# The association between area of residence and sufficient antenatal tetanus vaccination in women ages 15–49 in Afghanistan: an analysis of the 2015 DHS dataset

**DOI:** 10.1186/s41256-020-00180-1

**Published:** 2020-11-30

**Authors:** Jillian Sherley, Sam Newton

**Affiliations:** 1 London School of Hygiene & Tropical Medicine, University of London, London, UK; 2 School of Public Health, Kwame Nkrumah University of Science and Technology, Kumasi, Ghana

**Keywords:** Tetanus, Antenatal, Afghanistan, Rural, Urban

## Abstract

**Background:**

Neonatal tetanus (NT) is a deadly nervous system disorder that is endemic to Afghanistan. Administering sufficient doses of tetanus toxoid containing vaccine (TTCV) during pregnancy can pass antibodies to the fetus and therefore prevent NT. Using survey data, we investigated the association between area of residence (urban or rural) and sufficient antenatal TTCV coverage among women aged 15–49 years in Afghanistan during their most recent pregnancy in the past 5 years that resulted in a live birth. Mother’s education level was also assessed as a potential effect modifier.

**Methods:**

Secondary analysis was performed on data from the 2015 Afghanistan Demographic and Health Survey (AfDHS). The 2015 AfDHS was a nationally representative survey with participants selected in a stratified two-stage sample design from urban and rural areas across Afghanistan’s 34 provinces. Data were analyzed on 19,737 women ages 15–49 that had a live birth in the 5 years preceding the survey. The relationship between area of residence and sufficient antenatal TTCV was assessed in a multivariable logistic regression model, adjusting for several confounding variables.

**Results:**

55.1% (95% CI = 51.6–58.5%) of urban women and 53.9% (95% CI = 49.7–57.9%) of rural women had sufficient tetanus vaccination coverage in their most recent pregnancy. In multivariate analysis, there was strong evidence for greater odds of sufficient antenatal tetanus vaccination in rural areas (OR = 1.62; 95% CI = 1.18–2.24, *p* = 0.003). There was no effect modification on this association by mother’s education level.

**Conclusions:**

Women in rural areas of Afghanistan have greater odds of receiving sufficient antenatal tetanus vaccination than women in urban areas. Further study into factors contributing to this urban-rural disparity is needed. Targeted antenatal tetanus vaccination strategies for urban and rural women will be necessary as Afghanistan continues to work towards NT eradication.

**Supplementary Information:**

The online version contains supplementary material available at 10.1186/s41256-020-00180-1.

## Background

Tetanus is a serious nervous system infection caused by the anaerobic bacterium *Clostridium tetani*, which lives in the soil and cannot be eradicated because it is so ubiquitous in the environment. Neonatal tetanus (NT) is defined as developing tetanus within the first 28 days of life, and 90% will develop symptoms in the first 3–14 days [1]. NT is characterized by reduced feeding due to trismus, followed by widespread rigidity and muscle spasm, then autonomic nervous system dysfunction and respiratory failure [1]. Childbirth in contaminated environments using non-sterile instruments increases an infant’s exposure to tetanus, and babies born to mothers without complete tetanus toxoid-containing vaccine (TTCV) coverage are at particular risk [1]. NT remains endemic in many low and middle income countries (LMIC) where it has a virtual 100% mortality rate because the specialized care needed is often unavailable [1]. A fully vaccinated pregnant woman will pass antibodies through the placenta to the fetus, thus protecting against tetanus until the baby can be vaccinated at 6 weeks of age [2]. A systematic review by Blencowe et al. concluded proper antenatal TTCV coverage reduces NT mortality by 94% [2]. As of July 2019, NT remains endemic in 12 countries [3]. Most of these countries are in sub-Saharan Africa, with the exceptions of Afghanistan, Pakistan, Papua New Guinea and Yemen [3]. WHO’s Strategic Advisory Group of Experts on Immunization has declared 2020 the goal for global NT eradication [4].

When the Taliban regime collapsed in 2001, Afghanistan reported a dismal maternal mortality rate of 1390/100,000 live births and an infant mortality rate of 88/1000 live births [5]. In 2002 the Afghanistan Ministry of Public Health (MoPH) introduced the Basic Package of Health Services (BPHS) to provide standardized cost-effective healthcare services throughout the country, including widespread availability of antenatal TTCV [6]. By 2017, maternal and infant mortality had decreased to 638/100,000 live births and 49/1000 live births, respectively [5]. From 1990 to 2015 Afghanistan’s NT rate decreased markedly from 129/1000 live births to 16/1000 live births [7]. However, the actual value may be higher as complete ascertainment of NT cases can be challenging. Births and deaths occurring at home may be unreported [8] and NT deaths that occur shortly after birth are sometimes misreported as stillbirths [9]. NT is considered eradicated when there are < 1/1000 cases for all live births in every area of a country [10].

The Afghanistan MoPH provides a three dose primary TTCV series in infancy [11]. While many countries give subsequent booster doses at school, this would miss a large number of girls in Afghanistan, particularly in rural areas. Only 41 and 36% of girls in rural Afghanistan attend primary and secondary school, respectively [12]. Thus, women in Afghanistan are offered TTCV at their ANC (antenatal care) visits and/or during supplementary immunization activities (SIAs) [11]. In 2006, 2009, 2010 and 2013 the Afghanistan MoPH carried out SIAs in rural areas high-risk for NT, and received funding by UNICEF to conduct SIAs annually from 2015 to 19 [11].

Harsh winters and mountainous terrain in Afghanistan can present problems in delivering vaccines to healthcare facilities, particularly in remote locations [11]. As of 2016, only 65% of public health facilities providing ANC actually had TTCV in stock [13]. Difficulty in providing adequate antenatal TTCV also stems from a shortage of female healthcare workers. While the number of health workers trained in vaccinating nearly quadrupled between 2004 and 2014 [14], in 2018 only 31% of vaccinators were female and the majority of female vaccinators chose to work in secure, urban areas [15]. Since it is often considered unacceptable in Afghanistan for a woman to be immunized by a man [15], a pregnant woman in a rural area may have no choice but to remain unvaccinated.

This paper examines whether there is a difference in antenatal TTCV coverage between rural and urban area of residence among Afghan women during their most recent pregnancy resulting in a live birth. Variables that confound the association between area of residence and antenatal TTCV coverage will be identified and mother’s education level will be explored as a potential effect modifier. There are currently no published studies that examine this same association using data from Afghanistan, and given that Afghanistan remains one of the few countries in the world yet to eradicate NT, it is critical that reasons for this be explored. The purpose of this study is to fill a current gap in the literature and explore potential health system causes for any difference in TTCV coverage by area of residence in Afghanistan.

## Methods

### Study design and data source

**Fig. 1:**
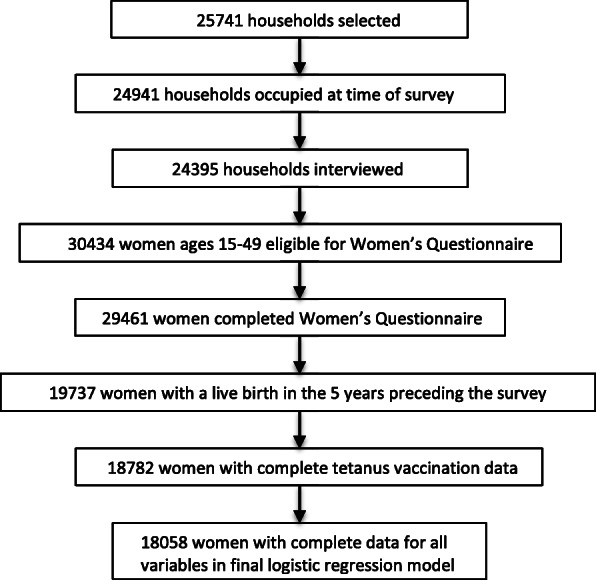
Flow chart of sample

A secondary analysis of the 2015 Afghanistan Demographic and Health Survey (AfDHS) was done to examine the association between a woman’s area of residence and sufficient antenatal TTCV coverage in her most recent pregnancy resulting in a live birth. The sampling frame for the 2015 AfDHS was the Household Listing Frame provided by the Central Statistics Organization (CSO), last updated in 2009. There were 25,974 enumeration areas across 34 provinces. Small provinces were oversampled to ensure the number surveyed was proportional to the size of the province. A stratified two-stage sample design was used, first selecting 950 clusters (690 rural, 260 urban) across the enumeration areas. Given the unstable security situation in some provinces, 101 reserve clusters were selected in case any intended clusters could not be reached. Next, an equal probability systematic selection process was used to select a fixed number of 27 households per cluster. A large number of clusters (70) were considered too unsafe to survey and thus all reserve clusters were used, resulting in 956 clusters being successfully surveyed, for a total of 24,941 households. The Women’s Questionnaire (Appendix A) of the 2015 AfDHS was administered to all ever-married women ages 15–49 who were either permanent residents of the household or visitors who stayed the night before. The questionnaire was first written in English and then translated into Pashto and Dari. As shown in Fig. 1, 18,782 women with complete information about their tetanus vaccination history were interviewed, and 18,058 women had complete data for all variables included in the final logistic regression model.

### Variables

The primary exposure was the woman’s area of residence (urban or rural). The outcome was whether or not a woman had sufficient antenatal TTCV coverage in her most recent pregnancy that resulted in a live birth in the 5 years preceding the survey. A woman was considered protected against tetanus if she had:

- Two TTCV during the most recent pregnancy, or
- Two or more TTCV, the latest within 3 years of the birth, or
- Three or more TTCV, the latest within 5 years of the birth, or
- Four or more TTCV, the latest within 10 years of the birth, or
- Five or more TTCV at any time

Several potential confounding variables were identified via a literature review: mother’s age group (15–19, 20–24, 25–29, 30–34, 35–39, 40–44, 45–49), marital status (married, widowed, divorced/separated), ethnicity (Pasthun, Tajik, Hazara, Uzbek, other), number of living children (none, 1–2, 3–4, 5–6, 7–8, 9+), birth order (1, 2, 3, 4, 5+), number of ANC visits (0, 1, 2, 3, 4+), mother’s highest education level (none, primary, secondary, higher), husband’s highest education level (none, primary, secondary, higher), mother’s employment status (employed, unemployed), husband’s type of employment (non-manual, skilled manual and unskilled manual), combined wealth index (poorest, poorer, middle, richer, richest), whether the woman wanted her last child (wanted then, wanted later, no further children were wanted), mother’s perception of distance to the health facility (big problem, not a big problem), mother’s perception of ease in getting permission for medical care (big problem, not a big problem).

### Data management and analysis

Statistical analyses were performed with Stata version 14.2. Prior to analysis, data was weighted and adjusted to account for oversampling in the smaller provinces and the stratified cluster sampling technique. Several variables had very small amounts of missing data (0.1–0.3%); data was assumed to be missing completely at random and multivariate analyses used only subjects with complete data. All covariates were considered potential confounders and included, in turn, in a bivariate logistic regression model with the exposure and outcome of interest. Variables that changed the crude odds ratio (OR) by at least 10% approximately were considered confounders and carried forward to the final multivariate logistic regression model. Mother’s age group was considered an a priori confounder. The final model was examined for effect modification by mother’s education level as both a categorical and binary variable (no education vs. some education) and was also checked for multicollinearity using the vif command.

## Results

### Baseline characteristics of participants

Baseline demographic and reproductive characteristics are presented in Table 1. A majority of women were between 20 and 34 years old (71.6%). Most women lived in rural areas (76.8%) and nearly all were married (99.1%). The vast majority of subjects did not work (88.9%). 82.9% of women were uneducated and 57.6% had uneducated husbands. Just over half (54.1%) had sufficient antenatal TTCV in their most recent pregnancy. Over one third (38.1%) did not receive any ANC. About half said it was a big problem to get permission to seek medical care and 68.1% said the distance to the health facility was a big problem.

**Table 1:** Baseline characteristics of women ages 15–49^a^, Afghanistan Demographic and Health Survey 2015

Variable	Category	N	Wt% (95% CI)^b^
Demographic characteristics			
Area of residence	Urban	4731	23.3 (20.6–26.2)
	Rural	15,006	76.8 (73.8–79.4)
Mother’s age (years)	15–19	862	4.4 (4.0–4.8)
	20–24	4825	25.3 (24.2–26.4)
	25–29	5701	28.6 (27.6–29.5)
	30–34	3632	17.7 (16.7–18.7)
	35–39	2856	15.1 (14.0–16.3)
	40–44	1288	5.8 (5.2–6.6)
	45–49	573	3.2 (2.8–3.7)
Marital status	Married	19,525	99.1 (98.9–99.3)
	Widowed	168	0.7 (0.6–1.0)
	Divorced/separated	44	0.02 (0.1–0.2)
Mother’s highest education level	None	16,787	82.9 (80.9–84.7)
	Primary	1401	8.1 (7.3–9.0)
	Secondary	1246	7.3 (6.2–8.6)
	Higher	303	1.7 (1.3–2.1)
Husband’s highest education level (*N* = 19,485)	None	11,011	57.6 (55.3–59.8)
	Primary	2713	14.8 (13.7–15.8)
	Secondary	4381	21.3 (19.8–22.8)
	Higher	1380	6.4 (5.7–7.3)
Mother’s employment status (*N* = 19,673)	Unemployed	17,649	88.9 (86.7–90.8)
	Employed	2024	11.1 (9.2–13.3)
Husband’s type of employment (*N* = 19,675)	Non-manual	13,373	65.3 (63.3–67.2)
	Skilled manual	2889	16.7 (14.5–19.0)
	Unskilled manual	3413	18.1 (16.7–19.5)
	Pashtun	8482	40.0 (36.0–44.1)
Ethnicity (*N* = 19,702)	Tajik	5820	32.3 (29.0–35.9)
	Hazara	1717	9.1 (6.8–12.1)
	Uzbek	1344	11.2 (7.3–16.9)
	Other	2339	7.4 (6.0–9.1)
Combined wealth index	Poorest	3721	19.9 (18.0–22.0)
	Poorer	4485	20.2 (18.7–21.8)
	Middle	4365	20.5 (18.5–22.6)
	Richer	4268	20.7 (18.6–22.9)
	Richest	2898	18.8 (16.6–21.2)
Reproductive characteristics			
Sufficient antenatal TTCV (*N* = 18,782)	No	9187	45.9 (42.6–49.1)
	Yes	9595	54.1 (50.9–57.4)
Number living children	None	116	5.5 (4.2–7.1)
	1–2	6374	33.6 (32.3–34.9)
	3–4	5966	30.2 (29.1–31.2)
	5–6	4108	20.4 (19.4–21.4)
	7–8	2204	10.8 (10.1–11.6)
	9+	969	4.6 (4.1–5.0)
Variable	Category	N	Wt% (95% CI)^b^
Birth order	1	2940	15.2 (14.3–16.2)
	2	3096	16.7 (15.7–17.9)
	3	2930	15.1 (14.4–15.8)
	4	2649	13.3 (12.5–14.2)
	5+	8122	40.0 (38.5–40.9)
Number ANC visits (*N* = 19,297)	None	8506	38.8 (35.6–42.1)
	1	2136	12.2 (11.2–13.3)
	2	3246	18.0 (16.6–19.6)
	3	2213	12.7 (11.2–14.4)
	4+	3196	18.2 (16.8–19.7)
Wanted last child (*N* = 19,716)	Wanted child then	17,776	89.0 (87.9–90.0)
	Wanted child later	1110	6.0 (5.4–6.7)
	Did not want	830	5.0 (4.3–5.9)
Distance to health facility (*N* = 19,690)	Big problem	13,527	68.1 (66.0–70.3)
	Not a big problem	6163	31.9 (29.8–34.0)
Getting permission to seek medical care (*N* = 19,688)	Big problem	11,499	51.3 (48.2–54.4)
	Not a big problem	8189	48.7 (45.6–51.8)

^a^*N* = 19,737 unless otherwise stated, ^b^Accounts for clustering and stratification in sample

### Associations between independent variables and outcome

Bivariate associations between area of residence and sufficient antenatal TTCV are shown in Table 2. A similar percentage of urban and rural women (55.1 and 53.9%, respectively) had sufficient TTCV in their most recent pregnancy (*p* = 0.655). Several variables were strongly positively associated with sufficient antenatal TTCV: mother’s age group, mother’s highest education level, husband’s highest education level, husband’s type of employment, ethnicity, combined wealth index, birth order, number ANC visits, distance to health facility and getting permission to seek medical care. Marital status and whether the last child was wanted were not associated with sufficient antenatal TTCV.

**Table 2:** Associations between various independent variables and a woman having sufficient TTCV in her most recent pregnancy resulting in a live birth within the past 5 years^a^

Variable	Category	N with sufficient antenatal TTCV	Wt% (95% CI)^**b**^ with sufficient antenatal TTCV	***P***-value^**c**^
**Demographic characteristics**				
Area of residence	Urban	2489	55.1 (51.6–58.5)	0.655
	Rural	7106	53.9 (49.7–57.9)	
Mother’s age group (years)	15–19	402	52.0 (45.8–58.1)	0.002
	20–24	2438	56.9 (52.5–61.3)	
	25–29	2820	54.5 (49.9–59.0)	
	30–34	1735	52.8 (49.0–56.6)	
	35–39	1395	55.6 (52.1–59.1)	
	40–44	560	47.3 (43.1–51.6)	
	45–49	245	44.7 (38.7–51.0)	
Marital status	Married	9,12	54.1 (50.9–57.4)	0.710
	Widowed	69	57.1 (42.8–70.2)	
	Divorced/separated	14	45.2 (25.0–67.2)	
Mother’s highest education level	None	7584	50.1 (46.9–53.3)	< 0.001
	Primary	897	69.3 (63.8–74.3)	
	Secondary	883	77.2 (70.4–82.8)	
	Higher	231	74.5 (62.2–83.9)	
Husband’s highest education level (*N* = 19,485)	None	4698	48.4 (44.3–52.5)	< 0.001
	Primary	1434	59.4 (55.0–63.7)	
	Secondary	2487	62.1 (58.3–65.7)	
	Higher	888	67.9 (62.6–72.7)	
Mother’s employment status (*N* = 19,673)	Unemployed	8739	46.0 (43.2–48.9)	0.778
	Employed	828	45.2 (37.7–52.9)	
Husband’s type of employment (*N* = 19,675)	Non-manual	6363	47.4 (43.9–51.0)	0.029
	Skilled manual	1514	40.1 (34.1–46.3)	
	Unskilled manual	1695	45.2 (41.3–49.1)	
Ethnicity (*N* = 19,702)	Pashtun	3886	47.0 (41.7–52.3)	< 0.001
	Tajik	3454	60.9 (57.8–63.9)	
	Hazara	842	51.7 (46.9–56.5)	
	Uzbek	748	67.3 (56.6–76.5)	
	Other	650	45.1 (40.8–50.0)	
Combined wealth index	Poorest	1794	53.1 (49.7–56.4)	0.016
	Poorer	1930	51.5 (47.5–55.5)	
	Middle	2023	50.0 (43.1–56.7)	
	Richer	2155	57.3 (52.5–62.0)	
	Richest	1693	59.4 (54.3–64.2)	
**Reproductive characteristics**				
Number living children	None	43	53.2 (41.1–64.8)	< 0.001
	1–2	3376	60.4 (56.9–63.8)	
	3–4	2868	53.2 (49.6–56.7)	
	5–6	1858	49.9 (45.5–54.4)	
	7–8	1003	47.2 (43.2–51.3)	
	9+	447	50.0 (42.9–57.1)	
Birth order	1	1575	61.6 (58.2–64.8)	< 0.001
	2	1636	59.5 (54.6–64.3)	
	3	1444	53.4 (49.3–57.9)	
	4	1245	52.3 (47.6–57.0)	
	5+	3695	49.8 (46.2–53.5)	
Number ANC visits (*N* = 19,297)	0	2465	34.1 (29.8–38.6)	< 0.001
	1	1008	50.8 (46.5–55.0)	
	2	1986	64.7 (62.1–67.2)	
	3	1576	74.2 (68.6–79.1)	
	4+	2318	72.4 (69.3–75.4)	
Wanted last child (*N* = 19,716)	Wanted child then	8609	54.5 (51.0–57.9)	0.541
	Wanted child later	581	51.2 (44.1–58.3)	
	Did not want	393	52.3 (45.7–58.8)	
Distance to health facility (*N* = 19,690)	Big problem	6345	51.6 (47.9–55.3)	< 0.001
	Not a big problem	3234	59.5 (55.8–63.1)	
Getting permission to seek medical care (*N* = 19,688)	Big problem	5125	48.9 (45.9–51.9)	< 0.001
	Not a big problem	4453	60.0 (55.2–63.8)	

^a^*N* = 19,737 unless otherwise stated, ^b^Accounts for clustering and stratification in sample, ^c^From Pearson’s chi-squared test

Combined wealth index (OR = 1.47; 95% CI = 1.00–2.15, *p* < 0.001), number of ANC visits (OR = 1.22; 95% CI = 1.01–1.48, *p* < 0.001), mother’s highest education level (OR = 1.17; 95% CI = 0.94–1.47, *p* < 0.001) and husband’s highest education level (OR = 1.08; 95% CI = 0.88–1.34, *p* < 0.001) were all confounders of the main association (Table 3). Two variables, distance to health facility (OR = 1.01; 95% CI = 0.81–1.26, *p* < 0.001) and getting permission to seek medical care (OR = 1.01; 95% CI = 0.81–1.27, *p* < 0.001), were weakly confounding. Marital status, ethnicity, mother’s employment, husband’s employment, number of living children, birth order and whether the last child was wanted were not confounders. Adjustment for mother’s age group changed the crude OR very minimally, but has been selected as an a priori confounder.

**Table 3:** Odds ratio of sufficient antenatal tetanus coverage in rural vs. urban area of residence with adjustment for potential confounders^a^

Adjustment	OR (95% CI) for sufficient antenatal	***P***-value^b^
**TTCV in rural vs. urban residence**		
None (crude OR)	0.93 (0.75–1.16)	0.525
Mother’s age group	0.94 (0.76–1.17)	< 0.001
Marital status	0.93 (0.75–1.16)	0.575
Mother’s highest education level	1.17 (0.94–1.47)	< 0.001
Husband highest education level	1.08 (0.88–1.34)	< 0.001
Mother employment status	0.93 (0.75–1.16)	0.655
Husband’s type of employment	0.96 (0.77–1.21)	< 0.001
Ethnicity	0.96 (0.79–1.16)	< 0.001
Combined wealth index	1.47 (1.00–2.15)	< 0.001
Number of living children	0.94 (0.75–1.17)	0.387
Birth order	0.94 (0.76–1.17)	0.411
Number ANC visits	1.22 (1.01–1.48)	< 0.001
Wanted last child	0.92 (0.73–1.14)	< 0.001
Distance to health facility	1.01 (0.81–1.26)	< 0.001
Getting permission to seek medical care	1.01 (0.81–1.27)	< 0.001

^a^*N* = 18,058; only cases with no missing values used for analysis, ^b^From F-test

### The association between confounding variables and exposure

Variables that confound the main association are all strongly inversely associated with area of residence (Table 4). Rural women have lower levels of education than urban women (*p* < 0.001); 33.8% of urban women had at least some education, compared to only 12.0% of rural women. Compared to urban women, rural women attend fewer ANC visits (*p* < 0.001), have husbands with lower levels of education (*p* < 0.001), have lower combined wealth index (*p* < 0.001), are more likely to state the distance to a health facility is a big problem (*p* < 0.001) and have more difficulty getting permission to seek medical care (*p* < 0.001).

**Table 4:** Associations between confounders of the main association and area of residence^a^

Variable	Category	Wt% (95% CI)^**b**^ inurban areas	Wt% (95% CI)^**b**^ in rural areas	***P***-value^**c**^
Mother’s highest education level	None	66.2 (62.7–69.4)	88.0 (85.5–90.1)	< 0.001
	Primary	13.9 (12.1–15.9)	6.4 (5.5–7.3)	
	Secondary	15.2 (13.1–17.6)	4.9 (3.7–6.6)	
	Higher	4.7 (3.4–6.6)	0.7 (0.5–1.2)	
Husband’s highest education level (*N* = 19,485)	None	40.2 (36.7–43.8)	62.8 (60.2–65.4)	< 0.001
	Primary	17.8 (16.2–19.5)	13.8 (12.6–15.2)	
	Secondary	28.7 (25.8–31.7)	19.0 (17.4–20.7)	
	Higher	13.4 (10.9–16.2)	4.4 (3.7–5.1)	
Number ANC visits (*N* = 19,297)	0	28.0 (24.5–31.7)	42.1 (38.0–46.3)	< 0.001
	1	11.4 (9.4–13.9)	12.5 (11.4–13.7)	
	2	14.2 (12.0–16.7)	19.2 (17.5–21.0)	
	3	14.1 (11.9–16.7)	12.3 (10.5–14.4)	
	4+	32.3 (29.1–35.8)	13.9 (12.5–15.4)	
Combined wealth index	Poorest	3.4 (1.8–6.4)	24.9 (22.4–27.7)	< 0.001
	Poorer	2.1 (1.4–3.3)	25.6 (23.7–27.7)	
	Middle	3.2 (2.4–4.1)	25.7 (23.4–28.2)	
	Richer	21.0 (16.9–25.7)	20.6 (18.2–23.2)	
	Richest	70.4 (64.6–75.5)	3.1 (1.9–5.2)	
Distance to health facility (*N* = 19,690)	Big problem	47.6 (42.9–52.4)	74.3 (71.7–76.8)	< 0.001
	Not a big problem	52.4 (47.6–57.1)	25.7 (23.2–28.4)	
Getting permission to seek medical care(*N* = 19,688)	Big problem	36.4 (33.6–39.2)	55.8 (51.6–59.9)	< 0.001
	Not a big problem	63.6 (60.8–66.4)	44.2 (40.1–48.4)	

^a^*N* = 19,737 unless otherwise stated, ^b^Accounts for clustering and stratification in sample, ^c^From Pearson’s chi-squared test

### The association between area of residence and antenatal TTCV

Data sparsity and multicollinearity were not present, and thus all confounders were included in the final multivariate regression model (Table 5). After adjusting for mother’s age group, number of ANC visits, combined wealth index, mother’s highest education level, husband’s highest education level, distance to health facility and getting permission to seek medical care, rural women had 62% greater odds of receiving sufficient antenatal TTCV compared to urban women (OR = 1.62; 95% CI = 1.18–2.24, *p* = 0.003). There was no effect modification by mother’s education as a categorical (*p* = 0.275) or binary (*p* = 0.148) variable, and thus stratified results are not presented.

**Table 5:** Logistic regression model for sufficient antenatal TTCV in women living in rural vs. urban areas, with adjustment for confounders^a^

	Unadjusted OR (95% CI)	***P***-value^b^	Adjusted OR (95% CI)^**c**^	***P***-value^b^
Area of residence		0.525		0.003
Urban	1.00		1.00	
Rural	0.93 (0.75–1.16)		1.62 (1.18–2.24)	
Mother’s age group		< 0.001		0.010
15–19	1.00		1.00	
20–24	0.85 (0.60–1.20)		1.26 (0.94–1.69)	
25–29	1.06 (0.76–1.49)		1.24 (0.97–1.60)	
30–34	0.86 (0.62–1.19)		1.29 (0.99–1.68)	
35–39	0.80 (0.57–1.13)		1.34 (0.99–1.83)	
40–44	0.95 (0.65–1.40)		0.99 (0.73–1.34)	
45–49	1.96 (1.15–3.36)		0.88 (0.58–1.34)	
Number ANC visits		< 0.001		< 0.001
None	1.00		1.00	
1	0.79 (0.56–1.12)		1.94 (1.55–2.42)	
2	0.87 (0.62–1.21)		3.31 (2.67–4.10)	
3	0.55 (0.39–0.77)		5.07 (3.87–6.64)	
4+	0.28 (0.21–0.37)		4.64 (3.69–5.84)	
Combined wealth index		< 0.001		0.264
Poorest	1.00		1.00	
Poorer	1.61 (0.73–3.53)		0.87 (0.72–1.05)	
Middle	1.14 (0.55–2.37)		0.77 (0.59–0.99)	
Richer	0.13 (0.06–0.27)		0.94 (0.73–1.20)	
Richest	0.01 (0.00–0.01)		0.89 (0.62–1.29)	
Mother’s highest education level^d^		< 0.001		< 0.001
None	1.00		1.00	
Primary	0.33 (0.26–0.41)		1.90 (1.45–2.49)	
Secondary	0.24 (0.16–0.34)		2.35 (1.62–3.41)	
Higher	0.12 (0.07–0.21)		1.46 (0.71–3.03)	
Husband’s highest education level		< 0.001		0.011
None	1.00		1.00	
Primary	0.49 (0.40–0.59)		1.20 (1.02–1.42)	
Secondary	0.40 (0.32–0.50)		1.29 (1.10–1.51)	
Higher	0.20 (0.15–0.28)		1.41 (1.03–1.93)	
Distance to health facility		< 0.001		0.249
Big problem	1.00		1.00	
Not a big problem	0.31 (0.24–0.38)		1.09 (0.94–1.28)	
Getting permission to seek medical care		< 0.001		0.001
Big problem	1.00		1.00	
Not a big problem	0.48 (0.39–0.59)		1.28 (1.11–1.49)	

^a^*N* = 18,058; only cases with no missing values used for analysis

^b^From adjusted Wald test

^c^Adjusted for area of residence, mother’s age group, number antenatal care visits, combined wealth index, mother’s highest education level, husband’s highest education level, distance to health facility and getting permission to seek medical care

^d^Adjusted Wald test for interaction *p* = 0.275

### Association between other independent variables and antenatal TTCV

In the final model (Table 5), women attending ANC visits had greater odds of sufficient antenatal TTCV than women who did not receive ANC (*p* < 0.001). Those attending four ANC visits had 4.6 times greater odds of sufficient antenatal TTCV coverage compared to women without any ANC (OR = 4.64; 95% CI = 3.69–5.84). Compared to no education, some maternal education (*p* < 0.001) and having a husband with some education (*p* = 0.011) were both strongly associated with greater odds of sufficient TTCV. Women ages 20–39 had greater odds of sufficient TTCV than 15–19 year olds, and women 40–44 and 45–49 had lower odds of sufficient TTCV than 15–19 year olds (*p* = 0.010). Women who said it was “not a big problem” to get permission for medical care had 28% greater odds of sufficient TTCV than women who did not have a problem getting permission (OR 1.28; 95% CI = 1.11–1.49, *p* = 0.001).

## Discussion

### Principal findings

Overall 54.1% of the women in the survey had sufficient TTCV coverage during their most recent pregnancy resulting in a live birth and 45.9% did not have sufficient TTCV coverage. This was slightly lower than the 2010 AfDHS, which reported 60% of pregnant women overall received sufficient TTCV [16]. After adjusting for confounders, the odds of sufficient antenatal tetanus protection were 62% greater in women living in rural areas compared to urban areas, and there was strong evidence for this association. The main association was not modified by the mother’s education level.

### Interpretation

There are no prior studies examining this association amongst Afghan women, and data from other LMIC is inconclusive. A secondary analysis of the 2008–2009 Kenya DHS [17] and a cross-sectional study from India [18] both concluded there was no difference in antenatal TTCV coverage between urban and rural women. A secondary analysis of the 2005–06 India DHS found rural women were 48% less likely to receive antenatal TTCV than urban women (*p* < 0.001), although a woman was considered protected if she received even one TTCV dose [19]. A cross-sectional study of Nigerian women found those in rural areas had 2.5 greater odds of insufficient TTCV compared to urban women (*p* < 0.001) [20].

A multivariate analysis from Turkey found rural women actually had nearly four times greater odds of receiving at least one dose of antenatal TTCV compared to urban women (*p* < 0.001) [21], The researchers concluded that urban ANC providers administered less antenatal TTCV than rural ANC providers, as they felt their patients were at minimal risk for tetanus infection due to likely delivering in a hospital [21]. A similar tendency is evident in Afghanistan from a 2016 survey on healthcare facilities, which found only 64% of ANC providers in Afghanistan knew that pregnant women should receive TTCV, with poorer knowledge in urban areas compared to rural areas (*p* < 0.001) [13]. Additionally, only 58% of urban health centres providing ANC in Afghanistan actually had TTCV in stock, compared to 69% of rural health centres [13]. Private hospitals, the vast majority of which are in urban areas, performed even more poorly, with only 53% carrying TTCV [13]. Most TTCV for pregnant women is administered during ANC visits. Despite 68% of urban women in our study receiving at least one ANC visit, compared to only 52% of rural women, urban women interestingly had lower odds of being sufficiently vaccinated. It therefore seems reasonable that in Afghanistan, like Turkey, pregnant women in urban areas, especially those attending private health facilities, may be offered antenatal TTCV less often than their rural counterparts due to less knowledgeable ANC providers and less vaccine availability. In 2016, 75 and 35% of rural and urban Afghan women, respectively, had home deliveries [22]. Thus, while home deliveries are more common in rural Afghanistan, there remains a substantial proportion of urban women also delivering at home. Failing to provide full antenatal TTCV coverage leaves the baby susceptible to tetanus infection until vaccination at 6 weeks of age, and also leaves the mother unprotected.

Differences in provision of vaccination services may also partially explain the higher odds of sufficient antenatal TTCV coverage in rural areas of Afghanistan, compared to urban areas. In effort to meet the WHO’s goal of globally eradicating NT by 2020, several rounds of yearly SIAs have been conducted in high-risk, remote areas of Afghanistan. In contrast, urban patients are generally expected to attend a fixed vaccination centre [11], which can be challenging for those without adequate transportation. Also, maintaining sufficient numbers of healthcare workers and vaccination supplies can be difficult in large urban catchment areas [11]. These factors taken together may increase TTCV availability for rural women, compared to urban women.

Demographic characteristics frequently associated with insufficient antenatal TTCV in the literature include a lower wealth index category [18, 23], the husband being unemployed [23], maternal unemployment [17, 18], lower maternal education level [8, 18, 24, 25] and younger maternal age [18, 21]. Reproductive characteristics often cited include fewer ANC visits [8, 21, 24, 25], less access to female healthcare workers [8, 18, 21, 26, 27], an undesired pregnancy [23, 27], lower birth order [18, 21, 24], not having permission to seek medical care [23], having to travel long distances to a health centre [26], not being told about pregnancy complications [23] and not having exposure to mass media that discusses tetanus vaccination [18, 23]. In our fully adjusted model, mothers 20–39 years old were more likely to be sufficiently vaccinated, compared to younger mothers (ages 15–19) and older mothers (ages 40–49). Young mothers have likely had fewer pregnancies and thus less opportunity to receive TTCV, and older mothers had previous pregnancies when antenatal TTCV was administered less often. Antenatal tetanus protection was more likely with an increasing number of ANC visits, as with a greater number of health care contacts comes a greater chance of being offered vaccination. Some education in both the mother and her husband, compared no education, was associated with greater odds of sufficient antenatal TTCV. Men and women with some education probably better understand the importance of protecting their baby against tetanus, and are thus more accepting of vaccination. Lastly, women who did not have issues getting permission to seek medical care were more likely to be sufficiently protected against tetanus. In a patriarchal society like Afghanistan, women permitted by their husbands to attend ANC would have more opportunities to be vaccinated. Combined wealth index was not associated with antenatal TTCV, likely because the Afghanistan MoPH provides tetanus free of charge to all pregnant women [11]. Distance to the nearest health facility was also not associated with sufficient tetanus vaccination, despite a majority (68.1%) of sampled women perceiving the distance as a “big problem.” Women in more remote areas from Afghanistan, who are probably a further distance from medical facilities, are also the same women benefitting from SIAs, which likely mitigates vaccine-access issues caused by distance.

Some previous studies have found effect modification by maternal education level. A secondary analysis of the 2004 Bangladesh DHS [23] showed highly educated women in rural areas were significantly more likely to receive two or more antenatal TTCV, compared to rural women with lower education levels, but there was no difference across education levels in urban areas. A summary of DHS data from 17 countries across Latin American, the Caribbean, sub-Saharan Africa, North Africa and Asia [28] found rural women with education were significantly more likely to receive antenatal TTCV than those without education, whereas urban women with education were often less likely to receive antenatal TTCV. In our sample, any effect modification by maternal education level was possibly mitigated by urban women being offered antenatal TTCV less by their ANC providers, either because the provider does not believe NT is a risk or because the vaccine is simply less available in some urban health centres, and also by increased TTCV availability through SIAs in rural areas.

### Strengths

The present study is the first to examine the association between area of residence and antenatal TTCV coverage in Afghanistan in a multivariate model. Although security concerns and difficult terrain in some areas of the country presented challenges to the AfDHS data collection, preselection of 101 reserve clusters ensured an adequate number of rural and urban clusters were ultimately surveyed. This is expected to make the findings generalizable to pregnant women ages 15–49 throughout Afghanistan. The AfDHS had an excellent response rate, with 97.8% of all selected households completing the survey and 96.8% of all eligible women ages 15–49 participating in the Women’s Questionnaire. There was also very little missing data, as 18,058/19737 (91.5%) of women had complete data sets. The high response rate and small amounts of missing data both greatly minimize selection bias. This study’s sample size was much greater than [8, 17, 23, 25, 26], or comparable to [28] many studies in other LMIC that examine antenatal TTCV coverage. The large sample size reduces the role of chance in producing the associations that were found.

In some previous studies a pregnancy was defined as protected if a woman received two TTCV during a given pregnancy. This can underestimate the proportion protected as vaccinations received earlier in life and during other pregnancies may afford a woman full protection, even if no vaccinations are received in the current pregnancy. Additionally some studies consider a woman protected even if only 1 antenatal TTCV was received, which could overestimate the proportion protected. For the present study, the definition of sufficient antenatal tetanus protection adhered as closely as possible to the WHO definition [4], given the data available, which ensured outcome estimates were as accurate as possible. Also, most previous studies examining urban-rural disparities in antenatal TTCV have only presented the relationship in an unadjusted bivariate model. The current study examined this association by producing a multivariate logistic regression model, which thus controlled for confounding.

### Limitations

#### Bias

As outcome data was based only on a woman’s report of her vaccination status, the possibility for information bias was introduced. Women were not asked to produce a vaccination card, and indeed many may not have one. In study of three Afghan provinces, only 29–68% of women were able to produce immunization cards when asked [9]. Thus, a study on antenatal tetanus vaccination may not be possible if cards were required to prove vaccination status. Hasnain et al. found maternal recall of TTCV is actually felt to be reasonably accurate as pregnancy is a unique event and medical interventions received during it are remembered clearly [25]. Two other studies have found that women actually tend toward underreporting their tetanus vaccination status. In Central African Republic, women’s recall placed tetanus immunity at 74.4%, whereas tetanus immunity via seroprevalance was higher at 88.7% [29]. In Bangladesh, women underreported number of tetanus vaccines if they had received more than two doses, or if a dose had been given more than 1 year prior [30]. In the present study, any outcome misclassification is likely to be non-differential according to area of residence, and thus the strength of the association between area of residence and antenatal TTCV coverage may be underestimated.

#### Residual confounding

The main association was examined for confounding by several variables that have been previously identified in studies from other LMIC as being associated with antenatal TTCV coverage. There may be residual confounding by variables not measured in the AfDHS data, and thus could not be included in this study. For instance, variables strongly associated with antenatal TTCV coverage in other studies that were not measured in AfDHS include access to female healthcare workers, [18, 21, 24, 26, 27] being told about pregnancy complications [23] and exposure to mass media that discusses tetanus vaccinations [18, 23]. Also, the type and location of ANC provider was examined by Haile et al. [17], although no association was found with antenatal TTCV coverage. The 2015 AfDHS did ask women about type and location of ANC provider, however each participant was permitted to list multiple different answers with no indication as to which was utilized most frequently, and thus it was not possible to include these variables in a meaningful way in this study.

## Conclusions

During their most recent pregnancy resulting in a live birth, Afghan women in rural areas had greater odds of sufficient antenatal tetanus vaccination than women in urban areas. This study further highlights the importance of verifying tetanus vaccination status in every pregnancy, in every region of the country, and providing tetanus vaccination where appropriate in order to finally eradicate NT in Afghanistan. Further exploration of the barriers that exist to antenatal tetanus vaccination in different regions of Afghanistan is needed, as is a coordinated national approach to increase the proportion of pregnant women sufficiently vaccinated against tetanus.

## Supplementary Information

**Additional file 1.**

## Data Availability

The data that support the findings of this study are available from the Demographic and Health Surveys (DHS) Program but restrictions apply to the availability of these data, which were used under license for the current study, and so are not publicly available. Data are however available from DHS upon reasonable request.

## Abbreviations

AfDHS: Afghanistan Demographic and Health Survey
ANC: Antenatal care
BPHS: Basic Package of Health Services
CSO: Central Statistics Organization
LMIC: Low and middle income countries
MoPH: Ministry of Public Health
NT: Neonatal tetanus
SIA: Supplementary Immunization Activity
TTCV: Tetanus toxoid containing vaccine
WHO: World Health Organization
