# Supplementary material for: The association between area of residence and sufficient antenatal tetanus vaccination in women ages 15–49 in Afghanistan: an analysis of the 2015 DHS dataset
Source: Glob Health Res Policy. 2020 Nov 30;5:51. doi: 10.1186/s41256-020-00180-1 (PMC7702667; doi:10.1186/s41256-020-00180-1)
Supplement: Supplementary file 1 — Additional file 1. [file 41256_2020_180_MOESM1_ESM.pdf]

01 May 2015

AFGHANISTAN DEMOGRAPHIC AND HEALTH SURVEY 2015

EVER-MARRIED WOMAN'S QUESTIONNAIRE

CENTRAL STATISTICS ORGANIZATION AND MINISTRY OF PUBLIC HEALTH

IDENTIFICATION

PROVINCE ____________________

DISTRICT ____________________

VILLAGE/NAHIA ____________________

CONTROLLER AREA . . . .

CLUSTER NUMBER [SAHA SHOMOR] . . . .

TYPE OF LOCATION (URBAN=1; RURAL=2) . . . .

STRUCTURE/BUILDING NUMBER/GATE NUMBER . . . .

HOUSEHOLD NUMBER . . . .

NAME OF HOUSEHOLD HEAD ____________________

NAME AND LINE NUMBER OF WOMAN ____________________

WOMAN SELECTED FOR DOMESTIC VIOLENCE MODULE (YES=1; NO=2)

INTERVIEWER VISITS

	1	2	3	FINAL VISIT
DATE	________	________	________	DAY<br>MONTH<br>YEAR
INTERVIEWER'S NAME	________	________	________	INT. NO.
RESULT*	________	________	________	RESULT
NEXT VISIT: DATE	________	________		TOTAL NUMBER OF VISITS
TIME	________	________		

*RESULT CODES:
- 1 COMPLETED
- 2 NOT AT HOME
- 3 POSTPONED
- 4 REFUSED
- 5 PARTLY COMPLETED
- 6 INCAPACITATED
- 7 OTHER ____________ (SPECIFY)

	DARI	PASHTO	OTHER
LANGUAGE OF INTERVIEW	1	2	6 ______
NATIVE LANGUAGE OF RESPONDENT	1	2	6 ______

	YES	NO
TRANSLATOR USED?	1	2

SUPERVISOR	FIELD EDITOR	OFFICE EDITOR	KEYED BY
NAME ________	NAME ________	________<br>NAME	________<br>NAME

SECTION 1. RESPONDENT'S BACKGROUND

INTRODUCTION AND CONSENT

INFORMED CONSENT

As-salamu alaykum. My name is ______________________________________. I am working with Central Statistics Organization. We are conducting a survey about health all over Afghanistan, which is conducted with the joint effort of the Ministry of Public Health and Central Statistics Organization. The information we collect will help the government to plan health services. Your household was selected for the survey. The questions usually take about 30 to 60 minutes. All of the answers you give will be confidential and will not be shared with anyone other than members of our survey team. You don't have to be in the survey, but we hope you will agree to answer the questions since your views are important. If I ask you any question you don't want to answer, just let me know and I will go on to the next question or you can stop the interview at any time.

In case you need more information about the survey, you may contact the person listed on the card that has already been given to your household.

Do you have any questions? May I begin the interview now?

SIGNATURE OF INTERVIEWER: ______________________________ DATE: ____________________

RESPONDENT AGREES TO BE INTERVIEWED . . . 1 ↓ RESPONDENT DOES NOT AGREE TO BE INTERVIEWED 2 → END

NO.	QUESTIONS AND FILTERS	CODING CATEGORIES	SKIP
101	RECORD THE TIME.	HOUR . . . . [ ][ ]<br>MINUTES . . . . [ ][ ]	
102	In what month and year were you born?	MONTH . . . . [ ][ ]<br>DON'T KNOW MONTH . . . . 98<br>YEAR . . . . [ ][ ][ ][ ]<br>DON'T KNOW YEAR 9998	
103	How old were you at your last birthday?<br>COMPARE AND CORRECT 102 AND/OR 103 IF INCONSISTENT.	AGE IN COMPLETED YEARS [ ][ ]	
104	Have you ever attended school?	YES . . . . 1<br>NO 2	 → 108
104A	What type of school (Madrassa) have you attended?	SCHOOL . . . . 1<br>MADRASSA 2	
105	What is the highest level of school you attended: primary, secondary, or higher?	PRIMARY . . . . 1<br>SECONDARY . . . . 2<br>HIGHER 3	
106	What is the highest grade you completed?<br>IF COMPLETED LESS THAN GRADE ONE, RECORD '00'.	GRADE . . . . [ ][ ]	
107	CHECK 105:<br>PRIMARY [ ] ↓ SECONDARY OR HIGHER [ ] →		110

NO.	QUESTIONS AND FILTERS	CODING CATEGORIES	SKIP
108	Now I would like you to read this sentence to me.<br><br>SHOW CARD TO RESPONDENT.<br><br>IF RESPONDENT CANNOT READ WHOLE SENTENCE, PROBE: Can you read any part of the sentence to me?	CANNOT READ AT ALL . . . . 1<br>ABLE TO READ ONLY PARTS OF SENTENCE . . . . 2<br>ABLE TO READ WHOLE SENTENCE 3<br>NO CARD WITH REQUIRED LANGUAGE ____________ 4<br>(SPECIFY LANGUAGE)<br>BLIND/VISUALLY IMPAIRED 5	
109	CHECK 108:<br>CODE '2', '3' OR '4' CIRCLED ☐ ↓ CODE '1' OR '5' CIRCLED ☐ →		111
110	Do you read a newspaper or magazine at least once a week, less than once a week or not at all?	AT LEAST ONCE A WEEK . . . . 1<br>LESS THAN ONCE A WEEK . . . . 2<br>NOT AT ALL 3	
111	Do you listen to the radio at least once a week, less than once a week or not at all?	AT LEAST ONCE A WEEK . . . . 1<br>LESS THAN ONCE A WEEK . . . . 2<br>NOT AT ALL 3	
112	Do you watch television at least once a week, less than once a week or not at all?	AT LEAST ONCE A WEEK . . . . 1<br>LESS THAN ONCE A WEEK . . . . 2<br>NOT AT ALL 3	
113	To which ethnic group do you belong?	PASHTUN . . . . 01<br>TAJIK . . . . 02<br>HAZARA . . . . 03<br>UZBEK . . . . 04<br>TURKMEN . . . . 05<br>NURISTANI . . . . 06<br>BALOCH . . . . 07<br>PASHAI . . . . 08<br><br>OTHER ____________ 96<br>(SPECIFY)	

SECTION 2. REPRODUCTION

NO.	QUESTIONS AND FILTERS	CODING CATEGORIES	SKIP
201	Now I would like to ask about all the births you have had during your life. Have you ever given birth?	YES . . . . 1<br>NO 2	 2 → 206
202	Do you have any sons or daughters to whom you have given birth who are now living with you?	YES . . . . 1<br>NO 2	 2 → 204
203	How many sons live with you?<br><br>And how many daughters live with you?<br><br>IF NONE, RECORD '00'.	SONS AT HOME . . . . [ ][ ]<br><br>DAUGHTERS AT HOME . . . . . [ ][ ]	
204	Do you have any sons or daughters to whom you have given birth who are alive but do not live with you?	YES . . . . 1<br>NO 2	 2 → 206
205	How many sons are alive but do not live with you?<br><br>And how many daughters are alive but do not live with you?<br><br>IF NONE, RECORD '00'.	SONS ELSEWHERE . . . . [ ][ ]<br><br>DAUGHTERS ELSEWHERE [ ][ ]	
206	Have you ever given birth to a boy or girl who was born alive but later died?<br><br>IF NO, PROBE: Any baby who cried or showed signs of life but did not survive?	YES . . . . 1<br>NO 2	 2 → 208
207	How many boys have died?<br><br>And how many girls have died?<br><br>IF NONE, RECORD '00'.	BOYS DEAD . . . . [ ][ ]<br><br>GIRLS DEAD . . . . [ ][ ]	
208	SUM ANSWERS TO 203, 205, AND 207, AND ENTER TOTAL.<br>IF NONE, RECORD '00'.	TOTAL BIRTHS . . . . [ ][ ]	
209	CHECK 208:<br><br>Just to make sure that I have this right: you have had in TOTAL _____ births during your life. Is that correct?<br><br>YES [ ] ↓ NO [ ] → PROBE AND CORRECT 201-208 AS NECESSARY.		
210	CHECK 208:<br><br>ONE OR MORE BIRTHS [ ] ↓ NO BIRTHS [ ] → 226		226

211 Now I would like to record the names of all your births, whether still alive or not, starting with the first one you had.
RECORD NAMES OF ALL THE BIRTHS IN 212. RECORD TWINS AND TRIPLETS ON SEPARATE ROWS.
(IF THERE ARE MORE THAN 12 BIRTHS, USE AN ADDITIONAL QUESTIONNAIRE, STARTING WITH THE SECOND ROW).

212<br>What name was given to your (first/next) baby?<br>RECORD NAME.<br>BIRTH HISTORY	213<br>Is (NAME) a boy or a girl?	214<br>Were any of these births twins?	215<br>In what month and year was (NAME) born?<br>PROBE: When is his/her birthday?	216<br>Is (NAME) still alive?	217<br>IF ALIVE:<br>How old was (NAME) at his/her last birthday?<br>RECORD AGE IN COM-PLETED YEARS.	218<br>IF ALIVE:<br>Is (NAME) living with you?	219<br>IF ALIVE:<br>RECORD HOUSE-HOLD LINE NUMBER OF CHILD (RECORD '00' IF CHILD NOT LISTED IN HOUSE-HOLD).	220<br>IF DEAD:<br>How old was (NAME) when he/she died?<br>IF '1 YR', PROBE: How many months old was (NAME)? RECORD DAYS IF LESS THAN 1 MONTH; MONTHS IF LESS THAN TWO YEARS; OR YEARS.	221<br>Were there any other live births between (NAME OF PREVIOUS BIRTH) and (NAME), including any children who died after birth?
01	BOY 1<br>GIRL 2	SING 1<br>MULT 2	MONTH<br>YEAR	YES . . 1<br>NO . . . 2<br>220	AGE IN YEARS	YES . . . 1<br>NO 2	HOUSEHOLD LINE NUMBER<br>(NEXT BIRTH)	DAYS . . . 1<br>MONTHS 2<br>YEARS . . 3	
02	BOY 1<br>GIRL 2	SING 1<br>MULT 2	MONTH<br>YEAR	YES . . 1<br>NO . . . 2<br>220	AGE IN YEARS	YES . . . 1<br>NO 2	HOUSEHOLD LINE NUMBER<br>(GO TO 221)	DAYS . . . 1<br>MONTHS 2<br>YEARS . . 3	YES . . . . 1<br>ADD BIRTH<br>NO . . . . . 2<br>NEXT BIRTH
03	BOY 1<br>GIRL 2	SING 1<br>MULT 2	MONTH<br>YEAR	YES . . 1<br>NO . . . 2<br>220	AGE IN YEARS	YES . . . 1<br>NO 2	HOUSEHOLD LINE NUMBER<br>(GO TO 221)	DAYS . . . 1<br>MONTHS 2<br>YEARS . . 3	YES . . . . 1<br>ADD BIRTH<br>NO . . . . . 2<br>NEXT BIRTH
04	BOY 1<br>GIRL 2	SING 1<br>MULT 2	MONTH<br>YEAR	YES . . 1<br>NO . . . 2<br>220	AGE IN YEARS	YES . . . 1<br>NO 2	HOUSEHOLD LINE NUMBER<br>(GO TO 221)	DAYS . . . 1<br>MONTHS 2<br>YEARS . . 3	YES . . . . 1<br>ADD BIRTH<br>NO . . . . . 2<br>NEXT BIRTH
05	BOY 1<br>GIRL 2	SING 1<br>MULT 2	MONTH<br>YEAR	YES . . 1<br>NO . . . 2<br>220	AGE IN YEARS	YES . . . 1<br>NO 2	HOUSEHOLD LINE NUMBER<br>(GO TO 221)	DAYS . . . 1<br>MONTHS 2<br>YEARS . . 3	YES . . . . 1<br>ADD BIRTH<br>NO . . . . . 2<br>NEXT BIRTH
06	BOY 1<br>GIRL 2	SING 1<br>MULT 2	MONTH<br>YEAR	YES . . 1<br>NO . . . 2<br>220	AGE IN YEARS	YES . . . 1<br>NO 2	HOUSEHOLD LINE NUMBER<br>(GO TO 221)	DAYS . . . 1<br>MONTHS 2<br>YEARS . . 3	YES . . . . 1<br>ADD BIRTH<br>NO . . . . . 2<br>NEXT BIRTH
07	BOY 1<br>GIRL 2	SING 1<br>MULT 2	MONTH<br>YEAR	YES . . 1<br>NO . . . 2<br>220	AGE IN YEARS	YES . . . 1<br>NO 2	HOUSEHOLD LINE NUMBER<br>(GO TO 221)	DAYS . . . 1<br>MONTHS 2<br>YEARS . . 3	YES . . . . 1<br>ADD BIRTH<br>NO . . . . . 2<br>NEXT BIRTH

212 What name was given to your next baby? RECORD NAME. BIRTH HISTORY NUMBER	213 Is (NAME) a boy or a girl?	214 Were any of these births twins?	215 In what month and year was (NAME) born? PROBE: When is his/her birthday?	216 Is (NAME) still alive?	217 IF ALIVE: How old was (NAME) at his/her last birthday? RECORD AGE IN COM-PLETED YEARS.	218 IF ALIVE: Is (NAME) living with you?	219 IF ALIVE: RECORD HOUSE-HOLD LINE NUMBER OF CHILD (RECORD '00' IF CHILD NOT LISTED IN HOUSE-HOLD).	220 IF DEAD: How old was (NAME) when he/she died? IF '1 YR', PROBE: How many months old was (NAME)? RECORD DAYS IF LESS THAN 1 MONTH; MONTHS IF LESS THAN TWO YEARS; OR YEARS.	221 Were there any other live births between (NAME OF PREVIOUS BIRTH) and (NAME), including any children who died after birth?
08	BOY 1<br>GIRL 2	SING 1<br>MULT 2	MONTH<br>YEAR	YES . . 1<br>NO . . . 2 → 220	AGE IN YEARS	YES . . . 1<br>NO 2	HOUSEHOLD LINE NUMBER<br>(GO TO 221)	DAYS . . . 1<br>MONTHS 2<br>YEARS . . 3	YES . . . . 1 ADD BIRTH<br>NO . . . . . 2 NEXT BIRTH
09	BOY 1<br>GIRL 2	SING 1<br>MULT 2	MONTH<br>YEAR	YES . . 1<br>NO . . . 2 → 220	AGE IN YEARS	YES . . . 1<br>NO 2	HOUSEHOLD LINE NUMBER<br>(GO TO 221)	DAYS . . . 1<br>MONTHS 2<br>YEARS . . 3	YES . . . . 1 ADD BIRTH<br>NO . . . . . 2 NEXT BIRTH
10	BOY 1<br>GIRL 2	SING 1<br>MULT 2	MONTH<br>YEAR	YES . . 1<br>NO . . . 2 → 220	AGE IN YEARS	YES . . . 1<br>NO 2	HOUSEHOLD LINE NUMBER<br>(GO TO 221)	DAYS . . . 1<br>MONTHS 2<br>YEARS . . 3	YES . . . . 1 ADD BIRTH<br>NO . . . . . 2 NEXT BIRTH
11	BOY 1<br>GIRL 2	SING 1<br>MULT 2	MONTH<br>YEAR	YES . . 1<br>NO . . . 2 → 220	AGE IN YEARS	YES . . . 1<br>NO 2	HOUSEHOLD LINE NUMBER<br>(GO TO 221)	DAYS . . . 1<br>MONTHS 2<br>YEARS . . 3	YES . . . . 1 ADD BIRTH<br>NO . . . . . 2 NEXT BIRTH
12	BOY 1<br>GIRL 2	SING 1<br>MULT 2	MONTH<br>YEAR	YES . . 1<br>NO . . . 2 → 220	AGE IN YEARS	YES . . . 1<br>NO 2	HOUSEHOLD LINE NUMBER<br>(GO TO 221)	DAYS . . . 1<br>MONTHS 2<br>YEARS . . 3	YES . . . . 1 ADD BIRTH<br>NO . . . . . 2 NEXT BIRTH

222	Have you had any live births since the birth of (NAME OF LAST BIRTH)? IF YES, RECORD BIRTH(S) IN TABLE.	YES . . . . 1<br>NO 2	
223	COMPARE 208 WITH NUMBER OF BIRTHS IN HISTORY ABOVE AND MARK:<br>NUMBERS ARE SAME ☐ NUMBERS ARE DIFFERENT ☐ → (PROBE AND RECONCILE)		
224	CHECK 215:<br>ENTER THE NUMBER OF BIRTHS IN 1389 OR LATER.	NUMBER OF BIRTHS . . . .<br>NONE 0	→ 226

NO.	QUESTIONS AND FILTERS	CODING CATEGORIES	SKIP
225	C FOR EACH BIRTH SINCE HAMMAL 1389, ENTER 'B' IN THE MONTH OF BIRTH IN THE CALENDAR. WRITE THE NAME OF THE CHILD TO THE LEFT OF THE 'B' CODE. FOR EACH BIRTH, ASK THE NUMBER OF MONTHS THE PREGNANCY LASTED AND RECORD 'P' IN EACH OF THE PRECEDING MONTHS ACCORDING TO THE DURATION OF PREGNANCY. (NOTE: THE NUMBER OF 'P's MUST BE ONE LESS THAN THE NUMBER OF MONTHS THAT THE PREGNANCY LASTED.)		
226	Are you pregnant now?	YES . . . 1<br>NO . . . 2<br>UNSURE . . . 8	 NO, UNSURE → 230
227	How many months pregnant are you?<br>RECORD NUMBER OF COMPLETED MONTHS.<br>C ENTER 'P's IN THE CALENDAR, BEGINNING WITH THE MONTH OF INTERVIEW AND FOR THE TOTAL NUMBER OF COMPLETED MONTHS.	MONTHS . . . [ ][ ]	
228	When you got pregnant, did you want to get pregnant at that time?	YES . . . 1<br>NO . . . 2	YES → 230
229	Did you want to have a baby later on or did you not want any (more) children?	LATER . . . 1<br>NO MORE . . . 2	
230	Have you ever had a pregnancy that miscarried, was aborted, or ended in a stillbirth?	YES . . . 1<br>NO . . . 2	 NO → 238
231	When did the last such pregnancy end?	MONTH . . . [ ][ ]<br>YEAR . . . [ ][ ][ ][ ]	
232	CHECK 231:<br>LAST PREGNANCY ENDED IN HAMMAL 1389 OR LATER [ ] ↓<br>LAST PREGNANCY ENDED BEFORE HAMMAL 1389 [ ] →		→ 238
233	How many months pregnant were you when the last such pregnancy ended?<br>C RECORD NUMBER OF COMPLETED MONTHS. ENTER 'T' IN THE CALENDAR IN THE MONTH THAT THE PREGNANCY TERMINATED AND 'P' FOR THE REMAINING NUMBER OF COMPLETED MONTHS.	MONTHS . . . [ ][ ]	
234	Since Hammal 1389, have you had any other pregnancies that did not result in a live birth?	YES . . . 1<br>NO . . . 2	 NO → 236
235	ASK THE DATE AND THE DURATION OF PREGNANCY FOR EACH EARLIER NON-LIVE BIRTH PREGNANCY BACK TO HAMMAL 1389.<br>C ENTER 'T' IN THE CALENDAR IN THE MONTH THAT EACH PREGNANCY TERMINATED AND 'P' FOR THE REMAINING NUMBER OF COMPLETED MONTHS.		
236	Did you have any miscarriages, abortions or stillbirths that ended before 1389?	YES . . . 1<br>NO . . . 2	 NO → 238
237	When did the last such pregnancy that terminated before 1389 end?	MONTH . . . [ ][ ]<br>YEAR . . . [ ][ ][ ][ ]	

NO.	QUESTIONS AND FILTERS	CODING CATEGORIES	SKIP
238	When did your last menstrual period start?<br><br>______________________<br>(DATE, IF GIVEN)	DAYS AGO . . . . 1 [ ][ ]<br>WEEKS AGO . . . . 2 [ ][ ]<br>MONTHS AGO . . . . 3 [ ][ ]<br>YEARS AGO . . . . 4 [ ][ ]<br>IN MENOPAUSE/<br>HAS HAD HYSTERECTOMY . . . 994<br>BEFORE LAST BIRTH . . . . 995<br>NEVER MENSTRUATED 996	
239	From one menstrual period to the next, are there certain days when a woman is more likely to become pregnant?	YES . . . . 1<br>NO . . . . 2<br>DON'T KNOW 8	2, 8 → 301
240	Is this time just before her period begins, during her period, right after her period has ended, or halfway between two periods?	JUST BEFORE HER PERIOD<br>BEGINS . . . . 1<br>DURING HER PERIOD . . . . 2<br>RIGHT AFTER HER<br>PERIOD HAS ENDED . . . . 3<br>HALFWAY BETWEEN<br>TWO PERIODS . . . . 4<br>OTHER ______________________ 6<br>(SPECIFY)<br>DON'T KNOW 8	

301	Now I would like to talk about family planning - the various ways or methods that a couple can use to delay or avoid a pregnancy.<br><br>Have you ever heard of (METHOD)?		
01	**Female Sterilization**. PROBE: Women can have an operation to avoid having any more children.	YES . . . . . 1<br>NO 2	
02	**Male Sterilization**. PROBE: Men can have an operation to avoid having any more children.	YES . . . . . 1<br>NO 2	
03	**IUD**. PROBE: Women can have a loop or coil placed inside them by a doctor or a nurse.	YES . . . . . 1<br>NO 2	
04	**Injectables**. PROBE: Women can have an injection by a health provider that stops them from becoming pregnant for one or more months.	YES . . . . . 1<br>NO 2	
05	**Implants**. PROBE: Women can have one or more small rods placed in their upper arm by a doctor or nurse which can prevent pregnancy for one or more years.	YES . . . . . 1<br>NO 2	
06	**Pill**. PROBE: Women can take a pill every day to avoid becoming pregnant.	YES . . . . . 1<br>NO 2	
07	**Male condom**. PROBE: Men can put a rubber sheath on their penis before sexual intercourse.	YES . . . . . 1<br>NO 2	
09	**Lactational Amenorrhea Method (LAM)**.	YES . . . . . 1<br>NO 2	
10	**Rhythm Method**. PROBE: To avoid pregnancy, women do not have sexual intercourse on the days of the month they think they can get pregnant.	YES . . . . . 1<br>NO 2	
11	**Withdrawal**. PROBE: Men can be careful and pull out before climax.	YES . . . . . 1<br>NO 2	
12	**Emergency Contraception**. PROBE: As an emergency measure, within three days after they have unprotected sexual intercourse, women can take special pills to prevent pregnancy.	YES . . . . . 1<br>NO 2	
13	Have you heard of any other ways or methods that women or men can use to avoid pregnancy?	YES . . . . . 1<br>____________<br>(SPECIFY)<br>____________<br>(SPECIFY)<br>NO 2	
302	CHECK 226:<br>NOT PREGNANT OR UNSURE ☐ ↓ PREGNANT ☐ → 311		311
303	Are you currently doing something or using any method to delay or avoid getting pregnant?	YES . . . . . 1<br>NO 2	 → 311

NO.	QUESTIONS AND FILTERS	CODING CATEGORIES	SKIP
304	Which method are you using?<br><br>CIRCLE ALL MENTIONED.<br><br>IF MORE THAN ONE METHOD MENTIONED, FOLLOW SKIP INSTRUCTION FOR HIGHEST METHOD IN LIST.	FEMALE STERILIZATION . . . . A<br>MALE STERILIZATION . . . . B<br>IUD . . . . C<br>INJECTABLES . . . . D<br>IMPLANTS . . . . E<br>PILL . . . . F<br>MALE CONDOM . . . . G<br>LACTATIONAL AMEN. METHOD . . . . . K<br>RHYTHM METHOD . . . . L<br>WITHDRAWAL . . . . M<br>OTHER MODERN METHOD . . . . X<br>OTHER TRADITIONAL METHOD . . . Y	A, B → 307<br>C, D, E → 308A<br>G → 306<br>K, L, M, X, Y → 308A
305	What is the brand name of the pills you are using?<br><br>IF DON'T KNOW THE BRAND, ASK TO SEE THE PACKAGE.	CONTRACEPTIVE LD . . . . 01<br>NOVA . . . . 02<br>CONTRACEPTIVE HD . . . . 03<br>LO FEMENAL . . . . 04<br>MICROGYNON (SMP) . . . . 05<br>FAMILIA 28 . . . . 06<br>LYNESTRENOL . . . . 07<br>KHOSHI . . . . 08<br><br>OTHER ______________ 96<br>(SPECIFY)<br><br>DON'T KNOW 98	→ 308A
306	What is the brand name of the condoms you are using?<br><br>IF DON'T KNOW THE BRAND, ASK TO SEE THE PACKAGE.	ARAMESH . . . . 01<br>SATHI . . . . 02<br>ASODAGI . . . . 03<br>MOH/UNFPA . . . . 04<br>OTHER ______________ 96<br>(SPECIFY)<br><br>DON'T KNOW 98	→ 308A
307	In what facility did the sterilization take place?<br><br>PROBE TO IDENTIFY THE TYPE OF SOURCE.<br><br>IF UNABLE TO DETERMINE IF PUBLIC OR PRIVATE SECTOR, WRITE THE NAME OF THE PLACE.<br><br>______________________________<br>(NAME OF PLACE)	PUBLIC SECTOR<br>GOVT. HOSPITAL (NATIONAL, REGIONAL, PROVINCIAL OR DISTRICT) . . . . 11<br><br>OTHER PUBLIC SECTOR ______________ 16<br>(SPECIFY)<br><br>PRIVATE MEDICAL SECTOR<br>PRIVATE HOSPITAL/CLINIC . . . . . 21<br>PRIVATE DOCTOR'S OFFICE . . . . . 22<br>OTHER PRIVATE MEDICAL SECTOR ______________ 26<br>(SPECIFY)<br><br>OTHER ______________ 96<br>(SPECIFY)<br>DON'T KNOW 98	

NO.	QUESTIONS AND FILTERS	CODING CATEGORIES	SKIP
308	In what month and year was the sterilization performed?		
308A	Since what month and year have you been using (CURRENT METHOD) without stopping?<br><br>PROBE: For how long have you been using (CURRENT METHOD) now without stopping?	MONTH . . . . [ ][ ]<br>YEAR . . . . [ ][ ][ ][ ]	
309	CHECK 308/308A, 215 AND 231:<br>ANY BIRTH OR PREGNANCY TERMINATION AFTER MONTH AND YEAR OF START OF USE OF CONTRACEPTION IN 308/308A — YES / NO<br>GO BACK TO 308/308A, PROBE AND RECORD MONTH AND YEAR AT START OF CONTINUOUS USE OF CURRENT METHOD (MUST BE AFTER LAST BIRTH OR PREGNANCY TERMINATION).		
310	CHECK 308/308A:<br>YEAR IS 1389 OR LATER: C ENTER CODE FOR METHOD USED IN MONTH OF INTERVIEW IN THE CALENDAR AND IN EACH MONTH BACK TO THE DATE STARTED USING.<br>YEAR IS 1388 OR EARLIER: C ENTER CODE FOR METHOD USED IN MONTH OF INTERVIEW IN THE CALENDAR AND EACH MONTH BACK TO HAMMAL 1389.<br>THEN SKIP TO → 322		
311	I would like to ask you some questions about the times you or your partner may have used a method to avoid getting pregnant during the last few years.<br><br>USE CALENDAR TO PROBE FOR EARLIER PERIODS OF USE AND NONUSE, STARTING WITH MOST RECENT USE, BACK TO HAMMAL 1389.<br>USE NAMES OF CHILDREN, DATES OF BIRTH, AND PERIODS OF PREGNANCY AS REFERENCE POINTS.<br><br>C **IN COLUMN 1**, ENTER METHOD USE CODE OR '0' FOR NONUSE IN EACH BLANK MONTH.<br><br>ILLUSTRATIVE QUESTIONS:<br>* When was the last time you used a method? Which method was that?<br>* When did you start using that method? How long after the birth of (NAME)?<br>* How long did you use the method then?<br><br>**IN COLUMN 2**, ENTER CODES FOR DISCONTINUATION NEXT TO THE LAST MONTH OF USE. NUMBER OF CODES IN COLUMN 2 MUST BE SAME AS NUMBER OF INTERRUPTIONS OF METHOD USE IN COLUMN 1.<br><br>ASK WHY SHE STOPPED USING THE METHOD. IF A PREGNANCY FOLLOWED, ASK WHETHER SHE BECAME PREGNANT UNINTENTIONALLY WHILE USING THE METHOD OR DELIBERATELY STOPPED TO GET PREGNANT.<br><br>ILLUSTRATIVE QUESTIONS:<br>* Why did you stop using the (METHOD)? Did you become pregnant while using (METHOD), or did you stop to get pregnant, or did you stop for some other reason?<br>* IF DELIBERATELY STOPPED TO BECOME PREGNANT, ASK: How many months did it take you to get pregnant after you stopped using (METHOD)? AND ENTER ‘0’ IN EACH SUCH MONTH IN COLUMN 1.		

NO.	QUESTIONS AND FILTERS	CODING CATEGORIES	SKIP
312	CHECK THE CALENDAR FOR USE OF ANY CONTRACEPTIVE METHOD IN ANY MONTH<br><br>NO METHOD USED ☐ ↓ ANY METHOD USED ☐ → 314		→ 314
313	Have you ever used anything or tried in any way to delay or avoid getting pregnant?	YES . . . 1<br>NO . . . 2	 → 324
314	CHECK 304:<br><br>CIRCLE METHOD CODE:<br><br>IF MORE THAN ONE METHOD CODE CIRCLED IN 304, CIRCLE CODE FOR HIGHEST METHOD IN LIST.	NO CODE CIRCLED . . . 00<br>FEMALE STERILIZATION . . . 01<br>MALE STERILIZATION . . . 02<br>IUD . . . 03<br>INJECTABLES . . . 04<br>IMPLANTS . . . 05<br>PILL . . . 06<br>MALE CONDOM . . . 07<br>LACTATIONAL AMEN. METHOD . . . 11<br>RHYTHM METHOD . . . 12<br>WITHDRAWAL . . . 13<br>OTHER MODERN METHOD . . . 95<br>OTHER TRADITIONAL METHOD . . . 96	→ 324<br>→ 317A<br>→ 326<br><br><br><br><br><br>→ 315A (11, 12)<br><br>→ 326 (13, 95, 96)
315<br><br><br><br><br><br><br><br><br><br><br><br>315A	You first started using (CURRENT METHOD) in (DATE FROM 308/308A). Where did you get it at that time?<br><br><br><br><br><br><br><br><br><br><br><br>Where did you learn how to use the rhythm/lactational amenorrhea method?<br><br><br><br><br><br>PROBE TO IDENTIFY THE TYPE OF SOURCE.<br><br>IF UNABLE TO DETERMINE IF PUBLIC OR PRIVATE SECTOR, WRITE THE NAME OF THE PLACE.<br><br>__________<br>(NAME OF PLACE)	PUBLIC SECTOR<br>GOVT. HOSPITAL (NATIONAL, REGIONAL, PROVINCIAL OR DISTRICT) . . . 11<br>CHC/POLYCLINIC . . . 12<br>BASIC HEALTH CENTER . . . 13<br>HEALTH SUB-CENTER . . . 14<br>HEALTH POST/SUB-HEALTH POST 15<br>COMMUNITY HEALTH WORKER . . . 16<br>MOBILE CLINIC . . . 17<br>OTHER PUBLIC SECTOR ______ 18<br>(SPECIFY)<br>NON-GOVERNMENT SECTOR<br>MARIE STOPES . . . 21<br>RED CROSS SOCIETY . . . 22<br>AFGA . . . 23<br>OTHER NGO SECTOR ______ 26<br>(SPECIFY)<br><br>PRIVATE MEDICAL SECTOR<br>PRIVATE HOSPITAL/CLINIC . . . 31<br>PHARMACY . . . 32<br>PRIVATE DOCTOR . . . 33<br>FIELDWORKER . . . 34<br>OTHER PRIVATE MEDICAL SECTOR ______ 36<br>(SPECIFY)<br><br>OTHER SOURCE<br>CHARITY FOUNDATION . . . 41<br>REFUGEE CAMP . . . 42<br>SHOP . . . 43<br>FRIEND/RELATIVE . . . 44<br><br>OTHER ______ 96<br>(SPECIFY)	

NO.	QUESTIONS AND FILTERS	CODING CATEGORIES	SKIP
316	CHECK 304:<br><br>CIRCLE METHOD CODE:<br><br>IF MORE THAN ONE METHOD CODE CIRCLED IN 304, CIRCLE CODE FOR HIGHEST METHOD IN LIST.	IUD . . . . . . 03<br>INJECTABLES . . . . . . 04<br>IMPLANTS . . . . . . 05<br>PILL . . . . . . 06<br>MALE CONDOM . . . . . . 07<br>LACTATIONAL AMEN. METHOD . . . . . 11<br>RHYTHM METHOD 12	 <br><br><br>→ 323<br>→ 326<br>→ 326
317	At that time, were you told about side effects or problems you might have with the method?	YES . . . . . . 1<br>NO 2	→ 319<br>→ 318
317A	When you got sterilized, were you told about side effects or problems you might have with the method?	YES . . . . . . 1<br>NO 2	→ 319
318	Were you ever told by a health or family planning worker about side effects or problems you might have with the method?	YES . . . . . . 1<br>NO 2	 → 320
319	Were you told what to do if you experienced side effects or problems?	YES . . . . . . 1<br>NO 2	
320	CHECK 317:<br><br>CODE '1' CIRCLED ↓<br>At that time, were you told about other methods of family planning that you could use?<br><br>CODE '1' NOT CIRCLED ↓<br>When you obtained (CURRENT METHOD FROM 314) from (SOURCE OF METHOD FROM 307 OR 315), were you told about other methods of family planning that you could use?	YES . . . . . . 1<br>NO 2	→ 322
321	Were you ever told by a health or family planning worker about other methods of family planning that you could use?	YES . . . . . . 1<br>NO 2	
322	CHECK 304:<br><br>CIRCLE METHOD CODE:<br><br>IF MORE THAN ONE METHOD CODE CIRCLED IN 304, CIRCLE CODE FOR HIGHEST METHOD IN LIST.	FEMALE STERILIZATION . . . . . . 01<br>MALE STERILIZATION . . . . . . 02<br>IUD . . . . . . 03<br>INJECTABLES . . . . . . 04<br>IMPLANTS . . . . . . 05<br>PILL . . . . . . 06<br>MALE CONDOM . . . . . . 07<br>LACTATIONAL AMEN. METHOD . . . . . 11<br>RHYTHM METHOD . . . . . . 12<br>WITHDRAWAL . . . . . . 13<br>OTHER MODERN METHOD . . . . . . 95<br>OTHER TRADITIONAL METHOD . . . 96	(01, 02) → 326<br><br>(11, 12, 13, 95, 96) → 326

NO.	QUESTIONS AND FILTERS	CODING CATEGORIES	SKIP
323	Where did you obtain (CURRENT METHOD) the last time?<br><br>PROBE TO IDENTIFY THE TYPE OF SOURCE.<br><br>IF UNABLE TO DETERMINE IF PUBLIC OR PRIVATE SECTOR, WRITE THE NAME OF THE PLACE.<br><br>____________________<br>(NAME OF PLACE)	PUBLIC SECTOR<br>GOVT. HOSPITAL (NATIONAL, REGIONAL, PROVINCIAL OR DISTRICT) . . . . 11<br>CHC/POLYCLINIC . . . . 12<br>BASIC HEALTH CENTER . . . . 13<br>HEALTH SUB-CENTER . . . . 14<br>HEALTH POST/SUB-HEALTH POST 15<br>COMMUNITY HEALTH WORKER . . . 16<br>MOBILE CLINIC . . . . 17<br><br>OTHER PUBLIC SECTOR __________ 18<br>(SPECIFY)<br><br>NON-GOVERNMENT SECTOR<br>MARIE STOPES . . . . 21<br>RED CROSS SOCIETY . . . . 22<br>AFGA . . . . 23<br>OTHER NGO SECTOR __________ 26<br>(SPECIFY)<br><br>PRIVATE MEDICAL SECTOR<br>PRIVATE HOSPITAL/CLINIC . . . . . 31<br>PHARMACY . . . . 32<br>PRIVATE DOCTOR . . . . 33<br>FIELDWORKER . . . . 34<br>OTHER PRIVATE MEDICAL SECTOR __________ 36<br>(SPECIFY)<br><br>OTHER SOURCE<br>CHARITY/FOUNDATIONS . . . . 41<br>REFUGEE CAMP . . . . 42<br>SHOP . . . . 43<br>FRIEND/RELATIVE . . . . 44<br><br>OTHER __________ 96<br>(SPECIFY)	→ 326
324	Do you know of a place where you can obtain a method of family planning?	YES . . . . 1<br>NO 2	 → 326

NO.	QUESTIONS AND FILTERS	CODING CATEGORIES	SKIP
325	Where is that?<br><br>Any other place?<br><br>PROBE TO IDENTIFY EACH TYPE OF SOURCE.<br><br>IF UNABLE TO DETERMINE IF PUBLIC OR PRIVATE SECTOR, WRITE THE NAME OF THE PLACE.<br><br>________________<br>(NAME OF PLACE(S))	PUBLIC SECTOR<br>GOVT. HOSPITAL (NATIONAL, REGIONAL, PROVINCIAL OR DISTRICT) . . . . A<br>CHC/POLYCLINIC . . . . B<br>BASIC HEALTH CENTER . . . . C<br>HEALTH SUB-CENTER . . . . D<br>HEALTH POST/SUB-HEALTH POST E<br>COMMUNITY HEALTH WORKER . . . F<br>MOBILE CLINIC . . . . G<br>OTHER PUBLIC SECTOR __________ H<br>(SPECIFY)<br><br>NON-GOVERNMENT SECTOR<br>MARIE STOPES . . . . I<br>RED CROSS SOCIETY . . . . J<br>AFGA . . . . K<br>OTHER NGO SECTOR __________ L<br>(SPECIFY)<br><br>PRIVATE MEDICAL SECTOR<br>PRIVATE HOSPITAL/CLINIC . . . . . M<br>PHARMACY . . . . N<br>PRIVATE DOCTOR . . . . O<br>FIELDWORKER . . . . P<br>OTHER PRIVATE MEDICAL SECTOR __________ Q<br>(SPECIFY)<br><br>OTHER SOURCE<br>CHARITY/FOUNDATIONS . . . . R<br>REFUGEE CAMP . . . . S<br>SHOP . . . . T<br>FRIEND/RELATIVE . . . . U<br><br>OTHER __________ X<br>(SPECIFY)	
326	In the last 12 months, were you visited by community health worker who talked to you about family planning?	YES . . . . 1<br>NO 2	
327	In the last 12 months, have you visited a health facility for care for yourself (or your children)?	YES . . . . 1<br>NO 2	 → 401
328	Did any staff member at the health facility speak to you about family planning methods?	YES . . . . 1<br>NO 2	

401	CHECK 224:<br>ONE OR MORE BIRTHS IN 1389 OR LATER [ ] ↓<br>NO BIRTHS IN 1389 OR LATER [ ] → 556			
402	CHECK 215: ENTER IN THE TABLE THE BIRTH HISTORY NUMBER, NAME, AND SURVIVAL STATUS OF EACH BIRTH IN 1389 OR LATER. ASK THE QUESTIONS ABOUT ALL OF THESE BIRTHS. BEGIN WITH THE LAST BIRTH. (IF THERE ARE MORE THAN 3 BIRTHS, USE LAST 2 COLUMNS OF ADDITIONAL QUESTIONNAIRES).<br><br>Now I would like to ask some questions about your children born in the last five years. (We will talk about each separately.)			
403	BIRTH HISTORY NUMBER FROM 212 IN BIRTH HISTORY	LAST BIRTH<br>BIRTH HISTORY NUMBER [ ][ ]	NEXT-TO-LAST BIRTH<br>BIRTH HISTORY NUMBER [ ][ ]	SECOND-FROM-LAST BIRTH<br>BIRTH HISTORY NUMBER [ ][ ]
404	FROM 212 AND 216	NAME ____________<br>LIVING [ ] DEAD [ ]	NAME ____________<br>LIVING [ ] DEAD [ ]	NAME ____________<br>LIVING [ ] DEAD [ ]
405	When you got pregnant with (NAME), did you want to get pregnant at that time?	YES . . . . 1<br>(SKIP TO 408)<br>NO 2	YES . . . . 1<br>(SKIP TO 430)<br>NO 2	YES . . . . 1<br>(SKIP TO 430)<br>NO 2
406	Did you want to have a baby later on, or did you not want any (more) children?	LATER . . . . 1<br>NO MORE . . . . 2<br>(SKIP TO 408)	LATER . . . . 1<br>NO MORE . . . . 2<br>(SKIP TO 430)	LATER . . . . 1<br>NO MORE . . . . 2<br>(SKIP TO 430)
407	How much longer did you want to wait?	MONTHS . . 1 [ ][ ]<br>YEARS . . 2 [ ][ ]<br>DON'T KNOW 998	MONTHS . . 1 [ ][ ]<br>YEARS . . 2 [ ][ ]<br>DON'T KNOW . . . 998	MONTHS . . 1 [ ][ ]<br>YEARS . . 2 [ ][ ]<br>DON'T KNOW . . . 998
408	Did you see anyone for antenatal care for this pregnancy?	YES . . . . 1<br>NO . . . . 2<br>(SKIP TO 415)		
409	Whom did you see?<br><br>Anyone else?<br><br>PROBE TO IDENTIFY EACH TYPE<br><br>OF PERSON AND RECORD ALL MENTIONED.	HEALTH PERSONNEL<br>DOCTOR . . . . A<br>NURSE/MIDWIFE . . B<br>AUXILIARY MIDWIFE . . . . C<br><br>OTHER PERSON<br>TRADITIONAL BIRTH ATTENDANT . . D<br>COMMUNITY HEALTH WORKER . . . . E<br><br>OTHER____________ X<br>(SPECIFY)		

NO.	QUESTIONS AND FILTERS	LAST BIRTH<br>NAME ______	NEXT-TO-LAST BIRTH<br>NAME ______	SECOND-FROM-LAST BIRTH<br>NAME ______
410	Where did you receive antenatal care for this pregnancy?<br><br>Anywhere else?<br><br>PROBE TO IDENTIFY EACH TYPE OF SOURCE.<br><br>IF UNABLE TO DETERMINE IF PUBLIC OR PRIVATE<br><br>SECTOR, WRITE THE NAME OF THE PLACE.<br><br>______<br>(NAME OF PLACE(S))	HOME<br>YOUR HOME . . . . A<br>OTHER HOME . . . . B<br><br>PUBLIC SECTOR<br>GOVT. HOSPITAL C<br>CHC/POLY-<br>CLINIC . . . . D<br>BASIC HEALTH<br>CENTER . . . . E<br>HEALTH SUB-CENTEF F<br>HP/SHP . . . . G<br>CHW . . . . H<br>MOBILE CLINIC . . I<br>OTHER PUBLIC<br>SECTOR<br>______ J<br>(SPECIFY)<br><br>NGO SECTOR<br>MARIE STOPES . . . . K<br>RED CROSS . . . . L<br>AFGA . . . . M<br>OTHER NGO<br>SECTOR<br>______ N<br>(SPECIFY)<br><br>PRIVATE MED. SECTOR<br>PVT. HOSPITAL/<br>CLINIC . . . . O<br>PRIVATE DOCTOR . . P<br>OTHER PRIVATE<br>MED. SECTOR<br>______ Q<br>(SPECIFY)<br><br>OTHER SOURCE<br>CHARITY/<br>FOUNDATIONS . . R<br>REFUGEE CAMP . . S<br><br>OTHER______ X<br>(SPECIFY)		
411	How many months pregnant were you when you first received antenatal care for this pregnancy?	MONTHS . . . . [ ][ ]<br><br>DON'T KNOW 98		
412	How many times did you receive antenatal care during this pregnancy?	NUMBER OF TIMES [ ][ ]<br><br>DON'T KNOW 98		
413	As part of your antenatal care during this pregnancy, were any of the following done at least once:<br><br>Was your blood pressure measured?<br>Did you give a urine sample?<br>Did you give a blood sample?	YES NO<br><br>BP . . . . 1 2<br>URINE . . . . . . 1 2<br>BLOOD 1 2		

NO.	QUESTIONS AND FILTERS	LAST BIRTH<br>NAME ____________	NEXT-TO-LAST BIRTH<br>NAME ____________	SECOND-FROM-LAST BIRTH<br>NAME ____________
414	During (any of) your antenatal care visit(s), were you told about things to look out for that might suggest problems with the pregnancy?	YES . . . . 1<br>NO . . . . 2<br>DON'T KNOW 8		
414A	What are the symptoms during pregnancy indicating the need to seek immediate care?<br><br>PROBE: Any other?<br><br>RECORD ALL MENTIONED	VAGINAL BLEEDING . . A<br>SEVERE LOWER<br>ABDOMEN PAIN . . . . B<br>SEVERE HEADACHE . . C<br>CONVULSION . . . . D<br>BLURRED VISION . . . . E<br>SWELLING FACE . . . . F<br>SWELLING HANDS<br>AND FEET . . . . G<br>OTHER ____________ X<br>(SPECIFY)<br>DON'T KNOW . . . . Z		
415	During this pregnancy, were you given an injection in the arm to prevent the baby from getting tetanus, that is, convulsions after birth?	YES . . . . 1<br>NO . . . . 2<br>(SKIP TO 418)<br>DON'T KNOW 8		
416	During this pregnancy, how many times did you get a tetanus injection?	TIMES . . . . [ ]<br>DON'T KNOW 8		
417	CHECK 416:	2 OR MORE TIMES [ ] (SKIP TO 421)<br>OTHER [ ]		
418	At any time before this pregnancy, did you receive any tetanus injections?	YES . . . . 1<br>NO . . . . 2<br>(SKIP TO 421)<br>DON'T KNOW 8		
419	Before this pregnancy, how many times did you receive a tetanus injection?<br><br>IF 7 OR MORE TIMES, RECORD '7'.	TIMES . . . . [ ]<br>DON'T KNOW 8		
420	How many years ago did you receive the last tetanus injection before this pregnancy?	YEARS AGO . . . . . . [ ][ ]		
421	During this pregnancy, were you given or did you buy any iron tablets or iron syrup?<br><br>SHOW TABLETS (TAQWAI KHON PILLS)	YES . . . . 1<br>NO . . . . 2<br>(SKIP TO 423)<br>DON'T KNOW 8		

NO.	QUESTIONS AND FILTERS	LAST BIRTH<br>NAME ________	NEXT-TO-LAST BIRTH<br>NAME ________	SECOND-FROM-LAST BIRTH<br>NAME ________
422	During the whole pregnancy, for how many days did you take the tablets or syrup?<br><br>IF ANSWER IS NOT NUMERIC, PROBE FOR APPROXIMATE NUMBER OF DAYS.	DAYS [ ][ ][ ]<br><br>DON'T KNOW 998		
423	During this pregnancy, did you take any drug for intestinal worms?	YES . . . . 1<br>NO . . . . 2<br>DON'T KNOW 8		
430	When (NAME) was born, was he/she very large, larger than average, average, smaller than average, or very small?	VERY LARGE . . . . 1<br>LARGER THAN AVERAGE . . . . 2<br>AVERAGE . . . . 3<br>SMALLER THAN AVERAGE . . . . 4<br>VERY SMALL . . . . 5<br>DON'T KNOW 8	VERY LARGE . . . . . 1<br>LARGER THAN AVERAGE . . . . . 2<br>AVERAGE . . . . 3<br>SMALLER THAN AVERAGE . . . . . 4<br>VERY SMALL . . . . . 5<br>DON'T KNOW 8	VERY LARGE . . . . . 1<br>LARGER THAN AVERAGE . . . . . 2<br>AVERAGE . . . . 3<br>SMALLER THAN AVERAGE . . . . . 4<br>VERY SMALL . . . . . 5<br>DON'T KNOW 8
431	Was (NAME) weighed at birth?	YES . . . . 1<br>NO . . . . 2<br>(SKIP TO 433)<br>DON'T KNOW 8	YES . . . . 1<br>NO . . . . 2<br>(SKIP TO 433)<br>DON'T KNOW 8	YES . . . . 1<br>NO . . . . 2<br>(SKIP TO 433)<br>DON'T KNOW 8
432	How much did (NAME) weigh?<br><br>RECORD WEIGHT IN KILOGRAMS FROM HEALTH CARD, IF AVAILABLE.	KG FROM CARD<br>1 [ ] . [ ][ ][ ]<br><br>KG FROM RECALL<br>2 [ ] . [ ][ ][ ]<br><br>DON'T KNOW 99998	KG FROM CARD<br>1 [ ] . [ ][ ][ ]<br><br>KG FROM RECALL<br>2 [ ] . [ ][ ][ ]<br><br>DON'T KNOW 99998	KG FROM CARD<br>1 [ ] . [ ][ ][ ]<br><br>KG FROM RECALL<br>2 [ ] . [ ][ ][ ]<br><br>DON'T KNOW 99998
433	Who assisted with the delivery of (NAME)?<br><br>Anyone else?<br><br>PROBE FOR THE TYPE(S) OF PERSON(S) AND RECORD ALL MENTIONED.<br><br>IF RESPONDENT SAYS NO ONE ASSISTED, PROBE TO DETERMINE WHETHER ANY ADULTS WERE PRESENT AT THE DELIVERY.	HEALTH PERSONNEL<br>DOCTOR . . . . A<br>NURSE/MIDWIFE . . B<br>AUXILIARY MIDWIFE . . . . C<br><br>OTHER PERSON<br>TRADITIONAL BIRTH ATTENDANT . . D<br>COM. HEALTH WK E<br>RELATIVE/FRIEND . F<br>OTHER ________ X<br>(SPECIFY)<br>NO ONE ASSISTED Y	HEALTH PERSONNEL<br>DOCTOR . . . . A<br>NURSE/MIDWIFE . B<br>AUXILIARY MIDWIFE . . . C<br><br>OTHER PERSON<br>TRADITIONAL BIRTH ATTENDANT . . D<br>COM. HEALTH WK E<br>RELATIVE/FRIEND . F<br>OTHER ________ X<br>(SPECIFY)<br>NO ONE ASSISTED Y	HEALTH PERSONNEL<br>DOCTOR . . . . . A<br>NURSE/MIDWIFE . B<br>AUXILIARY MIDWIFE . . . C<br><br>OTHER PERSON<br>TRADITIONAL BIRTH ATTENDANT . . D<br>COM. HEALTH WK E<br>RELATIVE/FRIEND F<br>OTHER ________ X<br>(SPECIFY)<br>NO ONE ASSISTED Y

NO.	QUESTIONS AND FILTERS	LAST BIRTH<br>NAME ________	NEXT-TO-LAST BIRTH<br>NAME ________	SECOND-FROM-LAST BIRTH<br>NAME ________
434	Where did you give birth to (NAME)?<br><br>PROBE TO IDENTIFY THE TYPE OF SOURCE.<br><br>IF UNABLE TO DETERMINE IF PUBLIC OR PRIVATE SECTOR, WRITE THE NAME OF THE PLACE.<br><br>________<br>(NAME OF PLACE)	HOME<br>YOUR HOME . . . . 11<br>(SKIP TO 438)<br>OTHER HOME . . . . 12<br><br>PUBLIC SECTOR<br>GOVT. HOSPITAL 21<br>CHC/POLY-CLINIC . . . . 22<br>BASIC HEALTH CENTER 23<br>HEALTH SUB-CENTEF 24<br>HP/SHP . . . . 25<br>MOBILE CLINIC . . 26<br>OTHER PUBLIC SECTOR ________ 27<br>(SPECIFY)<br><br>NGO<br>MARIE STOPES . 31<br>RED CROSS . . . . 32<br>OTHER NGO SECTOR ________ 36<br>(SPECIFY)<br><br>PRIVATE MED. SECTOR<br>PVT. HOSPITAL/ CLINIC . . . . 41<br>PVT. MATERNITY HOME . . . . 42<br>PVT. DOCTOR'S OFFICE . . . . 43<br>OTHER PRIVATE MED. SECTOR ________ 46<br>(SPECIFY)<br><br>OTHER SOURCE<br>CHARITY/ FOUNDATIONS 51<br>REFUGEE CAMP . 52<br><br>OTHER________ 96<br>(SPECIFY)<br>(SKIP TO 438)	HOME<br>YOUR HOME . . . 11<br>(SKIP TO 448)<br>OTHER HOME . . . 12<br><br>PUBLIC SECTOR<br>GOVT. HOSPITAL 21<br>CHC/POLY-CLINIC . . . . 22<br>BASIC HEALTH CENTER 23<br>HEALTH SUB-CENT 24<br>HP/SHP . . . . . 25<br>MOBILE CLINIC . 26<br>OTHER PUBLIC SECTOR ________ 27<br>(SPECIFY)<br><br>NGO<br>MARIE STOPES . 31<br>RED CROSS . . . 32<br>OTHER NGO SECTOR ________ 36<br>(SPECIFY)<br><br>PRIVATE MED. SECTOR<br>PVT. HOSPITAL/ CLINIC . . . . 41<br>PVT. MATERNITY HOME . . . . 42<br>PVT. DOCTOR'S OFFICE . . . . 43<br>OTHER PRIVATE MED. SECTOR ________ 46<br>(SPECIFY)<br><br>OTHER SOURCE<br>CHARITY/ FOUNDATIONS 51<br>REFUGEE CAMP . 52<br><br>OTHER________ 96<br>(SPECIFY)<br>(SKIP TO 448)	HOME<br>YOUR HOME . . . 11<br>(SKIP TO 448)<br>OTHER HOME . . . 12<br><br>PUBLIC SECTOR<br>GOVT. HOSPITAL 21<br>CHC/POLY-CLINIC . . . . 22<br>BASIC HEALTH CENTER 23<br>HEALTH SUB-CENT 24<br>HP/SHP . . . . . 25<br>MOBILE CLINIC . 26<br>OTHER PUBLIC SECTOR ________ 27<br>(SPECIFY)<br><br>NGO<br>MARIE STOPES . 31<br>RED CROSS . . . 32<br>OTHER NGO SECTOR ________ 36<br>(SPECIFY)<br><br>PRIVATE MED. SECTOR<br>PVT. HOSPITAL/ CLINIC . . . . 41<br>PVT. MATERNITY HOME . . . . 42<br>PVT. DOCTOR'S OFFICE . . . . 43<br>OTHER PRIVATE MED. SECTOR ________ 46<br>(SPECIFY)<br><br>OTHER SOURCE<br>CHARITY/ FOUNDATIONS 51<br>REFUGEE CAMP . 52<br><br>OTHER________ 96<br>(SPECIFY)<br>(SKIP TO 448)
434A	How long after (NAME) was delivered did you stay there?<br><br>IF LESS THAN ONE DAY, RECORD HOURS.<br>IF LESS THAN ONE WEEK, RECORD DAYS.	HOURS 1 [ ][ ]<br>DAYS 2 [ ][ ]<br>WEEKS 3 [ ][ ]<br><br>DON'T KNOW 998		
435	Was (NAME) delivered by caesarean, that is, did they cut your belly open to take the baby out?	YES . . . . 1<br>NO 2	YES . . . . 1<br>NO 2	YES . . . . 1<br>NO 2

NO.	QUESTIONS AND FILTERS	LAST BIRTH<br>NAME ____________	NEXT-TO-LAST BIRTH<br>NAME ____________	SECOND-FROM-LAST BIRTH<br>NAME ____________
436	I would like to talk to you about checks on your health after delivery, for example, someone asking you questions about your health or examining you. Did anyone check on your health while you were still in the facility?	YES . . . . 1<br>(SKIP TO 439)<br>NO 2		
437	Did anyone check on your health after you left the facility?	YES . . . . 1<br>(SKIP TO 439)<br>NO . . . . 2<br>(SKIP TO 442)		
438	I would like to talk to you about checks on your health after delivery, for example, someone asking you questions about your health or examining you. Did anyone check on your health after you gave birth to (NAME)?	YES . . . . 1<br>NO . . . . 2<br>(SKIP TO 442)		
439	Who checked on your health at that time?<br><br>PROBE FOR MOST QUALIFIED PERSON.	HEALTH PERSONNEL<br>DOCTOR . . . . 11<br>NURSE/MIDWIFE . . 12<br>AUXILIARY MIDWIFE . . . . 13<br><br>OTHER PERSON<br>TRADITIONAL BIRTH ATTENDANT 21<br>COMMUNITY HEALTH WORKER . . . . 22<br><br>OTHER ____________ 96<br>(SPECIFY)		
440	How long after delivery did the first check take place?<br><br>IF LESS THAN ONE DAY, RECORD HOURS.<br>IF LESS THAN ONE WEEK, RECORD DAYS.	HOURS 1 [ ][ ]<br>DAYS 2 [ ][ ]<br>WEEKS 3 [ ][ ]<br><br>DON'T KNOW 998		
440A	How many times did you receive postnatal care during this pregnancy?	NUMBER OF TIMES [ ][ ]<br><br>DON'T KNOW 98		
442	In the two months after (NAME) was born, did any health care provider or a traditional birth attendant check on his/her health?	YES . . . . 1<br>NO . . . . 2<br>(SKIP TO 446)<br>DON'T KNOW 8		
443	How many hours, days or weeks after the birth of (NAME) did the first check take place?<br><br>IF LESS THAN ONE DAY, RECORD HOURS.<br>IF LESS THAN ONE WEEK, RECORD DAYS.	HRS AFTER BIRTH . . 1 [ ][ ]<br>DAYS AFTER BIRTH . . 2 [ ][ ]<br>WKS AFTER BIRTH . . 3 [ ][ ]<br><br>DON'T KNOW 998		

NO.	QUESTIONS AND FILTERS	LAST BIRTH<br>NAME ________	NEXT-TO-LAST BIRTH<br>NAME ________	SECOND-FROM-LAST BIRTH<br>NAME ________
444	Who checked on (NAME)'s health at that time?<br><br>PROBE FOR MOST QUALIFIED PERSON.	HEALTH PERSONNEL<br>DOCTOR . . . . 11<br>NURSE/MIDWIFE . . 12<br>AUXILIARY MIDWIFE . . . . 13<br><br>OTHER PERSON<br>TRADITIONAL BIRTH ATTENDANT . . . . 21<br>COMMUNITY HEALTH WORKER . . . . 22<br><br>OTHER________ 96<br>(SPECIFY)		
445	Where did this first check of (NAME) take place?<br><br>PROBE TO IDENTIFY THE TYPE OF SOURCE AND CIRCLE THE APPROPRIATE CODE.<br><br>IF UNABLE TO DETERMINE IF PUBLIC OR PRIVATE SECTOR, WRITE THE<br><br>NAME OF THE PLACE.<br><br>________<br>(NAME OF PLACE)	HOME<br>YOUR HOME . . . . 11<br>OTHER HOME . . . . 12<br><br>PUBLIC SECTOR<br>GOVT. HOSPITAL . . 21<br>CHC/POLY-CLINIC . . . . 22<br>BASIC HEALTH CENTER . . . . 23<br>HEALTH SUB-CENTEF 24<br>HP/SHP . . . . 25<br>MOBILE CLINIC . . . . 26<br>OTHER PUBLIC<br>________ 27<br>(SPECIFY)<br><br>NGO<br>MARIE STOPES . . . . 31<br>RED CROSS . . . . 32<br>OTHER NGO SECTOR<br>________ 36<br>(SPECIFY)<br><br>PRIVATE MED. SECTOR<br>PVT. HOSPITAL/ CLINIC . . . . 41<br>PVT. MATERNITY HOME . . . . 42<br>PVT. DOCTOR'S OFFICI . . . . 43<br>OTHER PRIVATE MED.________ 46<br>(SPECIFY)<br><br>OTHER SOURCE<br>CHARITY/ FOUNDATIONS . . 51<br>REFUGEE CAMP . . 52<br><br>OTHER________ 96<br>(SPECIFY)		

NO.	QUESTIONS AND FILTERS	LAST BIRTH<br>NAME ________	NEXT-TO-LAST BIRTH<br>NAME ________	SECOND-FROM-LAST BIRTH<br>NAME ________
446	In the first two months after delivery, did you receive a vitamin A dose like (this/any of these)?<br><br>SHOW COMMON TYPES OF AMPULES/CAPSULES.	YES . . . . 1<br>NO . . . . 2<br>DON'T KNOW 8		
447	Has your menstrual period returned since the birth of (NAME)?	YES . . . . 1<br>(SKIP TO 449)<br>NO . . . . 2<br>(SKIP TO 450)		
448	Did your period return between the birth of (NAME) and your next pregnancy?		YES . . . . 1<br>NO . . . . 2<br>(SKIP TO 452)	YES . . . . 1<br>NO . . . . 2<br>(SKIP TO 452)
449	For how many months after the birth of (NAME) did you not have a period?	MONTHS . . . . [ ][ ]<br>DON'T KNOW 98	MONTHS . . . [ ][ ]<br>DON'T KNOW 98	MONTHS . . . [ ][ ]<br>DON'T KNOW 98
450	CHECK 226:<br><br>IS RESPONDENT PREGNANT?	NOT PREG-NANT [ ]<br>PREGNANT OR UNSURE [ ]<br>(SKIP TO 452)		
451	Have you had sexual intercourse since the birth of (NAME)?	YES . . . . 1<br>NO . . . . 2<br>(SKIP TO 453)		
452	For how many months after the birth of (NAME) did you not have sexual intercourse?	MONTHS . . . . [ ][ ]<br>DON'T KNOW 98	MONTHS . . . [ ][ ]<br>DON'T KNOW 98	MONTHS . . . [ ][ ]<br>DON'T KNOW 98
453	Did you ever breastfeed (NAME)?	YES . . . . 1<br>(SKIP TO 455)<br>NO 2	YES . . . . 1<br>NO 2	YES . . . . 1<br>NO 2
454	CHECK 404:<br><br>IS CHILD LIVING?	LIVING [ ]<br>(SKIP TO 460)<br>DEAD [ ]<br>(GO BACK TO 405 IN NEXT COLUMN; OR IF NO MORE BIRTHS, GO TO 501)		

NO.	QUESTIONS AND FILTERS	LAST BIRTH<br>NAME ______	NEXT-TO-LAST BIRTH<br>NAME ______	SECOND-FROM-LAST BIRTH<br>NAME ______
455	How long after birth did you first put (NAME) to the breast?<br><br>IF LESS THAN 1 HOUR, RECORD '00' HOURS.<br>IF LESS THAN 24 HOURS, RECORD HOURS.<br>OTHERWISE, RECORD DAYS.	IMMEDIATELY . . . . 000<br><br>HOURS 1 [ ][ ]<br>DAYS 2 [ ][ ]		
456	In the first three days after delivery, was (NAME) given anything to drink other than breast milk?	YES . . . . 1<br>NO . . . . 2<br>(SKIP TO 458)		
457	What was (NAME) given to drink?<br><br>Anything else?<br><br>RECORD ALL LIQUIDS MENTIONED.	MILK (OTHER THAN BREAST MILK) A<br>PLAIN WATER . . . . B<br>SUGAR OR GLUCOSE WATER . . . . C<br>GRIPE WATER . . . . D<br>SUGAR-SALT-WATER SOLUTION . . . . E<br>FRUIT JUICE . . . . F<br>INFANT FORMULA G<br>TEA/INFUSIONS . . . . H<br>COFFEE . . . . I<br>HONEY . . . . J<br><br>OTHER ______ X<br>(SPECIFY)		
458	CHECK 404:<br><br>IS CHILD LIVING?	LIVING [ ] DEAD [ ]<br>(GO BACK TO 405 IN NEXT COLUMN; OR, IF NO MORE BIRTHS, GO TO 501)	LIVING [ ] DEAD [ ]<br>(GO BACK TO 405 IN NEXT COLUMN; OR, IF NO MORE BIRTHS, GO TO 501)	LIVING [ ] DEAD [ ]<br>(GO BACK TO 405 IN NEXT-TO-LAST COLUMN OF NEW QUESTIONNAIRE; OR, IF NO MORE BIRTHS, GO TO 501)
459	Are you still breastfeeding (NAME)?	YES . . . . 1<br>NO 2		
460	Did (NAME) drink anything from a bottle with a nipple yesterday or last night?	YES . . . . 1<br>NO . . . . 2<br>DON'T KNOW 8	YES . . . . 1<br>NO . . . . 2<br>DON'T KNOW 8	YES . . . . 1<br>NO . . . . 2<br>DON'T KNOW 8
461		GO BACK TO 405 IN NEXT COLUMN; OR, IF NO MORE BIRTHS, GO TO 501.	GO BACK TO 405 IN NEXT COLUMN; OR, IF NO MORE BIRTHS, GO TO 501.	GO BACK TO 405 IN NEXT-TO-LAST COLUMN OF NEW QUESTIONNAIRE; OR, IF NO MORE BIRTHS, GO TO 501.

No.	Question			
501	ENTER IN THE TABLE THE BIRTH HISTORY NUMBER, NAME, AND SURVIVAL STATUS OF EACH BIRTH IN 1389 OR LATER.<br>ASK THE QUESTIONS ABOUT ALL OF THESE BIRTHS. BEGIN WITH THE LAST BIRTH.<br>(IF THERE ARE MORE THAN 3 BIRTHS, USE LAST 2 COLUMNS OF ADDITIONAL QUESTIONNAIRES).			
502	BIRTH HISTORY NUMBER FROM 212 IN BIRTH HISTORY	LAST BIRTH<br>BIRTH HISTORY NUMBER . . . . [ ][ ]	NEXT-TO-LAST BIRTH<br>BIRTH HISTORY NUMBER . . . . [ ][ ]	SECOND-FROM-LAST BIRTH<br>BIRTH HISTORY NUMBER . . . . . . [ ][ ]
503	FROM 212 AND 216	NAME ________<br>LIVING [ ]<br>DEAD [ ] (GO TO 503 IN NEXT COLUMN OR, IF NO MORE BIRTHS, GO TO 553)	NAME ________<br>LIVING [ ]<br>DEAD [ ] (GO TO 503 IN NEXT COLUMN OR, IF NO MORE BIRTHS, GO TO 553)	NAME ________<br>LIVING [ ]<br>DEAD [ ] (GO TO 503 IN NEXT-TO-LAST COLUMN OF NEW QUESTIONNAIRE, OR, IF NO MORE BIRTHS, GO TO 553)
504	Do you have a card where (NAME)'s vaccinations are written down?<br>IF YES:<br>May I see it please?	YES, SEEN . . . . 1<br>(SKIP TO 506)<br>YES, NOT SEEN . . . . 2<br>(SKIP TO 509)<br>NO CARD 3	YES, SEEN . . . . 1<br>(SKIP TO 506)<br>YES, NOT SEEN . . . . 2<br>(SKIP TO 509)<br>NO CARD 3	YES, SEEN . . . . 1<br>(SKIP TO 506)<br>YES, NOT SEEN . . . . . . 2<br>(SKIP TO 509)<br>NO CARD 3
505	Did you ever have a vaccination card for (NAME)?	YES . . . . 1<br>(SKIP TO 509)<br>NO 2	YES . . . . 1<br>(SKIP TO 509)<br>NO 2	YES . . . . 1<br>(SKIP TO 509)<br>NO 2

506

(1) COPY DATES FROM THE CARD.
(2) WRITE ‘44' IN ‘DAY' COLUMN IF CARD SHOWS THAT A DOSE WAS GIVEN, BUT NO DATE IS RECORDED.

	LAST BIRTH DAY	MONTH	YEAR		NEXT-TO-LAST BIRTH DAY	MONTH	YEAR		SECOND-FROM-LAST BIRTH DAY	MONTH	YEAR
BCG				BCG				BCG			
HEP B -0 (GIVEN AT BIRTH)				H0				H0			
POLIO 0 (GIVEN AT BIRTH)				P0				P0			
POLIO 1				P1				P1			
POLIO 2				P2				P2			
POLIO 3				P3				P3			
POLIO 4				P4				P4			
DPT 1/ PENTAVALENT 1				D1				D1			
DPT 2/ PENTAVALENT 2				D2				D2			
DPT 3/ PENTAVALENT 3				D3				D3			
PCV1				PC1				PC1			
PCV2				PC2				PC2			
PCV3				PC3				PC3			
MEASLES 1				M 1				M 1			
MEASLES 2				M 2				M 2			
VITAMIN A (MOST RECENT)				VIT A				VIT A			

No.	Question	LAST BIRTH	NEXT-TO-LAST BIRTH	SECOND-FROM-LAST BIRTH
507	CHECK 506:	BCG TO MEASLES 2 ALL RECORDED [ ] (GO TO 511)<br>OTHER [ ]	BCG TO MEASLES 2 ALL RECORDED [ ] (GO TO 511)<br>OTHER [ ]	BCG TO MEASLES 2 ALL RECORDED [ ] (GO TO 511)<br>OTHER [ ]

NO.	QUESTIONS AND FILTERS	LAST BIRTH<br>NAME ______	NEXT-TO-LAST BIRTH<br>NAME ______	SECOND-FROM-LAST BIRTH<br>NAME ______
508	Has (NAME) had any vaccinations that are not recorded on this card, including vaccinations given in a national immunization day campaign?<br><br>RECORD 'YES' ONLY IF THE RESPONDENT MENTIONS AT LEAST ONE OF THE VACCINATIONS IN 506 THAT ARE NOT RECORDED AS HAVING BEEN GIVEN.	YES . . . . 1<br>(PROBE FOR VACCINATIONS AND WRITE '66' IN THE CORRESPONDING DAY COLUMN IN 506)<br>(SKIP TO 511)<br>NO . . . . 2<br>(SKIP TO 511)<br>DON'T KNOW 8	YES . . . . 1<br>(PROBE FOR VACCINATIONS AND WRITE '66' IN THE CORRESPONDING DAY COLUMN IN 506)<br>(SKIP TO 511)<br>NO . . . . 2<br>(SKIP TO 511)<br>DON'T KNOW 8	YES . . . . 1<br>(PROBE FOR VACCINATIONS AND WRITE '66' IN THE CORRESPONDING DAY COLUMN IN 506)<br>(SKIP TO 511)<br>NO . . . . 2<br>(SKIP TO 511)<br>DON'T KNOW 8
509	Did (NAME) ever have any vaccinations to prevent him/her from getting diseases, including vaccinations received in a national immunization day campaign?	YES . . . . 1<br>NO . . . . 2<br>(SKIP TO 511)<br>DON'T KNOW 8	YES . . . . 1<br>NO . . . . 2<br>(SKIP TO 511)<br>DON'T KNOW 8	YES . . . . 1<br>NO . . . . 2<br>(SKIP TO 511)<br>DON'T KNOW 8
510	Please tell me if (NAME) had any of the following vaccinations:			
510A	A BCG vaccination against tuberculosis, that is, an injection in the arm or shoulder that usually causes a scar?	YES . . . . 1<br>NO . . . . 2<br>DON'T KNOW 8	YES . . . . 1<br>NO . . . . 2<br>DON'T KNOW 8	YES . . . . 1<br>NO . . . . 2<br>DON'T KNOW 8
510B	Hepatitis B-0 dose, that is given at birth, along with BCG?	YES . . . . 1<br>NO . . . . 2<br>DON'T KNOW 8	YES . . . . 1<br>NO . . . . 2<br>DON'T KNOW 8	YES . . . . 1<br>NO . . . . 2<br>DON'T KNOW 8
510C	Polio vaccine, that is, drops in the mouth?	YES . . . . 1<br>NO . . . . 2<br>(SKIP TO 510F)<br>DON'T KNOW 8	YES . . . . 1<br>NO . . . . 2<br>(SKIP TO 510F)<br>DON'T KNOW 8	YES . . . . 1<br>NO . . . . 2<br>(SKIP TO 510F)<br>DON'T KNOW 8
510D	Was the first polio vaccine given in the first two weeks after birth or later?	FIRST 2 WEEKS . . . 1<br>LATER 2	FIRST 2 WEEKS . . . 1<br>LATER 2	FIRST 2 WEEKS . . . 1<br>LATER 2
510E	How many times was the polio vaccine given?	NUMBER OF TIMES . . . . . ☐	NUMBER OF TIMES . . . . . ☐	NUMBER OF TIMES . . . . . ☐
510F	A DPT/PENTAVALENT vaccination, that is, an injection given in the thigh, sometimes at the same time as polio drops?	YES . . . . 1<br>NO . . . . 2<br>(SKIP TO 510H)<br>DON'T KNOW 8	YES . . . . 1<br>NO . . . . 2<br>(SKIP TO 510H)<br>DON'T KNOW 8	YES . . . . 1<br>NO . . . . 2<br>(SKIP TO 510H)<br>DON'T KNOW 8
510G	How many times was the DPT/PENTAVALENT vaccination	NUMBER OF TIMES . . . . . ☐	NUMBER OF TIMES . . . . . ☐	NUMBER OF TIMES . . . . . ☐
510H	A PCV vaccination, that is, an injection given in the thigh, to prevent him/her from getting pneumonia?	YES . . . . 1<br>NO . . . . 2<br>(SKIP TO 510J)<br>DON'T KNOW 8	YES . . . . 1<br>NO . . . . 2<br>(SKIP TO 510J)<br>DON'T KNOW 8	YES . . . . 1<br>NO . . . . 2<br>(SKIP TO 510J)<br>DON'T KNOW 8
510I	How many times was the PCV vaccination given?	NUMBER OF TIMES . . . . . ☐	NUMBER OF TIMES . . . . . ☐	NUMBER OF TIMES . . . . . ☐
510J	A measles injection or an MMR/MR injection- that is, a shot in the arm at the age of 9 months or older - to prevent him/her from getting measles?	YES . . . . 1<br>NO . . . . 2<br>(SKIP TO 511)<br>DON'T KNOW 8	YES . . . . 1<br>NO . . . . 2<br>(SKIP TO 511)<br>DON'T KNOW 8	YES . . . . 1<br>NO . . . . 2<br>(SKIP TO 511)<br>DON'T KNOW 8
510K	How many times was measles or MMR/MR injection given?	NUMBER OF TIMES . . . . . ☐	NUMBER OF TIMES . . . . . ☐	NUMBER OF TIMES . . . . . ☐

NO.	QUESTIONS AND FILTERS	LAST BIRTH<br>NAME ________	NEXT-TO-LAST BIRTH<br>NAME ________	SECOND-FROM-LAST BIRTH<br>NAME ________
511	Within the last six months, was (NAME) given a vitamin A dose like (this/any of these)?<br><br>SHOW COMMON TYPES OF CAPSULES.	YES . . . . 1<br>NO . . . . 2<br>DON'T KNOW 8	YES . . . . 1<br>NO . . . . 2<br>DON'T KNOW 8	YES . . . . 1<br>NO . . . . 2<br>DON'T KNOW 8
512	In the last seven days, was (NAME) given sprinkles with iron or any micronutrient powder like (this/any of these)?<br>SHOW COMMON TYPES OF SPRINKLES/SACHETS.	YES . . . . 1<br>NO . . . . 2<br>DON'T KNOW 8	YES . . . . 1<br>NO . . . . 2<br>DON'T KNOW 8	YES . . . . 1<br>NO . . . . 2<br>DON'T KNOW 8
513	Was (NAME) given any drug for intestinal worms in the last six months?	YES . . . . 1<br>NO . . . . 2<br>DON'T KNOW 8	YES . . . . 1<br>NO . . . . 2<br>DON'T KNOW 8	YES . . . . 1<br>NO . . . . 2<br>DON'T KNOW 8
514	Has (NAME) had diarrhea in the last 2 weeks?	YES . . . . 1<br>NO . . . . 2<br>(SKIP TO 525)←<br>DON'T KNOW 8	YES . . . . 1<br>NO . . . . 2<br>(SKIP TO 525)←<br>DON'T KNOW 8	YES . . . . 1<br>NO . . . . 2<br>(SKIP TO 525)←<br>DON'T KNOW 8
515	Was there any blood in the stools?	YES . . . . 1<br>NO . . . . 2<br>DON'T KNOW 8	YES . . . . 1<br>NO . . . . 2<br>DON'T KNOW 8	YES . . . . 1<br>NO . . . . 2<br>DON'T KNOW 8
516	Now I would like to know how much (NAME) was given to drink during the diarrhea (including breastmilk).<br><br>Was he/she given less than usual to drink, about the same amount, or more than usual to drink?<br><br>IF LESS, PROBE: Was he/she given much less than usual to drink or somewhat less?	MUCH LESS . . . . . 1<br>SOMEWHAT LESS 2<br>ABOUT THE SAME 3<br>MORE . . . . 4<br>NOTHING TO DRINK 5<br>DON'T KNOW 8	MUCH LESS . . . . . 1<br>SOMEWHAT LESS 2<br>ABOUT THE SAME 3<br>MORE . . . . 4<br>NOTHING TO DRINK 5<br>DON'T KNOW 8	MUCH LESS . . . . . 1<br>SOMEWHAT LESS 2<br>ABOUT THE SAME 3<br>MORE . . . . 4<br>NOTHING TO DRINK 5<br>DON'T KNOW 8
517	When (NAME) had diarrhea, was he/she given less than usual to eat, about the same amount, more than usual, or nothing to eat?<br><br>IF LESS, PROBE: Was he/she given much less than usual to eat or somewhat less?	MUCH LESS . . . . . 1<br>SOMEWHAT LESS 2<br>ABOUT THE SAME 3<br>MORE . . . . 4<br>STOPPED FOOD 5<br>NEVER GAVE FOOD 6<br>DON'T KNOW 8	MUCH LESS . . . . . 1<br>SOMEWHAT LESS 2<br>ABOUT THE SAME 3<br>MORE . . . . 4<br>STOPPED FOOD 5<br>NEVER GAVE FOOD 6<br>DON'T KNOW 8	MUCH LESS . . . . . 1<br>SOMEWHAT LESS 2<br>ABOUT THE SAME 3<br>MORE . . . . 4<br>STOPPED FOOD 5<br>NEVER GAVE FOOD 6<br>DON'T KNOW 8
518	Did you seek advice or treatment for the diarrhea from any source?	YES . . . . 1<br>NO . . . . 2<br>(SKIP TO 522)←	YES . . . . 1<br>NO . . . . 2<br>(SKIP TO 522)←	YES . . . . 1<br>NO . . . . 2<br>(SKIP TO 522)←

NO.	QUESTIONS AND FILTERS	LAST BIRTH<br>NAME ________	NEXT-TO-LAST BIRTH<br>NAME ________	SECOND-FROM-LAST BIRTH<br>NAME ________
519	Where did you seek advice or treatment?<br><br>Anywhere else?<br><br>PROBE TO IDENTIFY EACH TYPE OF SOURCE.<br><br>IF UNABLE TO DETERMINE IF PUBLIC OR PRIVATE SECTOR, WRITE THE NAME OF THE PLACE.<br><br>________<br>(NAME OF PLACE(S))	PUBLIC SECTOR<br>GOVT HOSPITAL . A<br>CHC/POLY-<br>CLINIC . . . . B<br>BASIC HEALTH<br>CENTER . . . . . C<br>HSC . . . . D<br>HP/SHP . . . . E<br>COMM. HEALTH<br>WORKER . . . . . F<br>MOBILE CLINIC . G<br>OTHER PUBLIC<br>SECTOR<br>________ H<br>(SPECIFY)<br><br>NON-GOVERNMENT<br>MARIE STOPES . I<br>RED CROSS . . . J<br>OTHER NGO<br>SECTOR<br>________ K<br>(SPECIFY)<br><br>PRIVATE MEDICAL<br>SECTOR<br>PVT. HOSPITAL/<br>CLINIC . . . . L<br>PHARMACY . . . M<br>PVT DOCTOR'S<br>OFFICE . . . . . N<br>OTHER PRIVATE<br>MED. SECTOR<br>________ O<br>(SPECIFY)<br><br>OTHER SOURCE<br>CHARITY/<br>FOUNDATIONS P<br>REFUGEE CAMP . Q<br>SHOP . . . . R<br>TRADITIONAL<br>PRACTITIONER S<br>MARKET . . . . . T<br><br>OTHER ________ X<br>(SPECIFY)	PUBLIC SECTOR<br>GOVT HOSPITAL . A<br>CHC/POLY-<br>CLINIC . . . . B<br>BASIC HEALTH<br>CENTER . . . . . C<br>HSC . . . . D<br>HP/SHP . . . . E<br>COMM. HEALTH<br>WORKER . . . . . F<br>MOBILE CLINIC . G<br>OTHER PUBLIC<br>SECTOR<br>________ H<br>(SPECIFY)<br><br>NON-GOVERNMENT<br>MARIE STOPES . I<br>RED CROSS . . . J<br>OTHER NGO<br>SECTOR<br>________ K<br>(SPECIFY)<br><br>PRIVATE MEDICAL<br>SECTOR<br>PVT. HOSPITAL/<br>CLINIC . . . . L<br>PHARMACY . . . M<br>PVT DOCTOR'S<br>OFFICE . . . . . N<br>OTHER PRIVATE<br>MED. SECTOR<br>________ O<br>(SPECIFY)<br><br>OTHER SOURCE<br>CHARITY/<br>FOUNDATIONS P<br>REFUGEE CAMP . Q<br>SHOP . . . . R<br>TRADITIONAL<br>PRACTITIONER S<br>MARKET . . . . . T<br><br>OTHER ________ X<br>(SPECIFY)	PUBLIC SECTOR<br>GOVT HOSPITAL . A<br>CHC/POLY-<br>CLINIC . . . . B<br>BASIC HEALTH<br>CENTER . . . . . C<br>HSC . . . . D<br>HP/SHP . . . . E<br>COMM. HEALTH<br>WORKER . . . . . F<br>MOBILE CLINIC . G<br>OTHER PUBLIC<br>SECTOR<br>________ H<br>(SPECIFY)<br><br>NON-GOVERNMENT<br>MARIE STOPES . I<br>RED CROSS . . . J<br>OTHER NGO<br>SECTOR<br>________ K<br>(SPECIFY)<br><br>PRIVATE MEDICAL<br>SECTOR<br>PVT. HOSPITAL/<br>CLINIC . . . . L<br>PHARMACY . . . M<br>PVT DOCTOR'S<br>OFFICE . . . . . N<br>OTHER PRIVATE<br>MED. SECTOR<br>________ O<br>(SPECIFY)<br><br>OTHER SOURCE<br>CHARITY/<br>FOUNDATIONS P<br>REFUGEE CAMP . Q<br>SHOP . . . . R<br>TRADITIONAL<br>PRACTITIONER S<br>MARKET . . . . . T<br><br>OTHER ________ X<br>(SPECIFY)
520	CHECK 519:	TWO OR MORE CODES CIRCLED ☐<br>ONLY ONE CODE CIRCLED ☐ (SKIP TO 522)	TWO OR MORE CODES CIRCLED ☐<br>ONLY ONE CODE CIRCLED ☐ (SKIP TO 522)	TWO OR MORE CODES CIRCLED ☐<br>ONLY ONE CODE CIRCLED ☐ (SKIP TO 522)
521	Where did you first seek advice or treatment?<br><br>USE LETTER CODE FROM 519.	FIRST PLACE . . . ☐	FIRST PLACE . . . ☐	FIRST PLACE . . . ☐

NO.	QUESTIONS AND FILTERS	LAST BIRTH<br>NAME ________	NEXT-TO-LAST BIRTH<br>NAME ________	SECOND-FROM-LAST BIRTH<br>NAME ________
522	Was he/she given any of the following to drink at any time since he/she started having the diarrhea:	YES NO DK	YES NO DK	YES NO DK
	a) A fluid made from a special packet called SHEFA?	FLUID FROM ORS PKT 1 2 8	FLUID FROM ORS PKT 1 2 8	FLUID FROM ORS PKT 1 2 8
	b) A pre-packaged ORS liquid?	ORS LQD 1 2 8	ORS LQD 1 2 8	ORS LQD 1 2 8
	c) A government-recommended homemade fluid? (*Wheat Salt Solution WSS*)	HOMEMADE WSS . . . 1 2 8	HOMEMADE WSS . . . 1 2 8	HOMEMADE WSS . . . 1 2 8
	d) A government-recommended homemade fluid? (*Salt & Sugar Solution SSS*)	HOMEMADE SSS . . . 1 2 8	HOMEMADE SSS . . . 1 2 8	HOMEMADE SSS . . . 1 2 8
523	Was anything (else) given to treat the diarrhea?	YES . . . . 1<br>NO . . . . 2<br>(SKIP TO 525)←┤<br>DON'T KNOW 8	YES . . . . 1<br>NO . . . . 2<br>(SKIP TO 525)←┤<br>DON'T KNOW 8	YES . . . . 1<br>NO . . . . 2<br>(SKIP TO 525)←┤<br>DON'T KNOW 8
524	What (else) was given to treat the diarrhea?<br><br>Anything else?<br><br>RECORD ALL TREATMENTS GIVEN.	PILL OR SYRUP<br>ANTIBIOTIC . . . . . A<br>ANTIMOTILITY B<br>ZINC . . . . C<br>OTHER (NOT ANTI-BIOTIC, ANTI-MOTILITY, OR ZINC) . . . . D<br>UNKNOWN PILL OR SYRUP . . . E<br><br>INJECTION<br>ANTIBIOTIC . . . . . F<br>NON-ANTIBIOTIC G<br>UNKNOWN INJECTION . . . H<br><br>(IV) INTRAVENOUS I<br><br>HOME REMEDY/ HERBAL MED-ICINE . . . . J<br><br>OTHER ________ X<br>(SPECIFY)	PILL OR SYRUP<br>ANTIBIOTIC . . . . . A<br>ANTIMOTILITY B<br>ZINC . . . . C<br>OTHER (NOT ANTI-BIOTIC, ANTI-MOTILITY, OR ZINC) . . . . D<br>UNKNOWN PILL OR SYRUP . . . E<br><br>INJECTION<br>ANTIBIOTIC . . . . . F<br>NON-ANTIBIOTIC G<br>UNKNOWN INJECTION . . . H<br><br>(IV) INTRAVENOUS I<br><br>HOME REMEDY/ HERBAL MED-ICINE . . . . J<br><br>OTHER ________ X<br>(SPECIFY)	PILL OR SYRUP<br>ANTIBIOTIC . . . . . A<br>ANTIMOTILITY B<br>ZINC . . . . C<br>OTHER (NOT ANTI-BIOTIC, ANTI-MOTILITY, OR ZINC) . . . . D<br>UNKNOWN PILL OR SYRUP . . . E<br><br>INJECTION<br>ANTIBIOTIC . . . . . F<br>NON-ANTIBIOTIC G<br>UNKNOWN INJECTION . . . H<br><br>(IV) INTRAVENOUS I<br><br>HOME REMEDY/ HERBAL MED-ICINE . . . . J<br><br>OTHER ________ X<br>(SPECIFY)
525	Has (NAME) been ill with a fever at any time in the last 2 weeks?	YES . . . . 1<br>NO . . . . 2<br>(SKIP TO 527)←┤<br>DON'T KNOW 8	YES . . . . 1<br>NO . . . . 2<br>(SKIP TO 527)←┤<br>DON'T KNOW 8	YES . . . . 1<br>NO . . . . 2<br>(SKIP TO 527)←┤<br>DON'T KNOW 8
526	At any time during the illness, did (NAME) have blood taken from his/her finger or heel for testing?	YES . . . . 1<br>NO . . . . 2<br>DON'T KNOW 8	YES . . . . 1<br>NO . . . . 2<br>DON'T KNOW 8	YES . . . . 1<br>NO . . . . 2<br>DON'T KNOW 8
527	Has (NAME) had an illness with a cough at any time in the last 2 weeks?	YES . . . . 1<br>NO . . . . 2<br>(SKIP TO 530)←┤<br>DON'T KNOW 8	YES . . . . 1<br>NO . . . . 2<br>(SKIP TO 530)←┤<br>DON'T KNOW 8	YES . . . . 1<br>NO . . . . 2<br>(SKIP TO 530)←┤<br>DON'T KNOW 8

NO.	QUESTIONS AND FILTERS	LAST BIRTH<br>NAME ______	NEXT-TO-LAST BIRTH<br>NAME ______	SECOND-FROM-LAST BIRTH<br>NAME ______
528	When (NAME) had an illness with a cough, did he/she breathe faster than usual with short, rapid breaths or have difficulty breathing?	YES . . . . 1<br>NO . . . . 2<br>(SKIP TO 531)<br>DON'T KNOW 8	YES . . . . 1<br>NO . . . . 2<br>(SKIP TO 531)<br>DON'T KNOW 8	YES . . . . 1<br>NO . . . . 2<br>(SKIP TO 531)<br>DON'T KNOW 8
529	Was the fast or difficult breathing due to a problem in the chest or to a blocked or runny nose?	CHEST ONLY . . . 1<br>NOSE ONLY . . . . . 2<br>BOTH . . . . 3<br>OTHER ______ 6<br>(SPECIFY)<br>DON'T KNOW . . . . . 8<br>(SKIP TO 531)	CHEST ONLY . . . 1<br>NOSE ONLY . . . . . 2<br>BOTH . . . . 3<br>OTHER ______ 6<br>(SPECIFY)<br>DON'T KNOW . . . . . 8<br>(SKIP TO 531)	CHEST ONLY . . . 1<br>NOSE ONLY . . . . . 2<br>BOTH . . . . 3<br>OTHER ______ 6<br>(SPECIFY)<br>DON'T KNOW . . . . . 8<br>(SKIP TO 531)
530	CHECK 525:<br><br>HAD FEVER?	YES ☐ NO OR DK ☐<br>(GO BACK TO 503 IN NEXT COLUMN; OR, IF NO MORE BIRTHS, GO TO 553)	YES ☐ NO OR DK ☐<br>(GO BACK TO 503 IN NEXT COLUMN; OR, IF NO MORE BIRTHS, GO TO 553)	YES ☐ NO OR DK ☐<br>(GO TO 503 IN NEXT-TO-LAST COLUMN OF NEW QUESTIONNAIRE; OR, IF NO MORE BIRTHS, GO TO 553)
531	Now I would like to know how much (NAME) was given to drink (including breastmilk) during the illness with a (fever/cough).<br>Was he/she given less than usual to drink, about the same amount, or more than usual to drink?<br><br>IF LESS, PROBE: Was he/she given much less than usual to drink or somewhat less?	MUCH LESS . . . . . 1<br>SOMEWHAT LESS 2<br>ABOUT THE SAME 3<br>MORE . . . . 4<br>NOTHING TO DRINK 5<br>DON'T KNOW 8	MUCH LESS . . . . . 1<br>SOMEWHAT LESS 2<br>ABOUT THE SAME 3<br>MORE . . . . 4<br>NOTHING TO DRINK 5<br>DON'T KNOW 8	MUCH LESS . . . . . 1<br>SOMEWHAT LESS 2<br>ABOUT THE SAME 3<br>MORE . . . . 4<br>NOTHING TO DRINK 5<br>DON'T KNOW 8
532	When (NAME) had a (fever/cough), was he/she given less than usual to eat, about the same amount, more than usual, or nothing to eat?<br><br>IF LESS, PROBE: Was he/she given much less than usual to eat or somewhat less?	MUCH LESS . . . . . 1<br>SOMEWHAT LESS 2<br>ABOUT THE SAME 3<br>MORE . . . . 4<br>STOPPED FOOD 5<br>NEVER GAVE FOOD 6<br>DON'T KNOW 8	MUCH LESS . . . . . 1<br>SOMEWHAT LESS 2<br>ABOUT THE SAME 3<br>MORE . . . . 4<br>STOPPED FOOD 5<br>NEVER GAVE FOOD 6<br>DON'T KNOW 8	MUCH LESS . . . . . 1<br>SOMEWHAT LESS 2<br>ABOUT THE SAME 3<br>MORE . . . . 4<br>STOPPED FOOD 5<br>NEVER GAVE FOOD 6<br>DON'T KNOW 8
533	Did you seek advice or treatment for the illness from any source?	YES . . . . 1<br>NO . . . . 2<br>(SKIP TO 537)	YES . . . . 1<br>NO . . . . 2<br>(SKIP TO 537)	YES . . . . 1<br>NO . . . . 2<br>(SKIP TO 537)

NO.	QUESTIONS AND FILTERS	LAST BIRTH<br>NAME ______________	NEXT-TO-LAST BIRTH<br>NAME ______________	SECOND-FROM-LAST BIRTH<br>NAME ______________
534	Where did you seek advice or treatment?<br><br>Anywhere else?<br><br>PROBE TO IDENTIFY EACH TYPE OF SOURCE.<br><br>IF UNABLE TO DETERMINE IF PUBLIC OR PRIVATE SECTOR, WRITE THE NAME OF THE PLACE.<br><br>______________<br>(NAME OF PLACE(S))	PUBLIC SECTOR<br>GOVT HOSPITAL A<br>CHC/POLY-<br>CLINIC . . . . B<br>BASIC HEALTH<br>CENTER . . . . . C<br>HSC . . . . D<br>HP/SHP . . . . E<br>COMM. HEALTH<br>WORKER . . . . . F<br>MOBILE CLINIC . G<br>OTHER PUBLIC<br>SECTOR<br>____________ H<br>(SPECIFY)<br><br>NON-GOVERNMENT<br>MARIE STOPES . I<br>RED CROSS . . . J<br>OTHER NGO<br>SECTOR<br>____________ K<br>(SPECIFY)<br><br>PRIVATE MEDICAL<br>SECTOR<br>PVT HOSPITAL/<br>CLINIC . . . . L<br>PHARMACY . . . M<br>PVT DOCTOR . . . N<br>OTHER PRIVATE<br>MED. SECTOR<br>____________ O<br>(SPECIFY)<br><br>OTHER SOURCE<br>CHARITY/<br>FOUNDATIONS P<br>REFUGEE CAMP . Q<br>SHOP . . . . R<br>TRADITIONAL<br>PRACTITIONER S<br>MARKET . . . . . T<br><br>OTHER ________ X<br>(SPECIFY)	PUBLIC SECTOR<br>GOVT HOSPITAL A<br>CHC/POLY-<br>CLINIC . . . . B<br>BASIC HEALTH<br>CENTER . . . . . C<br>HSC . . . . D<br>HP/SHP . . . . E<br>COMM. HEALTH<br>WORKER . . . . . F<br>MOBILE CLINIC . G<br>OTHER PUBLIC<br>SECTOR<br>____________ H<br>(SPECIFY)<br><br>NON-GOVERNMENT<br>MARIE STOPES . I<br>RED CROSS . . . J<br>OTHER NGO<br>SECTOR<br>____________ K<br>(SPECIFY)<br><br>PRIVATE MEDICAL<br>SECTOR<br>PVT HOSPITAL/<br>CLINIC . . . . L<br>PHARMACY . . . M<br>PVT DOCTOR . . . N<br>OTHER PRIVATE<br>MED. SECTOR<br>____________ O<br>(SPECIFY)<br><br>OTHER SOURCE<br>CHARITY/<br>FOUNDATIONS P<br>REFUGEE CAMP . Q<br>SHOP . . . . R<br>TRADITIONAL<br>PRACTITIONER S<br>MARKET . . . . . T<br><br>OTHER ________ X<br>(SPECIFY)	PUBLIC SECTOR<br>GOVT HOSPITAL A<br>CHC/POLY-<br>CLINIC . . . . B<br>BASIC HEALTH<br>CENTER . . . . . C<br>HSC . . . . D<br>HP/SHP . . . . E<br>COMM. HEALTH<br>WORKER . . . . . F<br>MOBILE CLINIC . G<br>OTHER PUBLIC<br>SECTOR<br>____________ H<br>(SPECIFY)<br><br>NON-GOVERNMENT<br>MARIE STOPES . I<br>RED CROSS . . . J<br>OTHER NGO<br>SECTOR<br>____________ K<br>(SPECIFY)<br><br>PRIVATE MEDICAL<br>SECTOR<br>PVT HOSPITAL/<br>CLINIC . . . . L<br>PHARMACY . . . M<br>PVT DOCTOR . . . N<br>OTHER PRIVATE<br>MED. SECTOR<br>____________ O<br>(SPECIFY)<br><br>OTHER SOURCE<br>CHARITY/<br>FOUNDATIONS P<br>REFUGEE CAMP . Q<br>SHOP . . . . R<br>TRADITIONAL<br>PRACTITIONER S<br>MARKET . . . . . T<br><br>OTHER ________ X<br>(SPECIFY)
535	CHECK 534:	TWO OR MORE CODES CIRCLED ☐<br>ONLY ONE CODE CIRCLED ☐ (SKIP TO 537)	TWO OR MORE CODES CIRCLED ☐<br>ONLY ONE CODE CIRCLED ☐ (SKIP TO 537)	TWO OR MORE CODES CIRCLED ☐<br>ONLY ONE CODE CIRCLED ☐ (SKIP TO 537)
536	Where did you first seek advice or treatment?<br><br>USE LETTER CODE FROM 534.	FIRST PLACE . . . ☐	FIRST PLACE . . . ☐	FIRST PLACE . . . ☐

NO.	QUESTIONS AND FILTERS	LAST BIRTH<br>NAME _______	NEXT-TO-LAST BIRTH<br>NAME _______	SECOND-FROM-LAST BIRTH<br>NAME _______
537	At any time during the illness, did (NAME) take any drugs for the illness?	YES . . . . 1<br>NO . . . . 2<br>(GO BACK TO 503 IN NEXT COLUMN; OR, IF NO MORE BIRTHS, GO TO 553)<br>DON'T KNOW 8	YES . . . . 1<br>NO . . . . 2<br>(GO BACK TO 503 IN NEXT COLUMN; OR, IF NO MORE BIRTHS, GO TO 553)<br>DON'T KNOW 8	YES . . . . 1<br>NO . . . . 2<br>(GO TO 503 IN NEXT-TO-LAST COLUMN OF NEW QUESTIONNAIRE; OR, IF NO MORE BIRTHS, GO TO 553)<br>DON'T KNOW 8
538	What drugs did (NAME) take?<br><br>Any other drugs?<br><br>RECORD ALL MENTIONED.	ANTIMALARIAL DRUGS<br>SP/FANSIDAR . . . A<br>CHLOROQUINE B<br>AMODIAQUINE C<br>QUININE . . . . D<br>COMBINATION WITH ARTEMISININ E<br>ARTESUNATE MONOTHERAPY F<br>OTHER ANTI-MALARIAL ________ . . . G<br>(SPECIFY)<br><br>ANTIBIOTIC DRUGS<br>PILL/SYRUP . . . H<br>INJECTION . . . I<br><br>OTHER DRUGS<br>ASPRIN . . . . J<br>PARA-CETAMOL . . . K<br>IBUPROFEN . . . L<br><br>OTHER ________ X<br>(SPECIFY)<br>DON'T KNOW . . . . . Z	ANTIMALARIAL DRUGS<br>SP/FANSIDAR . . . A<br>CHLOROQUINE . B<br>AMODIAQUINE C<br>QUININE . . . . D<br>COMBINATION WITH ARTEMISININ E<br>ARTESUNATE MONOTHERAPY F<br>OTHER ANTI-MALARIAL ________ . . . G<br>(SPECIFY)<br><br>ANTIBIOTIC DRUGS<br>PILL/SYRUP . . . H<br>INJECTION . . . I<br><br>OTHER DRUGS<br>ASPRIN . . . . J<br>PARA-CETAMOL . . . K<br>IBUPROFEN . . . L<br><br>OTHER ________ X<br>(SPECIFY)<br>DON'T KNOW . . . . . Z	ANTIMALARIAL DRUGS<br>SP/FANSIDAR . . . A<br>CHLOROQUINE B<br>AMODIAQUINE C<br>QUININE . . . . D<br>COMBINATION WITH ARTEMISININ E<br>ARTESUNATE MONOTHERAPY F<br>OTHER ANTI-MALARIAL ________ . . . G<br>(SPECIFY)<br><br>ANTIBIOTIC DRUGS<br>PILL/SYRUP . . . H<br>INJECTION . . . I<br><br>OTHER DRUGS<br>ASPRIN . . . . J<br>PARA-CETAMOL . . . K<br>IBUPROFEN . . . L<br><br>OTHER ________ X<br>(SPECIFY)<br>DON'T KNOW . . . . . Z
552		GO BACK TO 503 IN NEXT COLUMN; OR, IF NO MORE BIRTHS, GO TO 553.	GO BACK TO 503 IN NEXT COLUMN; OR, IF NO MORE BIRTHS, GO TO 553.	GO TO 503 IN NEXT-TO-LAST COLUMN OF NEW QUESTIONNAIRE; OR, IF NO MORE BIRTHS, GO TO 553.

NO.	QUESTIONS AND FILTERS	CODING CATEGORIES	SKIP
553	CHECK 215 AND 218, ALL ROWS:<br><br>NUMBER OF CHILDREN BORN IN 1389 OR LATER LIVING WITH THE RESPONDENT<br><br>ONE OR MORE ☐ ↓ NONE ☐ →<br><br>RECORD NAME OF YOUNGEST CHILD LIVING WITH HER AND CONTINUE WITH 554<br><br>______________________<br>(NAME)		556
554	The last time (NAME FROM 553) passed stools, what was done to dispose of the stools?	CHILD USED TOILET OR LATRINE . . . 01<br>PUT/RINSED<br>INTO TOILET OR LATRINE . . . . 02<br>PUT/RINSED<br>INTO DRAIN OR DITCH . . . . 03<br>THROWN INTO GARBAGE . . . 04<br>BURIED . . . . 05<br>LEFT IN THE OPEN . . . . 06<br>OTHER ______________ 96<br>(SPECIFY)	
555	CHECK 522(a) AND 522 (b), ALL COLUMNS:<br><br>NO CHILD RECEIVED FLUID FROM ORS PACKET OR PRE-PACKAGED ORS LIQUID ☐ ↓<br><br>ANY CHILD RECEIVED FLUID FROM ORS PACKET OR PRE-PACKAGED ORS LIQUID ☐ →		556A
556	Have you ever heard of a special product called ORS (e.g. SHEFA) you can get for the treatment of diarrhea?	YES . . . . 1<br>NO 2	
556A	Sometimes children have severe illness and should be taken immediately to a health facility. What types of symptoms would cause you to take your child to a health facility right away?<br><br>Any other symptoms?	CHILD NOT ABLE TO DRINK OR<br>BREASTFEED . . . . A<br>CHILD BECOMES SICKER . . . . B<br>CHILD DEVELOPS A FEVER . . . . C<br>CHILD HAS FAST BREATHING . . . . . D<br>CHILD HAS DIFFICULT BREATHING . E<br>CHILD HAS BLOOD IN STOOL . . . . . F<br>CHILD IS DRINKING POORLY . . . . G<br><br>OTHER ______________ x<br>(SPECIFY)	
557	CHECK 215 AND 218, ALL ROWS:<br><br>NUMBER OF CHILDREN BORN IN 1392 OR LATER LIVING WITH THE RESPONDENT<br><br>ONE OR MORE ☐ ↓ NONE ☐ →<br><br>RECORD NAME OF YOUNGEST CHILD LIVING WITH HER AND CONTINUE WITH 558<br><br>______________________<br>(NAME)		601

NO.	QUESTIONS AND FILTERS	CODING CATEGORIES	SKIP
558	Now I would like to ask you about liquids or foods that (NAME FROM 557) had yesterday during the day or at night. I am interested in whether your child had the item I mention even if it was combined with other foods.<br><br>Did (NAME FROM 557) (drink/eat):	YES NO DK	
	a) Plain water?	**a)** 1 2 8	
	b) Juice or juice drinks?	**b)** 1 2 8	
	c) Clear broth?	**c)** 1 2 8	
	d) Milk such as tinned, powdered, or fresh animal milk?<br>IF YES: How many times did (NAME) drink milk?<br>IF 7 OR MORE TIMES, RECORD '7'.	**d)** 1 2 8<br>NUMBER OF TIMES DRANK MILK ☐	
	e) Infant formula?<br>IF YES: How many times did (NAME) drink infant formula?<br>IF 7 OR MORE TIMES, RECORD '7'.	**e)** 1 2 8<br>NUMBER OF TIMES DRANK FORMULA ☐	
	f) Any other liquids?	**f)** 1 2 8	
	g) Yogurt?<br>IF YES: How many times did (NAME) eat yogurt?<br>IF 7 OR MORE TIMES, RECORD '7'.	**g)** 1 2 8<br>NUMBER OF TIMES ATE YOGURT ☐	
	h) Any [BRAND NAME OF COMMERCIALLY FORTIFIED BABY FOOD, E.G., Cerelac]?	**h)** 1 2 8	
	i) Bread, rice, noodles, porridge, or other foods made from grains?	**i)** 1 2 8	
	j) Pumpkin, carrots, squash that are yellow or orange inside?	**j)** 1 2 8	
	k) White potatoes, manioc, cassava, or any other foods made from roots?	**k)** 1 2 8	
	l) Any dark green, leafy vegetables?	**l)** 1 2 8	
	m) Ripe mangoes or other vitamin-A rich fruits?	**m)** 1 2 8	
	n) Any other fruits or vegetables?	**n)** 1 2 8	
	o) Liver, kidney, heart or other organ meats?	**o)** 1 2 8	
	p) Any meat, such as beef, lamb, goat, chicken, or duck?	**p)** 1 2 8	
	q) Eggs?	**q)** 1 2 8	
	r) Fresh or dried fish?	**r)** 1 2 8	
	s) Any foods made from beans, peas, lentils, or nuts?	**s)** 1 2 8	
	t) Cheese or other food made from milk?	**t)** 1 2 8	
	u) Any other solid, semi-solid, or soft food?	**u)** 1 2 8	
559	CHECK 558 (CATEGORIES "g" THROUGH "u"):<br>NOT A SINGLE "YES" ☐ ↓	AT LEAST ONE "YES" ☐ →	561

NO.	QUESTIONS AND FILTERS	CODING CATEGORIES	SKIP
560	Did (NAME) eat any solid, semi-solid, or soft foods yesterday during the day or at night?<br><br>IF 'YES' PROBE: What kind of solid, semi-solid or soft foods did (NAME) eat?	YES . . . . 1<br>(GO BACK TO 558 TO RECORD FOOD EATEN YESTERDAY)<br><br>NO 2	 <br><br>→ 601
561	How many times did (NAME FROM 557) eat solid, semi-solid, or soft foods yesterday during the day or at night?<br><br>IF 7 OR MORE TIMES, RECORD '7'.	NUMBER OF TIMES . . . . [ ]<br><br>DON'T KNOW 8	

SECTION 6. MARRIAGE AND SEXUAL ACTIVITY

NO.	QUESTIONS AND FILTERS	CODING CATEGORIES	SKIP
601	What is your current marital status: are you married, widowed, divorced, or separated?	CURRENTLY MARRIED .... 1<br>WIDOWED .... 2<br>DIVORCED .... 3<br>SEPARATED 4	 2, 3, 4 → 609
604	Is your husband living with you now or is he staying elsewhere?	LIVING WITH HER .... 1<br>STAYING ELSEWHERE 2	
605	RECORD THE HUSBAND'S NAME AND LINE NUMBER FROM THE HOUSEHOLD QUESTIONNAIRE.<br>IF HE IS NOT LISTED IN THE HOUSEHOLD, RECORD '00'.	NAME ______________________<br><br>LINE NO. .... [ ][ ]	
606	Does your husband have other wives or does he live with other women as if married?	YES .... 1<br>NO .... 2<br>DON'T KNOW 8	 2, 8 → 609
607	Including yourself, in total, how many wives does he have?	TOTAL NUMBER OF WIVES . [ ][ ]<br><br>DON'T KNOW 98	
608	Are you the first, second, … wife?	RANK .... [ ][ ]	
609	Have you been married only once or more than once?	ONLY ONCE .... 1<br>MORE THAN ONCE 2	
610	CHECK 609:<br><br>MARRIED ONLY ONCE [ ] ↓<br>In what month and year did you start living with your husband?<br><br>MARRIED MORE THAN ONCE [ ] ↓<br>Now I would like to ask about your first husband. In what month and year did you start living with him?	MONTH .... [ ][ ]<br><br>DON'T KNOW MONTH .... 98<br><br>YEAR .... [ ][ ][ ][ ]<br><br>DON'T KNOW YEAR 9998	 <br><br><br>→ 612
611	How old were you when you first started living with him?	AGE .... [ ][ ]	
612	CHECK FOR THE PRESENCE OF OTHERS. BEFORE CONTINUING, MAKE EVERY EFFORT TO ENSURE PRIVACY.		
613	Now I would like to ask some questions about sexual activity in order to gain a better understanding of some important life issues.<br><br>How old were you when you had sexual intercourse for the very first time?	NEVER HAD SEXUAL INTERCOURSE .... 00<br><br>AGE IN YEARS .... [ ][ ]<br><br>FIRST TIME WHEN STARTED LIVING WITH (FIRST) HUSBAND 95	→ 628

NO.	QUESTIONS AND FILTERS	CODING CATEGORIES	SKIP
614	Now I would like to ask you some questions about your recent sexual activity. Let me assure you again that your answers are completely confidential and will not be told to anyone. If we should come to any question that you don't want to answer, just let me know and we will go to the next question.		
615	When was the last time you had sexual intercourse?<br><br>IF LESS THAN 12 MONTHS, ANSWER MUST BE RECORDED IN DAYS, WEEKS OR MONTHS.<br>IF 12 MONTHS (ONE YEAR) OR MORE, ANSWER MUST BE RECORDED IN YEARS.	DAYS AGO . . . . 1 [ ][ ]<br>WEEKS AGO . . . . 2 [ ][ ]<br>MONTHS AGO . . . . 3 [ ][ ]<br>YEARS AGO . . . . 4 [ ][ ]	

NO.	QUESTIONS AND FILTERS	CODING CATEGORIES	SKIP
628	PRESENCE OF OTHERS DURING THIS SECTION	YES NO<br>CHILDREN <10 . . . . 1 2<br>MALE ADULTS . . . . 1 2<br>FEMALE ADULTS 1 2	
629	Do you know of a place where a person can get male condoms?	YES . . . . 1<br>NO 2	 → 701
630	Where is that?<br><br>Any other place?<br><br>PROBE TO IDENTIFY EACH TYPE OF SOURCE.<br><br>IF UNABLE TO DETERMINE IF PUBLIC OR PRIVATE SECTOR, WRITE THE NAME OF THE PLACE.<br><br>____________________<br>(NAME OF PLACE(S))	PUBLIC SECTOR<br>GOVT. HOSPITAL (NATIONAL, REGIONAL, PROVINCIAL OR DISTRICT) . . . . A<br>CHC/POLYCLINIC . . . . B<br>BASIC HEALTH CENTER . . . . C<br>HEALTH SUB-CENTER . . . . D<br>HEALTH POST/SUB-HEALTH POST E<br>COMMUNITY HEALTH WORKER . . . F<br>MOBILE CLINIC . . . . G<br>OTHER PUBLIC SECTOR __________ H<br>(SPECIFY)<br>NON-GOVERNMENT SECTOR<br>MARIE STOPES . . . . I<br>RED CROSS SOCIETY . . . . J<br>AFGA . . . . K<br>OTHER NGO SECTOR __________ L<br>(SPECIFY)<br>PRIVATE MEDICAL SECTOR<br>PRIVATE HOSPITAL/CLINIC . . . . . M<br>PHARMACY . . . . N<br>PRIVATE DOCTOR . . . . O<br>FIELDWORKER . . . . P<br>OTHER PRIVATE MEDICAL SECTOR __________ Q<br>(SPECIFY)<br>OTHER SOURCE<br>CHARITY/FOUNDATIONS . . . . R<br>REFUGEE CAMP . . . . S<br>SHOP . . . . T<br>FRIENDS/RELATIVES . . . . U<br>OTHER __________ X<br>(SPECIFY)	
631	If you wanted to, could you yourself get a condom?	YES . . . . 1<br>NO . . . . 2<br>DON'T KNOW/UNSURE 8	

SECTION 7. FERTILITY PREFERENCES

NO.	QUESTIONS AND FILTERS	CODING CATEGORIES	SKIP
701	CHECK 304:<br>NEITHER STERILIZED ☐ ↓ HE OR SHE STERILIZED ☐ →		712
702	CHECK 226:<br>PREGNANT ☐ ↓ NOT PREGNANT OR UNSURE ☐ →		704
703	Now I have some questions about the future. After the child you are expecting now, would you like to have another child, or would you prefer not to have any more children?	HAVE ANOTHER CHILD . . . . 1<br>NO MORE . . . . 2<br>UNDECIDED/DON'T KNOW 8	→ 705<br><br>→ 711
704	Now I have some questions about the future. Would you like to have (a/another) child, or would you prefer not to have any (more) children?	HAVE (A/ANOTHER) CHILD . . . . 1<br>NO MORE/NONE . . . . 2<br>SAYS SHE CAN'T GET PREGNANT 3<br>UNDECIDED/DON'T KNOW 8	 → 707<br>→ 712<br>→ 710
705	CHECK 226:<br>NOT PREGNANT OR UNSURE ☐ ↓ How long would you like to wait from now before the birth of (a/another) child?<br>PREGNANT ☐ ↓ After the birth of the child you are expecting now, how long would you like to wait before the birth of another child?	MONTHS . . . . 1 ☐☐<br>YEARS . . . . 2 ☐☐<br>SOON/NOW . . . . 993<br>SAYS SHE CAN'T GET PREGNANT 994<br>OTHER ______________________ 996<br>(SPECIFY)<br>DON'T KNOW 998	 <br>→ 710<br>→ 712<br><br>→ 710
706	CHECK 226:<br>NOT PREGNANT OR UNSURE ☐ ↓ PREGNANT ☐ →		711
707	CHECK 303: USING A CONTRACEPTIVE METHOD?<br>NOT CURRENTLY USING ☐ ↓ CURRENTLY USING ☐ →		712
708	CHECK 705:<br>NOT ASKED ☐ ↓ 24 OR MORE MONTHS OR 02 OR MORE YEARS ☐ ↓ 00-23 MONTHS OR 00-01 YEAR ☐ →		711

NO.	QUESTIONS AND FILTERS	CODING CATEGORIES	SKIP
709	CHECK 704:<br><br>WANTS TO HAVE A/ANOTHER CHILD ☐ → You have said that you do not want (a/another) child soon. Can you tell me why you are not using a method to prevent pregnancy? Any other reason?<br><br>WANTS NO MORE/ NONE ☐ → You have said that you do not want any (more) children. Can you tell me why you are not using a method to prevent pregnancy? Any other reason?<br><br>RECORD ALL REASONS MENTIONED.	FERTILITY-RELATED REASONS<br>NOT HAVING SEX . . . . B<br>INFREQUENT SEX . . . . C<br>MENOPAUSAL/HYSTERECTOMY D<br>CAN'T GET PREGNANT . . . . E<br>NOT MENSTRUATED SINCE LAST BIRTH . . . . F<br>BREASTFEEDING . . . . G<br>UP TO GOD/FATALISTIC . . . . H<br><br>OPPOSITION TO USE<br>RESPONDENT OPPOSED . . . . I<br>HUSBAND/PARTNER OPPOSED . . . J<br>OTHERS OPPOSED . . . . K<br>RELIGIOUS PROHIBITION . . . . L<br><br>LACK OF KNOWLEDGE<br>KNOWS NO METHOD . . . . M<br>KNOWS NO SOURCE . . . . N<br><br>METHOD-RELATED REASONS<br>SIDE EFFECTS/HEALTH CONCERNS . . . . O<br>LACK OF ACCESS/TOO FAR . . . . . P<br>COSTS TOO MUCH . . . . Q<br>PREFERRED METHOD NOT AVAILABLE . . . . R<br>NO METHOD AVAILABLE . . . . S<br>INCONVENIENT TO USE . . . . T<br>INTERFERES WITH BODY'S NORMAL PROCESSES . . . . U<br><br>OTHER ______________________ X<br>(SPECIFY)<br>DON'T KNOW . . . . Z	
710	CHECK 303: USING A CONTRACEPTIVE METHOD?<br><br>NOT ASKED ☐ ↓<br>NO, NOT CURRENTLY USING ☐ ↓<br>YES, CURRENTLY USING ☐		→ 712
711	Do you think you will use a contraceptive method to delay or avoid pregnancy at any time in the future?	YES . . . . 1<br>NO . . . . 2<br>DON'T KNOW 8	
712	CHECK 216:<br><br>HAS LIVING CHILDREN ☐ → If you could go back to the time you did not have any children and could choose exactly the number of children to have in your whole life, how many would that be?<br><br>NO LIVING CHILDREN ☐ → If you could choose exactly the number of children to have in your whole life, how many would that be?<br><br>PROBE FOR A NUMERIC RESPONSE.	NONE . . . . 00<br><br>NUMBER . . . . [ ][ ]<br><br>OTHER ______________________ 96<br>(SPECIFY)	→ 714<br><br><br><br>→ 714

NO.	QUESTIONS AND FILTERS	CODING CATEGORIES	SKIP
713	How many of these children would you like to be boys, how many would you like to be girls and for how many would it not matter if it's a boy or a girl?	BOYS / GIRLS / EITHER<br>NUMBER [ ][ ] [ ][ ] [ ][ ]<br>OTHER ________ 96<br>(SPECIFY)	
714	In the last few months have you:<br>Heard about family planning on the radio?<br>Seen anything about family planning on the television?<br>Read about family planning in a newspaper or magazine?<br>Seen or read about family planning in internet?<br>Read about family planning in billboard?<br>Heard from health professionals?<br>Heard from local community leaders?	YES NO<br>RADIO . . . . 1 2<br>TELEVISION . . . . 1 2<br>NEWSPAPER OR MAGAZINE . . . 1 2<br>INTERNET . . . . 1 2<br>BILLBOARD . . . . 1 2<br>HEALTH PROFESSIONALS . . . . . 1 2<br>LOCAL LEADERS 1 2	
716	CHECK 601:<br>YES, CURRENTLY MARRIED [ ]↓<br>NO, NOT IN UNION [ ] → 801		801
717	CHECK 303: USING A CONTRACEPTIVE METHOD?<br>CURRENTLY USING [ ]↓<br>NOT CURRENTLY USING OR NOT ASKED [ ] → 720		720
718	Would you say that using contraception is mainly your decision, mainly your husband's decision, or did you both decide together?	MAINLY RESPONDENT . . . . 1<br>MAINLY HUSBAND . . . . . 2<br>JOINT DECISION . . . . 3<br>OTHER ________ 6<br>(SPECIFY)	
719	CHECK 304:<br>NEITHER STERILIZED [ ]↓<br>HE OR SHE STERILIZED [ ] → 801		801
720	Does your husband want the same number of children that you want, or does he want more or fewer than you want?	SAME NUMBER . . . . 1<br>MORE CHILDREN . . . . 2<br>FEWER CHILDREN . . . . 3<br>DON'T KNOW 8	

SECTION 8. HUSBAND'S BACKGROUND AND WOMAN'S WORK

NO.	QUESTIONS AND FILTERS	CODING CATEGORIES	SKIP
801	CHECK 601:<br>CURRENTLY MARRIED ☐ ↓ FORMERLY MARRIED ☐		→ 803
802	How old was your husband on his last birthday?	AGE IN COMPLETED YEARS ☐☐	
803	Did your (last) husband ever attend school?	YES . . . . . 1<br>NO 2	 → 806
803A	What type of school (Madrassa) has he attended?	SCHOOL . . . . . 1<br>MADRASSA 2	
804	What was the highest level of school he attended: primary, secondary, or higher?	PRIMARY . . . . . 1<br>SECONDARY . . . . . 2<br>HIGHER . . . . . 3<br>DON'T KNOW 8	 <br><br>→ 806
805	What was the highest grade he completed at that level?<br>IF COMPLETED LESS THAN GRADE ONE, RECORD '00'.	GRADE . . . . . ☐☐<br>DON'T KNOW 98	
806	CHECK 801:<br>CURRENTLY MARRIED ☐ ↓ What is your husband's occupation? That is, what kind of work does he mainly do?<br>FORMERLY MARRIED ☐ ↓ What was your (last) husband's occupation? That is, what kind of work did he mainly do?	______ ☐☐☐<br>______<br>______	
807	Aside from your own housework, have you done any work in the last seven days?	YES . . . . . 1<br>NO 2	→ 811
808	As you know, some women take up jobs for which they are paid in cash or kind. Others sell things, have a small business or work on the family farm or in the family business.<br>In the last seven days, have you done any of these things or any other work?	YES . . . . . 1<br>NO 2	→ 811
809	Although you did not work in the last seven days, do you have any job or business from which you were absent for leave, illness, vacation, maternity leave, or any other such reason?	YES . . . . . 1<br>NO 2	→ 811
810	Have you done any work in the last 12 months?	YES . . . . . 1<br>NO 2	 → 815
811	What is your occupation, that is, what kind of work do you mainly do?	______ ☐☐☐<br>______<br>______	
812	Do you do this work for a member of your family, for someone else, or are you self-employed?	FOR FAMILY MEMBER . . . . . 1<br>FOR SOMEONE ELSE . . . . . 2<br>SELF-EMPLOYED 3	

NO.	QUESTIONS AND FILTERS	CODING CATEGORIES	SKIP
813	Do you usually work throughout the year, or do you work seasonally, or only once in a while?	THROUGHOUT THE YEAR . . . . 1<br>SEASONALLY/PART OF THE YEAR 2<br>ONCE IN A WHILE 3	
814	Are you paid in cash or kind for this work or are you not paid at all?	CASH ONLY . . . . 1<br>CASH AND KIND . . . . 2<br>IN KIND ONLY . . . . 3<br>NOT PAID 4	
815	CHECK 601:<br>CURRENTLY MARRIED ☐ ↓ NOT IN UNION ☐ →		823
816	CHECK 814:<br>CODE 1 OR 2 CIRCLED ☐ ↓ OTHER ☐ →		819
817	Who usually decides how the money you earn will be used: you, your husband, or you and your husband jointly?	RESPONDENT . . . . 1<br>HUSBAND . . . . 2<br>RESPONDENT AND<br>HUSBAND JOINTLY . . . 3<br>OTHER ______________________ 6<br>(SPECIFY)	
818	Would you say that the money that you earn is more than what your husband earns, less than what he earns, or about the same?	MORE THAN HIM . . . . 1<br>LESS THAN HIM . . . . 2<br>ABOUT THE SAME . . . . 3<br>HUSBAND HAS<br>NO EARNINGS . . . . 4<br>DON'T KNOW 8	 <br><br><br>→ 820
819	Who usually decides how your husband's earnings will be used: you, your husband, or you and your husband jointly?	RESPONDENT . . . . 1<br>HUSBAND . . . . 2<br>RESPONDENT AND<br>HUSBAND JOINTLY . . . . 3<br>HUSBAND HAS<br>NO EARNINGS . . . . 4<br>OTHER ______________________ 6<br>(SPECIFY)	
820	Who usually makes decisions about health care for yourself: you, your husband, you and your husband jointly, or someone else?	RESPONDENT . . . . 1<br>HUSBAND . . . . 2<br>RESPONDENT AND<br>HUSBAND JOINTLY . . . . 3<br>SOMEONE ELSE . . . . 4<br>OTHER 6	
821	Who usually makes decisions about making major household purchases?	RESPONDENT . . . . 1<br>HUSBAND . . . . 2<br>RESPONDENT AND<br>HUSBAND JOINTLY . . . . 3<br>SOMEONE ELSE . . . . 4<br>OTHER 6	
822	Who usually makes decisions about visits to your family or relatives?	RESPONDENT . . . . 1<br>HUSBAND . . . . 2<br>RESPONDENT AND<br>HUSBAND JOINTLY . . . . 3<br>SOMEONE ELSE . . . . 4<br>OTHER 6	

NO.	QUESTIONS AND FILTERS	CODING CATEGORIES	SKIP
823	Do you own this or any other house either alone or jointly with someone else?	ALONE ONLY .... 1<br>JOINTLY ONLY .... 2<br>BOTH ALONE AND JOINTLY .... 3<br>DOES NOT OWN 4	
824	Do you own any land either alone or jointly with someone else?	ALONE ONLY .... 1<br>JOINTLY ONLY .... 2<br>BOTH ALONE AND JOINTLY .... 3<br>DOES NOT OWN 4	
825	PRESENCE OF OTHERS AT THIS POINT (PRESENT AND LISTENING, PRESENT BUT NOT LISTENING, OR NOT PRESENT)	PRES./LISTEN. / PRES./NOT LISTEN. / NOT PRES.<br>CHILDREN < 10 ..... 1 2 3<br>HUSBAND .... 1 2 3<br>OTHER MALES ..... 1 2 3<br>OTHER FEMALES ... 1 2 3	
826	In your opinion, is a husband justified in hitting or beating his wife in the following situations:<br><br>If she goes out without telling him?<br>If she neglects the children?<br>If she argues with him?<br>If she refuses to have sex with him?<br>If she burns the food?	YES NO DK<br><br>GOES OUT .... 1 2 8<br>NEGL. CHILDREN ... 1 2 8<br>ARGUES .... 1 2 8<br>REFUSES SEX ..... 1 2 8<br>BURNS FOOD 1 2 8	

NO.	QUESTIONS AND FILTERS	CODING CATEGORIES	SKIP
901	Now I would like to talk about something else. Have you ever heard of an illness called AIDS?	YES . . . 1<br>NO . . . 2	 → 937
902	Can people reduce their chance of getting HIV by having just one uninfected sex partner who has no other sex partners?	YES . . . 1<br>NO . . . 2<br>DON'T KNOW . . . 8	
903	Can people get HIV from mosquito bites?	YES . . . 1<br>NO . . . 2<br>DON'T KNOW . . . 8	
904	Can people reduce their chance of getting HIV by using a condom every time they have sex?	YES . . . 1<br>NO . . . 2<br>DON'T KNOW . . . 8	
905	Can people get HIV by sharing food with a person who has AIDS?	YES . . . 1<br>NO . . . 2<br>DON'T KNOW . . . 8	
906	Can people get HIV because of witchcraft or other supernatural means?	YES . . . 1<br>NO . . . 2<br>DON'T KNOW . . . 8	
907	Is it possible for a healthy-looking person to have HIV?	YES . . . 1<br>NO . . . 2<br>DON'T KNOW . . . 8	
908	Can HIV be transmitted from a mother to her baby:<br><br>During pregnancy?<br>During delivery?<br>By breastfeeding?	YES NO DK<br><br>DURING PREG. . . . . . 1 2 8<br>DURING DELIVERY . . . 1 2 8<br>BREASTFEEDING . . . 1 2 8	
909	CHECK 908:<br>AT LEAST ONE 'YES' ☐ ↓ OTHER ☐		→ 910A
910	Are there any special drugs that a doctor or a nurse can give to a woman infected with HIV to reduce the risk of transmission to the baby?	YES . . . 1<br>NO . . . 2<br>DON'T KNOW . . . 8	
910A	From where did you hear or get information about HIV/AIDS?<br><br>Any other source?	RADIO . . . A<br>TELEVISION . . . B<br>NEWSPAPER/MAGAZINE . . . C<br>POSTER/BILLBOARD . . . D<br>INTERNET . . . E<br>HEALTH PROFESSIONALS . . . F<br>RELIGIOUS INSTITUTIONS . . . G<br>SCHOOL/TEACHER . . . H<br>COMMUNITY MEETINGS . . . I<br>WORKPLACE . . . J<br>FRIENDS/RELATIVES . . . K<br><br>OTHER ____________ X<br>(SPECIFY)	

NO.	QUESTIONS AND FILTERS	CODING CATEGORIES	SKIP
926	I don't want to know the results, but have you ever been tested to see if you have HIV?	YES . . . 1<br>NO . . . 2	 → 930
927	How many months ago was your most recent HIV test?	MONTHS AGO . . . [ ][ ]<br><br>TWO OR MORE YEARS . . . 95	
928	I don't want to know the results, but did you get the results of the test?	YES . . . 1<br>NO . . . 2	
929	Where was the test done?<br><br>PROBE TO IDENTIFY THE TYPE OF SOURCE.<br><br>IF UNABLE TO DETERMINE IF PUBLIC OR PRIVATE SECTOR, WRITE THE NAME OF THE PLACE.<br><br>__________<br>(NAME OF PLACE)	PUBLIC SECTOR<br>GOVT. HOSPITAL (NATIONAL, REGIONAL, PROVINCIAL OR DISTRICT) . . . 11<br>CHC/POLYCLINIC . . . 12<br>BASIC HEALTH CENTER . . . 13<br>HEALTH SUB-CENTER . . . 14<br>HEALTH POST/SUB-HEALTH POST 15<br>STAND-ALONE VCT CENTER . . . 16<br>FAMILY PLANNING CLINIC . . . 17<br>MOBILE CLINIC . . . 18<br>COMMUNITY HEALTH WORKER . . . 19<br><br>OTHER PUBLIC SECTOR __________ 20<br>(SPECIFY)<br><br>NGO<br>MARIE STOPES . . . 21<br>RED CROSS SOCIETY . . . 22<br>AFGA . . . 23<br>OTHER NGO SECTOR __________ 26<br>(SPECIFY)<br><br>PRIVATE MEDICAL SECTOR<br>PRIVATE HOSPITAL/CLINIC/ PRIVATE DOCTOR . . . 31<br>STAND-ALONE VCT CENTER . . . 32<br>PHARMACY . . . 33<br>MOBILE CLINIC . . . 34<br>FIELDWORKER . . . 35<br>OTHER PRIVATE MEDICAL SECTOR __________ 37<br>(SPECIFY)<br>OTHER SOURCE<br>HOME . . . 41<br>CHARITY/FOUNDATIONS . . . 42<br>REFUGEE CAMP . . . 43<br><br>OTHER __________ 96<br>(SPECIFY)	(11–96) → 932

NO.	QUESTIONS AND FILTERS	CODING CATEGORIES	SKIP
930	Do you know of a place where people can go to get tested for HIV?	YES . . . . . 1<br>NO 2	 → 932
931	Where is that?<br><br>Any other place?<br><br>PROBE TO IDENTIFY EACH TYPE OF SOURCE.<br>IF UNABLE TO DETERMINE IF PUBLIC OR PRIVATE SECTOR, WRITE THE NAME OF THE PLACE.<br><br>______________________<br>(NAME OF PLACE(S))	PUBLIC SECTOR<br>GOVT. HOSPITAL (NATIONAL, REGIONAL, PROVINCIAL OR DISTRICT) . . . . . A<br>CHC/POLYCLINIC . . . . . B<br>BASIC HEALTH CENTER . . . . . C<br>HEALTH SUB-CENTER . . . . . D<br>HEALTH POST/SUB-HEALTH POST E<br>STAND-ALONE VCT CENTER . . . . . F<br>FAMILY PLANNING CLINIC . . . . . G<br>MOBILE CLINIC . . . . . H<br>COMMUNITY HEALTH WORKER . . . I<br>OTHER PUBLIC SECTOR ______ J<br>(SPECIFY)<br><br>NGO SECTOR<br>MARIE STOPES . . . . . K<br>RED CROSS SOCIETY . . . . . L<br>AFGA . . . . . M<br>OTHER NGO SECTOR ______ N<br>(SPECIFY)<br><br>PRIVATE MEDICAL SECTOR<br>PRIVATE HOSPITAL/CLINIC/ PRIVATE DOCTOR . . . . . O<br>STAND-ALONE VCT CENTER . . . . . P<br>PHARMACY . . . . . Q<br>MOBILE CLINIC . . . . . R<br>FIELDWORKER . . . . . S<br>OTHER PRIVATE MEDICAL SECTOR<br>______ T<br>(SPECIFY)<br><br>OTHER SOURCE<br>HOME . . . . . U<br>CHARITY/FOUNDATIONS . . . . . V<br>REFUGEE CAMP . . . . . W<br>OTHER ______ X<br>(SPECIFY)	
932	Would you buy fresh vegetables from a shopkeeper or vendor if you knew that this person had HIV?	YES . . . . . 1<br>NO . . . . . 2<br>DON'T KNOW 8	
933	If a member of your family got infected with HIV, would you want it to remain a secret or not?	YES, REMAIN A SECRET . . . . . 1<br>NO . . . . . 2<br>DK/NOT SURE/DEPENDS 8	
934	If a member of your family became sick with AIDS, would you be willing to care for her or him in your own household?	YES . . . . . 1<br>NO . . . . . 2<br>DK/NOT SURE/DEPENDS 8	
935	In your opinion, if a female teacher has HIV but is not sick, should she be allowed to continue teaching in the school?	SHOULD BE ALLOWED . . . . . 1<br>SHOULD NOT BE ALLOWED . . . . . 2<br>DK/NOT SURE/DEPENDS 8	

NO.	QUESTIONS AND FILTERS	CODING CATEGORIES	SKIP
936	Should children 12-14 be taught about using a condom to avoid getting AIDS?	YES . . . . 1<br>NO . . . . 2<br>DK/NOT SURE/DEPENDS 8	
937	CHECK 901:<br>HEARD ABOUT AIDS ☐ → Apart from AIDS, have you heard about other infections that can be transmitted through sexual contact?<br>NOT HEARD ABOUT AIDS ☐ → Have you heard about infections that can be transmitted through sexual contact?	YES . . . . 1<br>NO 2	
938	CHECK 613:<br>HAS HAD SEXUAL INTERCOURSE ☐ ↓<br>NEVER HAD SEXUAL INTERCOURSE ☐ → 946		946
939	CHECK 937: HEARD ABOUT OTHER SEXUALLY TRANSMITTED INFECTIONS?<br>YES ☐ ↓<br>NO ☐ → 941		941
940	Now I would like to ask you some questions about your health in the last 12 months. During the last 12 months, have you had a disease which you got through sexual contact?	YES . . . . 1<br>NO . . . . 2<br>DON'T KNOW 8	
941	Sometimes women experience a bad-smelling abnormal genital discharge.<br>During the last 12 months, have you had a bad-smelling abnormal genital discharge?	YES . . . . 1<br>NO . . . . 2<br>DON'T KNOW 8	
942	Sometimes women have a genital sore or ulcer. During the last 12 months, have you had a genital sore or ulcer?	YES . . . . 1<br>NO . . . . 2<br>DON'T KNOW 8	
943	CHECK 940, 941, AND 942:<br>HAS HAD AN INFECTION (ANY 'YES') ☐ ↓<br>HAS NOT HAD AN INFECTION OR DOES NOT KNOW ☐ → 946		946
944	The last time you had (PROBLEM FROM 940/941/942), did you seek any kind of advice or treatment?	YES . . . . 1<br>NO 2	 → 946

NO.	QUESTIONS AND FILTERS	CODING CATEGORIES	SKIP
945	Where did you go?<br><br>Any other place?<br><br>PROBE TO IDENTIFY EACH TYPE OF SOURCE.<br><br>IF UNABLE TO DETERMINE IF PUBLIC OR PRIVATE SECTOR, WRITE THE NAME OF THE PLACE.<br><br>______________________<br>(NAME OF PLACE(S))	PUBLIC SECTOR<br>GOVT. HOSPITAL (NATIONAL, REGIONAL, PROVINCIAL OR DISTRICT) . . . . A<br>CHC/POLYCLINIC . . . . B<br>BASIC HEALTH CENTER . . . . C<br>HEALTH SUB-CENTER . . . . D<br>HEALTH POST/SUB-HEALTH POST E<br>STAND-ALONE VCT CENTER . . . F<br>FAMILY PLANNING CLINIC . . . . G<br>MOBILE CLINIC . . . . H<br>COMMUNITY HEALTH WORKER I<br>OTHER PUBLIC SECTOR ______________ J<br>(SPECIFY)<br><br>NGO SECTOR<br>MARIE STOPES . . . . K<br>RED CROSS SOCIETY . . . . L<br>AFGA . . . . M<br>OTHER NGO SECTOR ______________ N<br>(SPECIFY)<br><br>PRIVATE MEDICAL SECTOR<br>PRIVATE HOSPITAL/CLINIC/ PRIVATE DOCTOR . . . . O<br>STAND-ALONE VCT CENTER . . . P<br>PHARMACY . . . . Q<br>MOBILE CLINIC . . . . R<br>FIELDWORKER . . . . S<br>OTHER PRIVATE MEDICAL SECTOR ______________ T<br>(SPECIFY)<br><br>OTHER SOURCE<br>CHARITY/FOUNDATIONS . . . . U<br>REFUGEE CAMP . . . . V<br>SHOP . . . . W<br>OTHER ______________ X<br>(SPECIFY)	
946	If a wife knows her husband has a disease that she can get during sexual intercourse, is she justified in asking that they use a condom when they have sex?	YES . . . . 1<br>NO . . . . 2<br>DON'T KNOW 8	
947	Is a wife justified in refusing to have sex with her husband when she knows he has sex with other women?	YES . . . . 1<br>NO . . . . 2<br>DON'T KNOW 8	

SECTION 10. OTHER HEALTH ISSUES

NO.	QUESTIONS AND FILTERS	CODING CATEGORIES	SKIP
1001	Now I would like to ask you some other questions relating to health matters. Have you had an injection for any reason in the last 12 months?<br>IF YES: How many injections have you had?<br>IF NUMBER OF INJECTIONS IS 90 OR MORE, OR DAILY FOR 3 MONTHS OR MORE, RECORD '90'.<br>IF NON-NUMERIC ANSWER, PROBE TO GET AN ESTIMATE.	NUMBER OF INJECTIONS . . . [ ][ ]<br>NONE 00	 → 1004
1002	Among these injections, how many were administered by a doctor, a nurse, a pharmacist, a dentist, or any other health worker?<br>IF NUMBER OF INJECTIONS IS 90 OR MORE, OR DAILY FOR 3 MONTHS OR MORE, RECORD '90'.<br>IF NON-NUMERIC ANSWER, PROBE TO GET AN ESTIMATE.	NUMBER OF INJECTIONS . . . [ ][ ]<br>NONE 00	 → 1004
1003	The last time you got an injection from a health provider, did he/she take the syringe and needle from a new, unopened package?	YES . . . . 1<br>NO . . . . 2<br>DON'T KNOW 8	
1004	Do you currently smoke cigarettes?	YES . . . . 1<br>NO 2	 → 1006
1005	In the last 24 hours, how many cigarettes did you smoke?	NUMBER OF CIGARETTES . . . . [ ][ ]	
1006	Do you currently smoke or use any (other) type of tobacco?	YES . . . . 1<br>NO 2	 → 1007A
1007	What (other) type of tobacco do you currently smoke or use?<br>RECORD ALL MENTIONED.	CHELAM . . . . A<br>CHEWING TOBACCO . . . . B<br>SNUFF . . . . C<br>OTHER ______________________ X<br>(SPECIFY)	
1007A	Do you currently use drugs?	YES . . . . 1<br>NO 2	 → 1007C
1007B	What type of drugs do you currently use?<br>RECORD ALL MENTIONED.	OPIUM . . . . A<br>HEROIN . . . . B<br>OTHER ______________________ X<br>(SPECIFY)	
1007C	Have you ever heard of an illness called tuberculosis or TB?	YES . . . . 1<br>NO 2	 → 1007G
1007D	How does tuberculosis spread from one person to another?<br>PROBE:<br>Any other ways?<br>[CIRCLE ALL MENTIONED]	THROUGH THE AIR WHEN COUGHING OR SNEEZING . . . . A<br>BY SHARING UTENSILS . . . . B<br>BY TOUCHING A PERSON WITH TB . C<br>THROUGH SHARING FOOD . . . . D<br>THROUGH SEXUAL CONTACT . . . . . E<br>THROUGH MOSQUITO BITES . . . . F<br>OTHER ____________________ X<br>SPECIFY<br>DON'T KNOW . . . . Z	
1007E	Can tuberculosis be cured?	YES . . . . 1<br>NO . . . . 2<br>DON'T KNOW 8	

NO.	QUESTIONS AND FILTERS	CODING CATEGORIES	SKIP
1007F	Have you ever been told by a doctor or nurse that you have/ had tuberculosis?	YES . . . . . 1<br>NO . . . . . 2<br>DON'T KNOW 8	
1007G	Have you ever heard of an illness called Hepatitis?	YES . . . . . 1<br>NO . . . . . 2<br>DON'T KNOW 8	2, 8 → 1008
1007H	Is there anything a person can do to avoid getting Hepatitis?	YES . . . . . 1<br>NO . . . . . 2<br>DON'T KNOW 8	2, 8 → 1007J
1007I	What can a person do to avoid getting Hepatitis?<br><br>PROBE:<br><br>Any other ways?<br><br>[CIRCLE ALL MENTIONED]	SAFE SEX . . . . . A<br>SAFE BLOOD TRANSFER . . . . . B<br>DISPOSABLE SYRINGE . . . . . C<br>AVOID CONTAMINATED FOOD/WATER . . . . . D<br>AVOID CONTACT WITH INFECTED PERSON . . . . . E<br>MAKING SURE THAT INSTRUMENTS OF DENTISTS ARE PROPERLY STERILIZED . . . . . F<br>OTHERS ______ X<br>(SPECIFY)<br><br>DON'T KNOW . . . . . Z	
1007J	Have you ever been told by a doctor or nurse that you have/ had Hepatitis?	YES . . . . . 1<br>NO . . . . . 2<br>DON'T KNOW 8	2, 8 → 1008
1007K	What type of Hepatitis were you diagnosed with?	HEPATITIS A . . . . . A<br>HEPATITIS B . . . . . B<br>HEPATITIS C . . . . . C<br><br>DON"T KNOW . . . . . Z	
1007L	Are you currently suffering from Hepatitis?	YES . . . . . 1<br>NO . . . . . 2<br>DON'T KNOW 8	2, 8 → 1008
1007M	What type of Hepatitis are you currently sufferring from?	HEPATITIS A . . . . . A<br>HEPATITIS B . . . . . B<br>HEPATITIS C . . . . . C<br><br>DON"T KNOW . . . . . Z	
1008	Many different factors can prevent women from getting medical advice or treatment for themselves. When you are sick and want to get medical advice or treatment, is each of the following a big problem or not?	BIG PROB-LEM / NOT A BIG PROB-LEM	
	Getting permission to go to the doctor?	PERMISSION TO GO . . . 1 / 2	
	Getting money needed for advice or treatment?	GETTING MONEY . . . . . 1 / 2	
	The distance to the health facility?	DISTANCE . . . . . 1 / 2	
	Not wanting to go alone?	GO ALONE . . . . . 1 / 2	

NO.	QUESTIONS AND FILTERS	CODING CATEGORIES	SKIP
1009	Are you covered by any health insurance?	YES . . . . 1<br>NO 2	 → 1101
1010	What type of health insurance are you covered by?<br><br>RECORD ALL MENTIONED.	MUTUAL HEALTH ORGANIZATION/<br>COMMUNITY-BASED HEALTH<br>INSURANCE . . . . A<br>HEALTH INSURANCE THROUGH<br>EMPLOYER . . . . B<br>SOCIAL SECURITY . . . . C<br>OTHER PRIVATELY PURCHASED<br>COMMERCIAL HEALTH INSURANCE D<br>OTHER ______________ X<br>(SPECIFY)	

NO.	QUESTIONS AND FILTERS	CODING CATEGORIES	SKIP
1101	Sometimes a woman can have a problem of constant leakage of urine or stool from her vagina during the day and night. This problem usually occurs after a difficult childbirth, but may also occur after a sexual assault or after pelvic surgery.<br><br>Have you ever experienced a constant leakage of urine or stool from your vagina during the day and night?	YES . . . . . 1<br>NO 2	1 → 1103
1102	Have you ever heard of this problem?	YES . . . . . 1<br>NO 2	1, 2 → 1201
1103	Did this problem start after you delivered a baby or had a stillbirth?	AFTER DELIVERED BABY . . . . . 1<br>AFTER HAD STILLBIRTH . . . . . 2<br>NEITHER 3	3 → 1105
1104	Did this problem start after a normal labor and delivery, or after a very difficult labor and delivery?	NORMAL LABOR/DELIVERY . . . . . 1<br>VERY DIFFICULT LABOR/DELIVERY . 2	1, 2 → 1106
1105	What do you think caused this problem?	SEXUAL ASSAULT . . . . . 1<br>PELVIC SURGERY . . . . . 2<br><br>OTHER ________ 6<br>(SPECIFY)<br>DON'T KNOW 8	8 → 1107
1106	How many days after [CAUSE OF PROBLEM FROM 1103 OR 1105] did the leakage start?<br><br>RECORD 90 IF 90 DAYS OR MORE	NUMBER OF DAYS AFTER DELIVERY/OTHER EVENT [ ][ ]	
1107	Have you sought treatment for this condition?	YES . . . . . 1<br>NO 2	1 → 1109
1108	Why have you not sought treatment?<br><br>PROBE AND RECORD ALL MENTIONED.	DO NOT KNOW CAN BE FIXED . . . . . A<br>DO NOT KNOW WHERE TO GO . . . . . B<br>TOO EXPENSIVE . . . . . C<br>TOO FAR . . . . . D<br>POOR QUALITY OF CARE . . . . . E<br>COULD NOT GET PERMISSION . . . . F<br>EMBARRASSMENT . . . . . G<br>PROBLEM DISAPPEARED . . . . . H<br><br>OTHER ________ X<br>(SPECIFY)	→ 1201
1109	From whom did you last seek treatment?	HEALTH PROFESSIONAL<br>DOCTOR . . . . . 1<br>NURSE/MIDWIFE . . . . . 2<br>OTHER PERSON<br>COMMUNITY<br>HEALTH WORKER . . . . . 3<br><br>OTHER ________ 6<br>(SPECIFY)	
1110	Did you have an operation to fix the problem?	YES . . . . . 1<br>NO 2	
1111	Did the treatment stop the leakage completely?<br><br>IF NO: Did the treatment reduce the leakage?	YES, STOPPED COMPLETELY . . . . . 1<br>NOT STOPPED BUT REDUCED . . . . . 2<br>NOT STOPPED AT ALL . . . . . 3<br>DID NOT RECEIVE TREATMENT 4	
1111A	How was your family members' support towards you when you were suffering from the problem?	EXCELLENT SUPPORT . . . . . 1<br>GOOD SUPPORT . . . . . 2<br>APPROPRIATE SUPPORT . . . . . 3<br>POOR SUPPORT . . . . . 4<br>NO SUPPORT AT ALL 5	

SECTION 12. MATERNAL MORTALITY

NO.		CODING CATEGORIES	SKIP
1201	Now I would like to ask you some questions about your brothers and sisters, that is, all of the children born to your natural mother, including those who are living with you, those living elsewhere and those who have died. How many children did your mother give birth to, including you?	NUMBER OF BIRTHS TO NATURAL MOTHER . . . . [ ][ ]	
1202	CHECK 1201: TWO OR MORE BIRTHS [ ] ↓ ONLY ONE BIRTH (RESPONDENT ONLY) [ ]		→ 1300
1203	How many births did your mother have before you were born?	NUMBER OF PRECEDING BIRTHS . . . . [ ][ ]	

		(1)	(2)	(3)	(4)	(5)	(6)
1204	What was the name given to your oldest (next oldest) brother or sister?	________	________	________	________	________	________
1205	Is (NAME) male or female?	MALE 1<br>FEMALE 2	MALE 1<br>FEMALE 2	MALE 1<br>FEMALE 2	MALE 1<br>FEMALE 2	MALE 1<br>FEMALE 2	MALE 1<br>FEMALE 2
1206	Is (NAME) still alive?	YES . . . . . 1<br>NO . . . . . 2<br>GO TO 1208<br>DK . . . . . 8<br>GO TO (2)	YES . . . 1<br>NO . . . 2<br>GO TO 1208<br>DK . . . 8<br>GO TO (3)	YES . . . 1<br>NO . . . 2<br>GO TO 1208<br>DK . . . 8<br>GO TO (4)	YES . . . 1<br>NO . . . 2<br>GO TO 1208<br>DK . . . 8<br>GO TO (5)	YES . . . 1<br>NO . . . 2<br>GO TO 1208<br>DK . . . 8<br>GO TO (6)	YES . . . 1<br>NO . . . 2<br>GO TO 1208<br>DK . . . 8<br>GO TO (7)
1207	How old is (NAME)?	[ ][ ]<br>GO TO (2)	[ ][ ]<br>GO TO (3)	[ ][ ]<br>GO TO (4)	[ ][ ]<br>GO TO (5)	[ ][ ]<br>GO TO (6)	[ ][ ]<br>GO TO (7)
1208	How many years ago did (NAME) die?	[ ][ ]	[ ][ ]	[ ][ ]	[ ][ ]	[ ][ ]	[ ][ ]
1209	How old was (NAME) when he/she died?	[ ][ ]<br>IF MALE OR DIED BEFORE 12 YEARS OF AGE GO TO (2)	[ ][ ]<br>IF MALE OR DIED BEFORE 12 YEARS OF AGE GO TO (3)	[ ][ ]<br>IF MALE OR DIED BEFORE 12 YEARS OF AGE GO TO (4)	[ ][ ]<br>IF MALE OR DIED BEFORE 12 YEARS OF AGE GO TO (5)	[ ][ ]<br>IF MALE OR DIED BEFORE 12 YEARS OF AGE GO TO (6)	[ ][ ]<br>IF MALE OR DIED BEFORE 12 YEARS OF AGE GO TO (7)
1210	Was (NAME) pregnant when she died?	YES . . . . . 1<br>GO TO 1213<br>NO 2	YES . . . 1<br>GO TO 1213<br>NO . . . 2	YES . . . 1<br>GO TO 1213<br>NO . . . 2	YES . . . 1<br>GO TO 1213<br>NO . . . 2	YES . . . 1<br>GO TO 1213<br>NO . . . 2	YES . . . 1<br>GO TO 1213<br>NO . . . 2
1211	Did (NAME) die during childbirth?	YES . . . . . 1<br>GO TO 1213<br>NO 2	YES . . . 1<br>GO TO 1213<br>NO . . . 2	YES . . . 1<br>GO TO 1213<br>NO . . . 2	YES . . . 1<br>GO TO 1213<br>NO . . . 2	YES . . . 1<br>GO TO 1213<br>NO . . . 2	YES . . . 1<br>GO TO 1213<br>NO . . . 2
1212	Did (NAME) die within two months after the end of a pregnancy or childbirth?	YES . . . . . 1<br>NO 2	YES . . . 1<br>NO . . . 2	YES . . . 1<br>NO . . . 2	YES . . . 1<br>NO . . . 2	YES . . . 1<br>NO . . . 2	YES . . . 1<br>NO . . . 2
1213	How many live born children did (NAME) give birth to during her lifetime?	[ ][ ]	[ ][ ]	[ ][ ]	[ ][ ]	[ ][ ]	[ ][ ]

IF NO MORE BROTHERS OR SISTERS, GO TO NEXT SECTION.

1204	What was the name given to your oldest (next oldest) brother or sister?	(7)<br>________	(8)<br>________	(9)<br>________	(10)<br>________	(11)<br>________	(12)<br>________
1205	Is (NAME) male or female?	MALE 1<br>FEMALE 2	MALE 1<br>FEMALE 2	MALE 1<br>FEMALE 2	MALE 1<br>FEMALE 2	MALE 1<br>FEMALE 2	MALE 1<br>FEMALE 2
1206	Is (NAME) still alive?	YES ..... 1<br>NO ..... 2<br>GO TO 1208<br>DK ..... 8<br>GO TO (8)	YES ... 1<br>NO ... 2<br>GO TO 1208<br>DK ... 8<br>GO TO (9)	YES ... 1<br>NO ... 2<br>GO TO 1208<br>DK ... 8<br>GO TO (10)	YES ... 1<br>NO ... 2<br>GO TO 1208<br>DK ... 8<br>GO TO (11)	YES ... 1<br>NO ... 2<br>GO TO 1208<br>DK ... 8<br>GO TO (12)	YES ... 1<br>NO ... 2<br>GO TO 1208<br>DK ... 8<br>GO TO (13)
1207	How old is (NAME)?	GO TO (8)	GO TO (9)	GO TO (10)	GO TO (11)	GO TO (12)	GO TO (13)
1208	How many years ago did (NAME) die?						
1209	How old was (NAME) when he/she died?	IF MALE OR DIED BEFORE 12 YEARS OF AGE GO TO (8)	IF MALE OR DIED BEFORE 12 YEARS OF AGE GO TO (9)	IF MALE OR DIED BEFORE 12 YEARS OF AGE GO TO (10)	IF MALE OR DIED BEFORE 12 YEARS OF AGE GO TO (11)	IF MALE OR DIED BEFORE 12 YEARS OF AGE GO TO (12)	IF MALE OR DIED BEFORE 12 YEARS OF AGE GO TO (13)
1210	Was (NAME) pregnant when she died?	YES ..... 1<br>GO TO 1213<br>NO 2	YES ... 1<br>GO TO 1213<br>NO ... 2	YES ... 1<br>GO TO 1213<br>NO ... 2	YES ... 1<br>GO TO 1213<br>NO ... 2	YES ... 1<br>GO TO 1213<br>NO ... 2	YES ... 1<br>GO TO 1213<br>NO ... 2
1211	Did (NAME) die during childbirth?	YES ..... 1<br>GO TO 1213<br>NO 2	YES ... 1<br>GO TO 1213<br>NO ... 2	YES ... 1<br>GO TO 1213<br>NO ... 2	YES ... 1<br>GO TO 1213<br>NO ... 2	YES ... 1<br>GO TO 1213<br>NO ... 2	YES ... 1<br>GO TO 1213<br>NO ... 2
1212	Did (NAME) die within two months after the end of a pregnancy or childbirth?	YES ..... 1<br>NO 2	YES ... 1<br>NO ... 2	YES ... 1<br>NO ... 2	YES ... 1<br>NO ... 2	YES ... 1<br>NO ... 2	YES ... 1<br>NO ... 2
1213	How many live born children did (NAME) give birth to during her lifetime?						
IF NO MORE BROTHERS OR SISTERS, GO TO NEXT SECTION.							

NO.	QUESTIONS AND FILTERS	CODING CATEGORIES	SKIP
1300	CHECK HOUSEHOLD QUESTIONNAIRE - Q.141 AND COVER PAGE OF WOMAN QUESTIONNAIRE.<br>WOMAN SELECTED FOR THIS SECTION [ ] ↓<br>WOMAN NOT SELECTED [ ] → 1333		1333
1301	CHECK FOR PRESENCE OF OTHERS:<br>DO NOT CONTINUE UNTIL PRIVACY IS ENSURED.<br>PRIVACY OBTAINED . . . . 1 ↓<br>PRIVACY NOT POSSIBLE . . . . 2 → 1332		1332
	READ TO THE RESPONDENT<br>Now I would like to ask you questions about some other important aspects of a woman's life. You may find some of these questions very personal. However, your answers are crucial for helping to understand the condition of women in Afghanistan. Let me assure you that your answers are completely confidential and will not be told to anyone and no one else in your household will know that you were asked these questions.		
1302	CHECK 601:<br>CURRENTLY MARRIED [ ] ↓<br>FORMERLY MARRIED [ ] (READ IN PAST TENSE AND USE 'LAST' WITH HUSBAND') ↓		
1303	First, I am going to ask you about some situations which happen to some women. Please tell me if these apply to your relationship with your (last) husband?		

Question		YES	NO	DK
a) He (is/was) jealous or angry if you (talk/talked) to other men?	JEALOUS	1	2	8
b) He frequently (accuses/accused) you of being unfaithful?	ACCUSES	1	2	8
c) He (does/did) not permit you to meet your female friends?	NOT MEET FRIENDS .	1	2	8
d) He (tries/tried) to limit your contact with your family?	NO FAMILY	1	2	8
e) He (insists/insisted) on knowing where you (are/were) at all times?	WHERE YOU ARE	1	2	8

1304 Now I need to ask some more questions about your relationship with your (last) husband.

A Did your (last) husband ever:

B How often did this happen during the last 12 months: often, only sometimes, or not at all?

A	EVER	OFTEN	SOME-TIMES	NOT IN LAST 12 MONTHS
a) say or do something to humiliate you in front of others?	YES 1 →<br>NO 2	1	2	3
b) threaten to hurt or harm you or someone you care about?	YES 1 →<br>NO 2 ↓	1	2	3
c) insult you or make you feel bad about yourself?	YES 1 →<br>NO 2 ↓	1	2	3

NO.	QUESTIONS AND FILTERS	CODING CATEGORIES	SKIP
1305	A Did your (last) husband ever do any of the following things to you:	B How often did this happen during the last 12 months: often, only sometimes, or not at all?	

	EVER	OFTEN	SOME-TIMES	NOT IN LAST 12 MONTHS
a) push you, shake you, or throw something at you?	YES 1 → NO 2 ↓	1	2	3
b) slap you?	YES 1 → NO 2 ↓	1	2	3
c) twist your arm or pull your hair?	YES 1 → NO 2 ↓	1	2	3
d) punch you with his fist or with something that could hurt you?	YES 1 → NO 2 ↓	1	2	3
e) kick you, drag you, or beat you up?	YES 1 → NO 2 ↓	1	2	3
f) try to choke you or burn you on purpose?	YES 1 → NO 2 ↓	1	2	3
g) threaten or attack you with a knife, gun, or other weapon?	YES 1 → NO 2 ↓	1	2	3
h) physically force you to have sexual intercourse with him when you did not want to?	YES 1 → NO 2 ↓	1	2	3
i) physically force you to perform any other sexual acts you did not want to?	YES 1 → NO 2 ↓	1	2	3
j) force you with threats or in any other way to perform sexual acts you did not want to?	YES 1 → NO 2 ↓	1	2	3

NO.	QUESTIONS AND FILTERS	CODING CATEGORIES	SKIP
1306	CHECK 1305A (a-j):<br>AT LEAST ONE 'YES' ☐ ↓	NOT A SINGLE 'YES' ☐ →	1309
1307	How long after you first (got married/started living together) with your (last) (husband/partner) did (this/any of these things) first happen?<br><br>IF LESS THAN ONE YEAR, RECORD '00'.	NUMBER OF YEARS . . . . ☐☐<br><br>BEFORE MARRIAGE 95	
1308	Did the following ever happen as a result of what your (last) husband did to you:		
	a) You had cuts, bruises, or aches?	YES . . . . 1<br>NO 2	
	b) You had eye injuries, sprains, dislocations, or burns?	YES . . . . 1<br>NO 2	
	c) You had deep wounds, broken bones, broken teeth, or any other serious injury?	YES . . . . 1<br>NO 2	

NO.	QUESTIONS AND FILTERS	CODING CATEGORIES	SKIP
1309	Have you ever hit, slapped, kicked, or done anything else to physically hurt your (last) husband at times when he was not already beating or physically hurting you?	YES . . . 1<br>NO . . . 2	 → 1311
1310	In the last 12 months, how often have you done this to your (last) husband: often, only sometimes, or not at all?	OFTEN . . . 1<br>SOMETIMES . . . 2<br>NOT AT ALL . . . 3	
1311	Does (did) your (last) husband drink alcohol?	YES . . . 1<br>NO . . . 2	 → 1313
1312	How often does (did) he get drunk: often, only sometimes, or never?	OFTEN . . . 1<br>SOMETIMES . . . 2<br>NEVER . . . 3	
1313	Are (Were) you afraid of your (last) husband: most of the time, sometimes, or never?	MOST OF THE TIME AFRAID . . . 1<br>SOMETIMES AFRAID . . . 2<br>NEVER AFRAID . . . 3	
1314	CHECK 609:<br>MARRIED MORE THAN ONCE ☐ ↓<br>MARRIED ONLY ONCE ☐ →		1316

1315	A So far we have been talking about the behavior of your (current/last) husband. Now I want to ask you about the behavior of any previous husband.	EVER	B How long ago did this last happen?<br>0 - 11 MONTHS AGO	12+ MONTHS AGO	DON'T REMEMBER
	a) Did any previous husband ever hit, slap, kick, or do anything else to hurt you physically?	YES 1 →<br>NO 2 ↓	1	2	3
	b) Did any previous husband physically force you to have intercourse or perform any other sexual acts against your will?	YES 1 →<br>NO 2	1	2	3

NO.	QUESTIONS AND FILTERS	CODING CATEGORIES	SKIP
1316	From the time you were 15 years old has anyone other than (your/any) husband hit you, slapped you, kicked you, or done anything else to hurt you physically?	YES . . . 1<br>NO . . . 2<br>REFUSED TO ANSWER/<br>NO ANSWER . . . 3	 ┐<br><br>┘→ 1319

NO.	QUESTIONS AND FILTERS	CODING CATEGORIES	SKIP
1317	Who has hurt you in this way?<br><br>Anyone else?<br><br>RECORD ALL MENTIONED.	MOTHER/STEP-MOTHER . . . . . A<br>FATHER/STEP-FATHER . . . . . B<br>SISTER/BROTHER . . . . . C<br>DAUGHTER/SON . . . . . D<br>OTHER RELATIVE . . . . . E<br>MOTHER-IN-LAW . . . . . F<br>FATHER-IN-LAW . . . . . G<br>OTHER IN-LAW . . . . . H<br>TEACHER . . . . . I<br>EMPLOYER/SOMEONE AT WORK . . . J<br>POLICE/SOLDIER . . . . . K<br><br>OTHER ______ X<br>(SPECIFY)	
1318	In the last 12 months, how often has (this person/have these persons) physically hurt you: often, only sometimes, or not at all?	OFTEN . . . . . 1<br>SOMETIMES . . . . . 2<br>NOT AT ALL 3	
1319	CHECK 201, 226, AND 230:<br>EVER BEEN PREGNANT (YES ON 201 OR 226 OR 230) ☐ ↓<br>NEVER BEEN PREGNANT ☐ →		1324A
1320	Has any one ever hit, slapped, kicked, or done anything else to hurt you physically while you were pregnant?	YES . . . . . 1<br>NO 2	 → 1324A
1321	Who has done any of these things to physically hurt you while you were pregnant?<br><br>Anyone else?<br><br>RECORD ALL MENTIONED.	CURRENT HUSBAND . . . . . A<br>MOTHER/STEP-MOTHER . . . . . B<br>FATHER/STEP-FATHER . . . . . C<br>SISTER/BROTHER . . . . . D<br>DAUGHTER/SON . . . . . E<br>OTHER RELATIVE . . . . . F<br>FORMER HUSBAND . . . . . G<br>MOTHER-IN-LAW . . . . . H<br>FATHER-IN-LAW . . . . . I<br>OTHER IN-LAW . . . . . J<br>TEACHER . . . . . K<br>EMPLOYER/SOMEONE AT WORK . . . L<br>POLICE/SOLDIER . . . . . M<br><br>OTHER ______ X<br>(SPECIFY)	
1324A	CHECK 1305A (h-j) and 1315A(b)<br>AT LEAST ONE 'YES' ☐ ↓<br>NOT A SINGLE 'YES' ☐ →		1326
1325	How old were you the first time you were forced to have sexual intercourse or perform any other sexual acts by (your/any) husband?	AGE IN COMPLETED YEARS ☐☐<br><br>DON'T KNOW 98	

NO.	QUESTIONS AND FILTERS	CODING CATEGORIES	SKIP
1326	CHECK 1305A (a-j), 1315A (a,b), 1316, AND 1320:<br><br>AT LEAST ONE 'YES' ☐ ↓ NOT A SINGLE 'YES' ☐ → 1330		1330
1327	Thinking about what you yourself have experienced among the different things we have been talking about, have you ever tried to seek help?	YES . . . . 1<br>NO 2	 → 1329
1328	From whom have you sought help?<br><br>Anyone else?<br><br>RECORD ALL MENTIONED.	OWN FAMILY . . . . A<br>HUSBAND'S FAMILY . . . . B<br>CURRENT/FORMER HUSBAND . . . . . . C<br>FRIEND . . . . D<br>NEIGHBOR . . . . E<br>RELIGIOUS LEADER . . . . F<br>DOCTOR/MEDICAL PERSONNEL . . . G<br>POLICE . . . . H<br>LAWYER . . . . I<br>SOCIAL SERVICE ORGANIZATION . . . J<br><br>OTHER ______________ X<br>(SPECIFY)	→ 1330
1329	Have you ever told any one about this?	YES . . . . 1<br>NO 2	
1330	As far as you know, did your father ever beat your mother?	YES . . . . 1<br>NO . . . . 2<br>DON'T KNOW 8	

THANK THE RESPONDENT FOR HER COOPERATION AND REASSURE HER ABOUT THE CONFIDENTIALITY OF HER ANSWERS. FILL OUT THE QUESTIONS BELOW WITH REFERENCE TO THE DOMESTIC VIOLENCE MODULE ONLY.

NO.	QUESTION		YES ONCE	YES, MORE THAN ONCE	NO
1331	DID YOU HAVE TO INTERRUPT THE INTERVIEW BECAUSE SOME ADULT WAS TRYING TO LISTEN, OR CAME INTO THE ROOM, OR INTERFERED IN ANY OTHER WAY?	HUSBAND	1	2	3
		OTHER MALE ADULT .	1	2	3
		FEMALE ADULT	1	2	3

1332 INTERVIEWER'S COMMENTS / EXPLANATION FOR NOT COMPLETING THE DOMESTIC VIOLENCE MODULE

____

____

NO.	QUESTION	CODING CATEGORIES
1333	RECORD THE TIME.	HOURS . . . . ☐☐<br>MINUTES . . . . ☐☐

INTERVIEWER'S OBSERVATIONS

TO BE FILLED IN AFTER COMPLETING INTERVIEW

COMMENTS ON SPECIFIC QUESTIONS:

ANY OTHER COMMENTS

SUPERVISOR'S OBSERVATIONS

NAME OF SUPERVISOR: ____________________ DATE: ____________________

EDITOR'S OBSERVATIONS

NAME OF EDITOR: ____________________ DATE: ____________________

INSTRUCTIONS:
ONLY ONE CODE SHOULD APPEAR IN ANY BOX.
COLUMN 1 REQUIRES A CODE IN EVERY MONTH.

INFORMATION TO BE CODED FOR EACH COLUMN

COLUMN 1: BIRTHS, PREGNANCIES, CONTRACEPTIVE USE**

B BIRTHS
P PREGNANCIES
T TERMINATIONS

0 NO METHOD
1 FEMALE STERILIZATION
2 MALE STERILIZATION
3 IUD
4 INJECTABLES
5 IMPLANTS
6 PILL
7 MALE CONDOM
K LACTATIONAL AMENORRHEA METHOD
L RHYTHM METHOD
M WITHDRAWAL
X OTHER MODERN METHOD
Y OTHER TRADITIONAL METHOD

COLUMN 2: DISCONTINUATION OF CONTRACEPTIVE USE

0 INFREQUENT SEX/HUSBAND AWAY
1 BECAME PREGNANT WHILE USING
2 WANTED TO BECOME PREGNANT
3 HUSBAND/PARTNER DISAPPROVED
4 WANTED MORE EFFECTIVE METHOD
5 SIDE EFFECTS/HEALTH CONCERNS
6 LACK OF ACCESS/TOO FAR
7 COSTS TOO MUCH
8 INCONVENIENT TO USE
F UP TO GOD/FATALISTIC
A DIFFICULT TO GET PREGNANT/MENOPAUSAL
D MARITAL DISSOLUTION/SEPARATION
X OTHER ______________________
(SPECIFY)
Z DON'T KNOW

				1	2	
	12	HUT	01			
	11	DALW	02			
	10	JADI	03			
	09	QAUS	04			
1	08	AQRAB	05			1
3	07	MIZAN	06			3
9	06	SONBOLA	07			9
4	05	ASAD	08			4
	04	SARATAN	09			
	03	JAUZA	10			
	02	SAUR	11			
	01	HAMMAL	12			
	12	HUT	13			
	11	DALW	14			
	10	JADI	15			
	09	QAUS	16			
1	08	AQRAB	17			1
3	07	MIZAN	18			3
9	06	SONBOLA	19			9
3	05	ASAD	20			3
	04	SARATAN	21			
	03	JAUZA	22			
	02	SAUR	23			
	01	HAMMAL	24			
	12	HUT	25			
	11	DALW	26			
	10	JADI	27			
	09	QAUS	28			
1	08	AQRAB	29			1
3	07	MIZAN	30			3
9	06	SONBOLA	31			9
2	05	ASAD	32			2
	04	SARATAN	33			
	03	JAUZA	34			
	02	SAUR	35			
	01	HAMMAL	36			
	12	HUT	37			
	11	DALW	38			
	10	JADI	39			
	09	QAUS	40			
1	08	AQRAB	41			1
3	07	MIZAN	42			3
9	06	SONBOLA	43			9
1	05	ASAD	44			1
	04	SARATAN	45			
	03	JAUZA	46			
	02	SAUR	47			
	01	HAMMAL	48			
	12	HUT	49			
	11	DALW	50			
	10	JADI	51			
	09	QAUS	52			
1	08	AQRAB	53			1
3	07	MIZAN	54			3
9	06	SONBOLA	55			9
0	05	ASAD	56			0
	04	SARATAN	57			
	03	JAUZA	58			
	02	SAUR	59			
	01	HAMMAL	60			
	12	HUT	61			
	11	DALW	62			
	10	JADI	63			
	09	QAUS	64			
1	08	AQRAB	65			1
3	07	MIZAN	66			3
8	06	SONBOLA	67			8
9	05	ASAD	68			9
	04	SARATAN	69			
	03	JAUZA	70			
	02	SAUR	71			
	01	HAMMAL	72			
